# Investigating the anti-inflammatory potential of *N*-amidic acid organoselenium candidates: biological assessments, molecular docking, and molecular dynamics simulations†

**DOI:** 10.1039/d4ra04762a

**Published:** 2024-10-09

**Authors:** Hanan A. Althikrallah, Saad Shaaban, Ayman Abo Elmaaty, Hussein Ba-Ghazal, Mohammed N. Almarri, Marwa Sharaky, Radwan Alnajjar, Ahmed A. Al-Karmalawy

**Affiliations:** a Department of Chemistry, College of Science, King Faisal University Al-Ahsa 31982 Saudi Arabia sibrahim@kfu.edu.sa halhekrallh@kfu.edu.sa; b Department of Chemistry, Faculty of Science, Mansoura University 35516 Mansoura Egypt; c Medicinal Chemistry Department, Faculty of Pharmacy, Port Said University Port Said 42511 Egypt; d Cancer Biology Department, Pharmacology Unit, National Cancer Institute (NCI), Cairo University Cairo Egypt; e CADD Unit, Faculty of Pharmacy, Libyan International Medical University Benghazi Libya; f Department of Pharmaceutical Chemistry, College of Pharmacy, The University of Mashreq Baghdad 10023 Iraq; g Pharmaceutical Chemistry Department, Faculty of Pharmacy, Horus University–Egypt New Damietta 34518 Egypt akarmalawy@horus.edu.eg

## Abstract

Inflammation is a complex process with many contributing factors, and it often causes pain. The pathophysiology of pain involves the release of inflammatory mediators that initiate pain sensation, as well as edema and other inflammation hallmarks. Selenium-containing compounds (OSe) are very promising for developing new medicines because they can treat many different diseases. In this study, we estimated the anti-inflammatory properties of maleanilic and succinanilic acids containing selenium (OSe). These molecules were designed by combining different strategies to enhance their anti-inflammatory properties. Hence, the anti-inflammatory impacts of compounds 8, 9, 10, and 11 were pursued using inflammatory markers COX-2, IL-1β, and IL-6. Notably, it was revealed that compounds 8, 9, 10, and 11 downregulated COX-2, IL-1β, and IL-6 by (2.01, 1.63, 2.26, and 2.05), (1.42, 1.64, 1.93, and 2.59), and (1.67, 2.54, 2.22, and 4.06)-fold changes, respectively. Moreover, molecular docking studies were conducted on compounds 8, 9, 10, and 11 to pursue their binding affinities for the COX-2 enzyme. Notably, very promising binding scores of compounds 8, 9, 10, and 11 towards the binding site of the COX-2 receptor were attained. Additionally, more accurate molecular dynamics simulations were performed for 200 ns for the docked complexes of compounds 8, 9, 10, and 11 to confirm the molecular docking findings, which ignore the protein's flexibility. Therefore, the exact stability of the *N*-amidic acids OSe compounds 8, 9, 10, and 11 towards the binding pocket of the COX-2 enzyme was examined and explained as well. Also, the MM-GBSA binding energy was calculated for equilibrated MD trajectory, and 200 snapshots were selected with a 50 ps interval for further analysis. Accordingly, the investigated compounds can be treated as prominent lead anti-inflammatory candidates for further optimization.

## Abstract 2

*N*-Amidic acid organoselenium candidates as anti-inflammatory agents.

## Graphical abstract

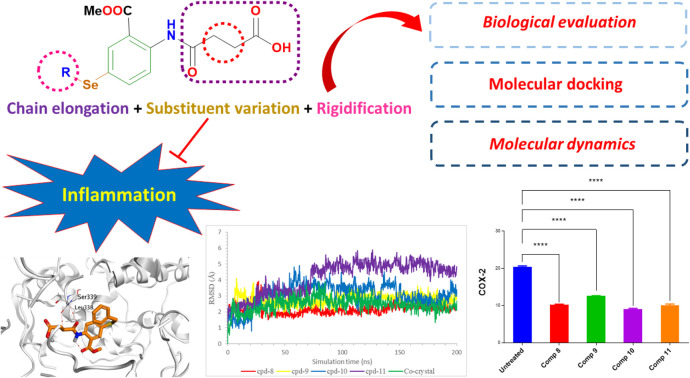

## 1. Introduction

Inflammation is a complex process with many contributing factors, and it often causes pain. It is the body's way of fighting back against invaders like germs, harmful chemicals, or physical injuries to tissues. Inflammation involves increased leakage from blood vessels, changes in cell membranes, and damage to proteins.^1,2^ The pathophysiology of pain involves unleashing inflammatory mediators that start pain sensation, as well as edema and other inflammation hallmarks.^3^

Steroids are powerful tools to fight inflammation and the pain it causes. However, their use can be tricky. Steroids can have a range of side effects, and we cannot simply stop taking them abruptly once treatment is over; they need to be tapered off gradually.^4^ Meanwhile, NSAIDs (nonsteroidal anti-inflammatory drugs) like ibuprofen, diclofenac, and indomethacin are generally safe for short-term use. However, taking them for a long time can lead to serious gastrointestinal and renal problems.^5,6^

The way the body processes arachidonic acid is essential for understanding inflammation.^7^ Arachidonic acid is metabolized to thromboxane A2 and prostaglandins by the cyclooxygenase (COX) cascade or by the 5-lipoxygenase (5-LOX) pathway upon suitable stimulation of neutrophils. Arachidonic acid is released from phospholipid membranes and transformed into prostaglandins and leukotrienes by the COX or LOX cascades.^8^

The studies focused on discovering cyclooxygenase isozymes (COX-1 and COX-2) which have contributed significantly to our understanding of inflammatory mechanisms.^9^ When inflammation occurs, macrophages move in and can release a variety of signaling molecules. Some molecules, such as interleukin (IL)-1β, IL-6, tumor necrosis factor (TNF)-α, chemokines, and interferons, act like messengers that prompt the inflammatory response. These are called pro-inflammatory mediators.^10^

The inhibition of COX-1 is responsible mainly for the unfavorable gastrointestinal and renal side effects associated with NSAIDs. To address this issue, the “coxibs” classes were developed as selective inhibitors for COX-2. However, it's worth noting that “coxibs” themselves are associated with cardiovascular severe effects.^11–13^ However, it is increasingly suggested that these adverse effects are likely to be dependent on the drug itself rather than being inherent to the entire class.^13,14^

Besides, COX-2 is overexpressed in many cancers, including colon, stomach, liver, breast, ovary, lung, and prostate cancer. Hence, drugs that block COX-2 might help prevent the incidence of these cancers.^15,16^ Accordingly, COX-2 represents a promising antitumor target, particularly in cancer cells where it is overexpressed. Therefore, there is an ongoing demand for the synthesis of novel selective COX-2 inhibitors with enhanced gastric and renal profiles, aiming to minimize consequential side effects.^3^

**Fig. 1:**
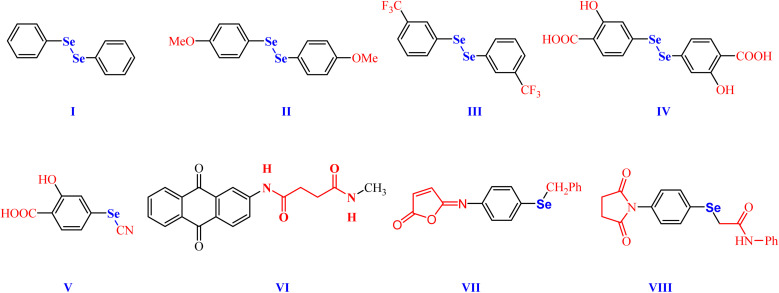
Biologically relevant amidic acid, dicarboxamide, and cyclic imides.

Research on organoselenium (OSe) compounds is gaining attention due to their potential to shield cells from damage, thanks to their antioxidant properties.^17,18^ Besides, the literature unveiled that some OSe compounds were employed as anti-inflammatory candidates.^19–23^ For instance, phenyl diselenide (PhSe)_2_I and its derivatives II and III, have shown interesting antinociceptive activities *in vivo* as well as good anti-inflammatory activities owing to their abilities to decrease proinflammatory cytokines (Fig. 1).^24–26^ Furthermore, 2-hydroxy-5-5,5′-diselanediylbis(2-hydroxybenzoic acid) IV and selenocyanatobenzoic acid V manifested good antinociceptive and anti-inflammatory activities in the croton-oil models (Fig. 1).^20,27^ Interestingly, their mode of action includes altering glutamatergic, nitrergic, and serotonergic pathways.^20,28^

On the other hand, the dicarboxamide-containing scaffolds and their cyclic imide analogues manifested potential anticoagulant and anti-inflammatory activities.^29,30^ For example, 1,3-dicarboxamide VI manifested promising antioxidant features by elevating the expression of the cytochrome P-450 enzymes in the liver (Fig. 1).^30–33^ Furthermore, the OSe-based isomaleimide VII developed in our house, has shown promising antiapoptotic activity as well as cytoprotective and antioxidant potential against oligodendrocytes (Fig. 1).^34–36^ Moreover, the OSe-containing *N*-succinimide VIII displayed good antioxidant and anticancer activity against HEPG2 cells (Fig. 1).^35–37^

We recently developed different OSe agents as potential SARS-CoV-2 M^Pro^ inhibitors.^38^ Interestingly, these compounds contain maleic and succinic acid fragments, and preliminary computational calculations data pointed out that they could bind within the active site of the 6LU7 protein pocket.^38,39^ These studies need further validation by pharmacological assessments. Furthermore, there is no available biological data for these compounds.

As this is an enormously unknown group of compounds, nothing is known about their anti-inflammatory potential and their biological targets. Furthermore, there is ample evidence in the literature indicating that OSe compounds (diorganyl diselenides, for instance, and particularly, diphenyl diselenide) have a wide range of applications as anti-inflammatory agents and some of these compounds have already entered clinical trials, *e.g.*, ebselen and ethaselen.^24–26,40^

In continuation of our previous work, our objectives were expanded further to investigate the underlying anti-inflammatory activities of these compounds to determine whether these candidates may be useful for further medicinal studies in the future. Therefore, we aim to investigate their anti-inflammatory activities using inflammation-related markers such as COX-2, IL-6, and IL-1β. Finally, molecular docking studies were performed against the target apoptotic markers. They greatly recommended the potential activity of the examined candidates to induce apoptosis as a mechanism for their antitumor activity.

### 1.1. Rational of design

**Fig. 2:**
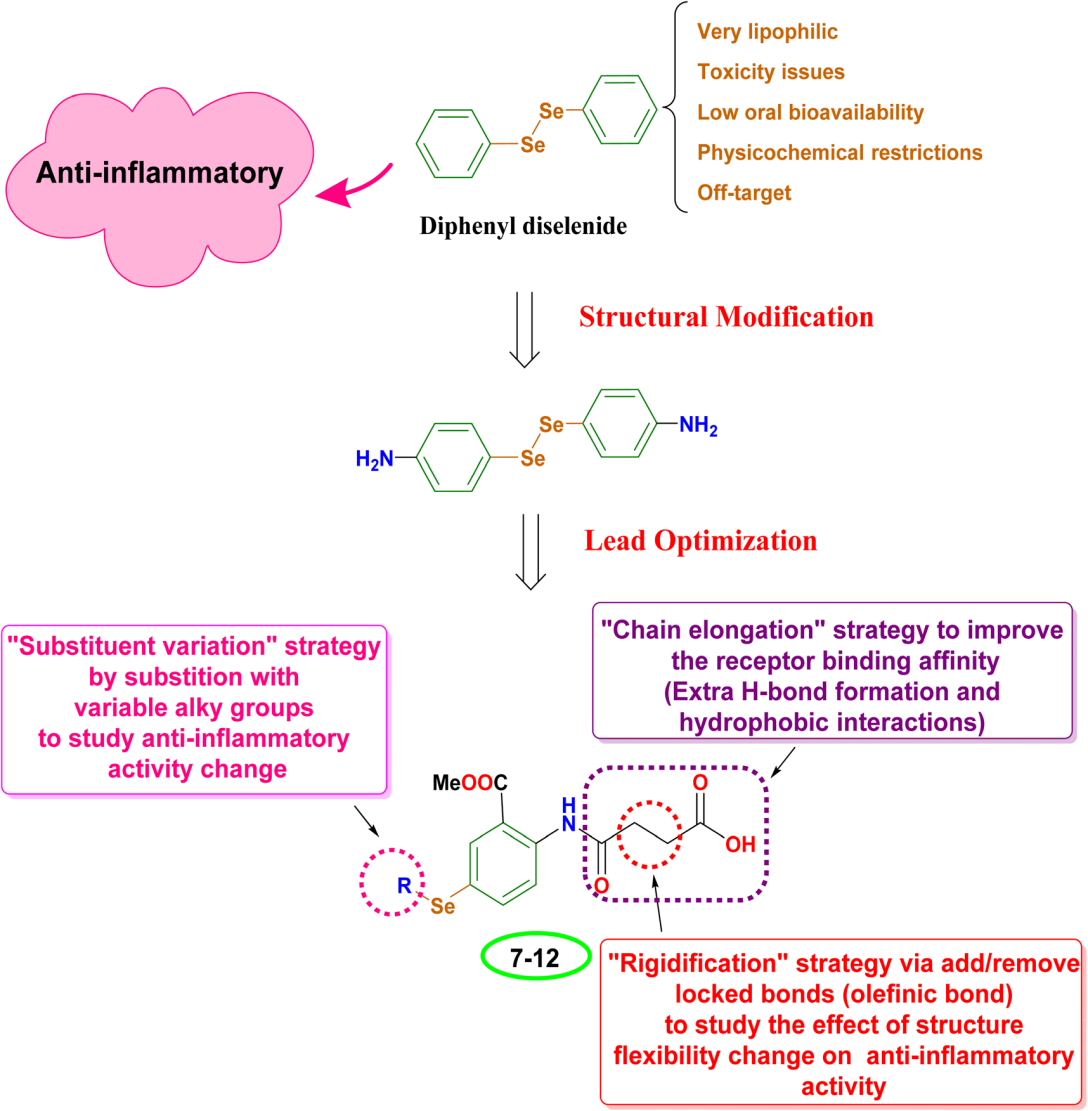
The proposed design rationale for the studied *N*-amidic acids OSe compounds as anti-inflammatory candidates.

The design aimed to enhance anti-inflammatory properties by combining various lead optimization techniques. The starting compound (PhSe)2 presented several challenges. It is highly fat-soluble, making it difficult to be absorbed orally. Additionally, it caused unwanted effects (*e.g.*, toxicity issues) due to its interactions with unintended targets, and its overall physicochemical properties limited its effectiveness.^41,42^ As a result, (PhSe)_2_ was simplified to 4-aminobenzeneselenol. Subsequently, a lead optimization tool was utilized employing various strategies, including substituent variation, chain elongation, and rigidification. In the substituent variation approach, selenium was substituted with different alkyl groups to explore changes in the anti-inflammatory activity. The 4-amino group was also replaced with an amido-butanoic acid motif as part of the chain elongation approach. This change aimed to improve how the molecule binds to its target receptor. The amido-butanoic acid motif likely achieves this by introducing additional interactions: hydrophobic interactions (attracted to other non-polar molecules) and hydrogen bonding. Furthermore, to understand how the molecule's flexibility affects its anti-inflammatory activity, rigid building blocks (olefinic bonds) were incorporated within the elongated chain of the butanoic acid group (between α and β carbons), as depicted in Fig. 2. This “rigidification approach” essentially can limit the molecule's flexibility in a specific area, changing its activity.

## 2. Results and discussion

### 2.1. Chemistry

**Scheme 1:**
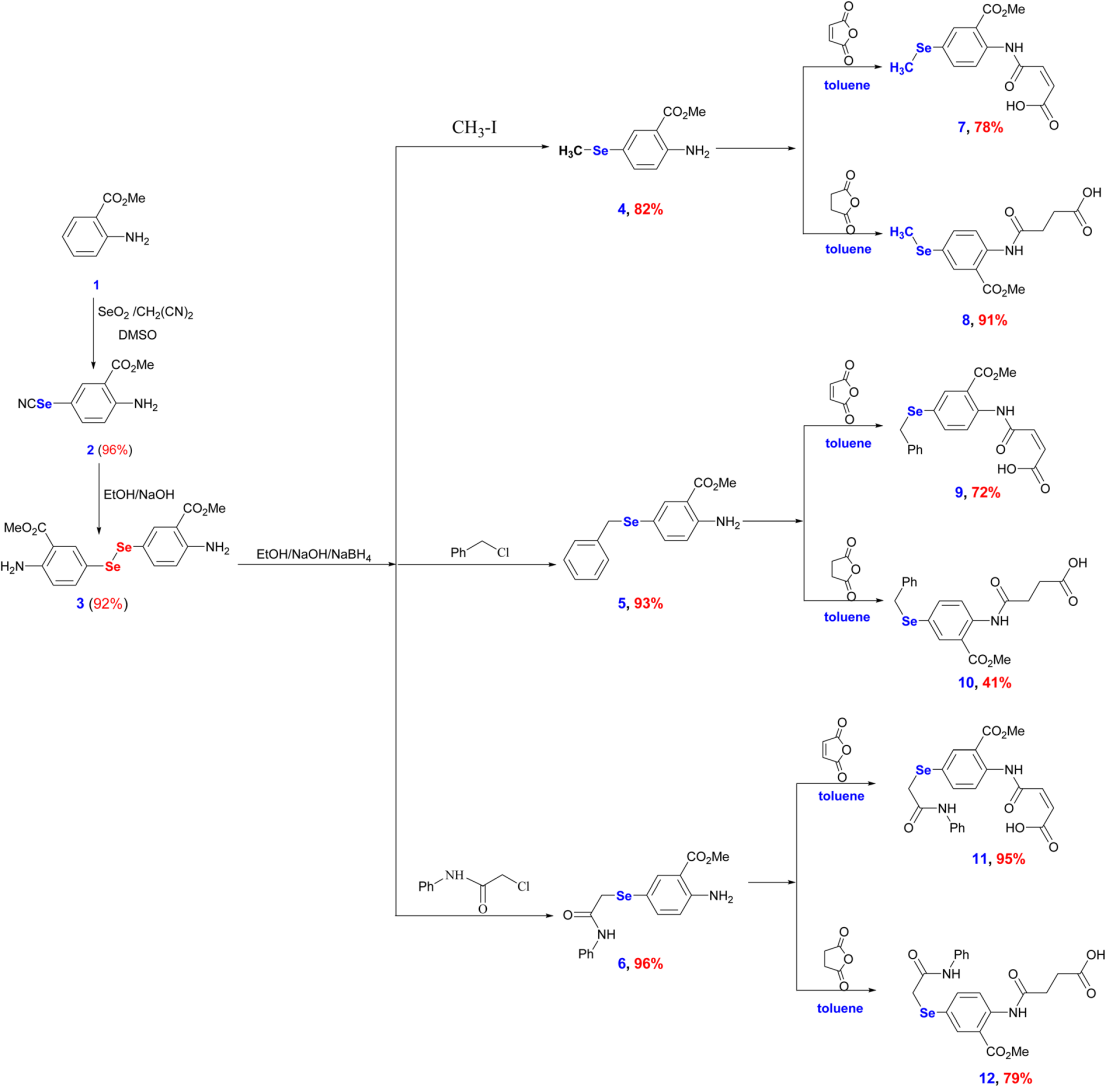
Synthesis of the target amidic acids (7–12).

The increasing interest in developing new OSe agents is driven by their privileged anti-inflammatory and antioxidant properties.^28,43,44^ Thereby, there is an urgent demand for innovative procedures that efficiently give access to highly functionalized OSe compounds and estimate their potential bioactivities. Despite the considerable advancements noticed over the last decade in the chemistry of OSe compounds, their synthesis is usually associated with several challenges.^18,43^ These include the restriction to certain reaction conditions (*e.g.*, under inert gas, dry conditions, or high temperature) as well as the incorporation of hazardous and toxic reagents.^45–47^ Hence, there is a high requirement for developing mild and simple protocols using stable OSe reagents compatible with a wide functional group. Diaryl diselenides are versatile precursors for synthesizing a wide range of multifunctional OSe intermediates, such as aryl selenide halide (ArSeX). The latter is used for the synthesis of structurally diverse selenaheterocycles.^48^ Indeed, diaryl diselenides are generally stable and safe to use and handle.^40,48,49^ Likewise, carboxamides are key scaffolds in several bioactive molecules such as peptides, pseudopeptides, enzymes, and pharmacologically active agents.^37,50^ Their potential bioactivities stem from their exceptional electronic properties as well as their ability to form hydrogen bonding. Therefore, incorporating carboxamides into the OSe scaffolds will enhance the overall anti-inflammatory properties. From a chemistry perspective, dimethyl 5,5′-diselanediylbis(2-aminobenzoate) (3) has three functional groups, *i.e.*, diselenide (Se–Se), ester (COOMe), and amino (NH_2_) groups. It can be obtained in excellent yield (92%) and high purity and is also soluble in most organic solvents.^37,39,51^ Compound 3 is synthesized in two steps, starting from methyl 2-aminobenzoate (1) by selenocyanation and subsequent hydrolysis (Scheme 1). The reduction of OSe 3 by NaBH_4_ affords reactive nucleophile sodium arylselenolate intermediate. The reaction of sodium arylselenolate with alkyl halides such as iodomethane, α-chlorotoluene, and 2-chloroacetanilide furnished the respective *para*-substituted primary aromatic OSe amines 4, 5, and 6 in very good yields (up to 96%) (Scheme 1). The nucleophilic attack of the amine functionality of the OSe compounds 4, 5, and 6 on the maleic and succinic anhydride carbonyl carbon resulted in ring opening and the subsequent formation of the *N*-amidic acids 7–12 in good yields (up to 95%) as shown in Scheme 1.

### 2.2. Biological assessments

First, the cytotoxic inhibitory concentration 50 (IC_50_) of the investigated compounds (7–12) was pursued against the human melanoma cancer (A375) cell line using the SRB assay.^52^ Their IC_50_ values were recorded as 7, 5, 4.8, 7, 5, and 17 μg mL^−1^, respectively.^53^

#### 2.2.1. Protein expression of the inflammatory-related genes

**Fig. 3:**
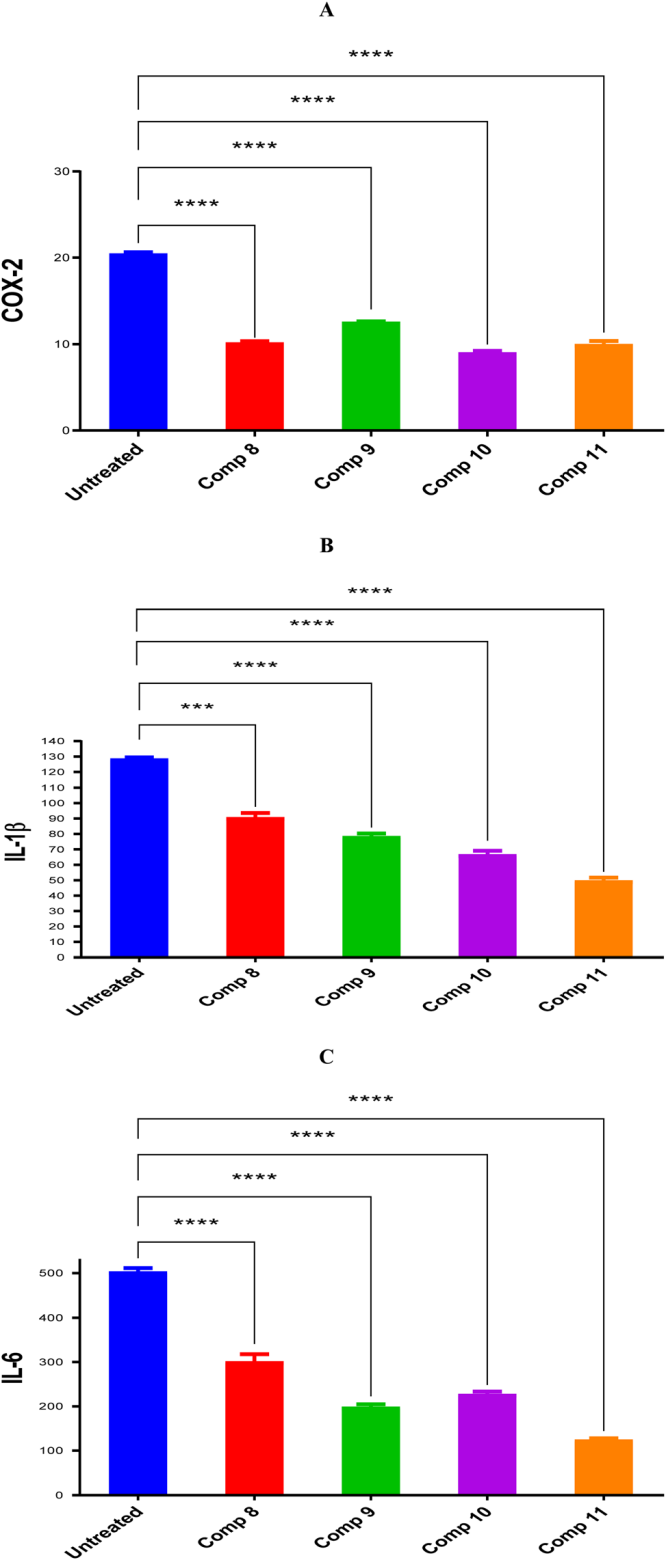
Compounds 8, 9, 10, and 11 protein expression levels for (A) COX-2, (B) IL-1β, and (C) IL-6 in comparison to untreated control cells with statistical analysis using one-way ANOVA (*p* < 0.5).

Owing to their superior cytotoxic potential, the anti-inflammatory effects of compounds 8, 9, 10, and 11 were pursued using inflammatory markers COX-2, IL-1β, and IL-6. Notably, it was revealed that compounds 8, 9, 10, and 11 downregulated COX-2 by 2.01, 1.63, 2.26, and 2.05-fold changes, respectively, as shown in Fig. 3A. Besides, it was shown that compounds 8, 9, 10, and 11 downregulated IL-1β by 1.42, 1.64, 1.93, and 2.59-fold changes, respectively, as shown in Fig. 3B. Moreover, it was displayed that compounds 8, 9, 10, and 11 downregulated IL-6 by 1.67, 2.54, 2.22, and 4.06-fold changes, respectively, as shown in Fig. 3C. Accordingly, we can assume that compound 10 has superior activity against COX-1, whereas, compound 11 has superior activity against IL-1β and IL-6.

### 2.3. *In silico* studies

#### 2.3.1. Molecular docking

To investigate their anti-inflammatory potential, *N*-amidic acids OSe compounds 8, 9, 10, and 11 were docked against the crucial inflammatory mediator (COX-2). The binding mode of the native co-crystallized inhibitor was observed to be two hydrogen bonds with Leu338 and Ser339 indicating their importance in inducing the COX-2 inhibitory potential. Moreover, the docked co-crystallized inhibitor (Score = −10.03 kcal mol^−1^ and RMSD = 0.56 Å) showed a similar binding mode with two hydrogen bonds towards Leu338 and Ser339. Besides, the validation RMSD was <2 Å, indicating the accuracy of the applied software.^54^

**Fig. 4:**
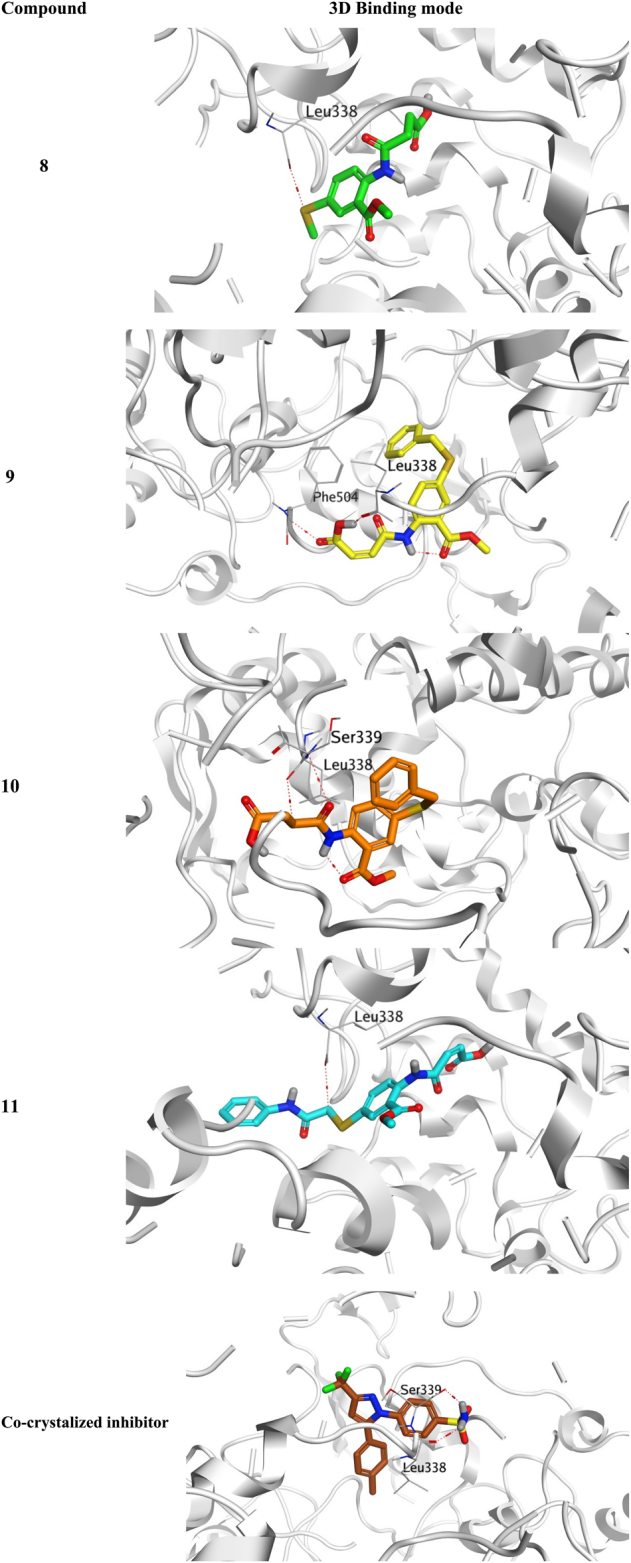
3D binding modes of compounds 8, 9, 10, and 11 within the binding pocket of COX-2 (PDB ID: 3LN1) compared to COX-2 co-crystallized inhibitor.

The binding scores of compounds 8, 9, 10, and 11 were recorded as −8.21, −8.51, −6.51, and −7.29 kcal mol^−1^ at RMSD values of 1.05, 1.25, 1.42, and 1.60 Å, respectively. Compound 8 showed a hydrogen bond with Leu338; however, compound 9 described two hydrogen bonds with Leu338 and Phe504. On the other side, compound 10 represented two hydrogen bonds with Leu338 and Ser339, and compound 11 formed a hydrogen bond with Leu338 (Fig. 4).

In conclusion, the very promising binding scores of compounds 8, 9, 10, and 11 towards the binding site of COX-2 receptor. Besides, the nearly similar binding interactions towards the crucial amino acids responsible for the antagonistic activity significantly confirm the anti-inflammatory potential of compounds 8, 9, 10, and 11.

#### 2.3.2. Molecular dynamics simulations

Additionally, more accurate molecular dynamics simulations were performed for 200 ns for the docked complexes of compounds 8, 9, 10, and 11 to confirm the molecular docking findings, which ignore the protein's flexibility. Therefore, the exact stability of the *N*-amidic acids OSe compounds 8, 9, 10, and 11 towards the binding pocket of the COX-2 enzyme could be studied and explained as well.

**Fig. 5:**
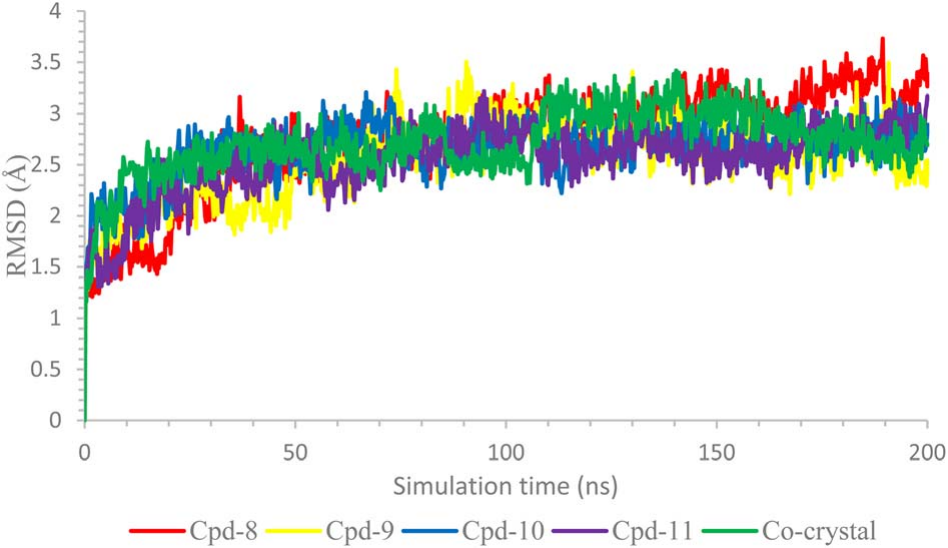
The RMSD of the ligand-COX-2 (PDB ID: 3LN1) complexes as a function of time.

The complex's stabilities were monitored with respect to their Cα initial positions as a function of time. Fig. 5 represents the RMSD of the Cα of these complexes, and as can be seen, the stabilities could be observed with low levels of fluctuations (<3.00 Å), which indicates low conformational changes within the protein structure.

**Fig. 6:**
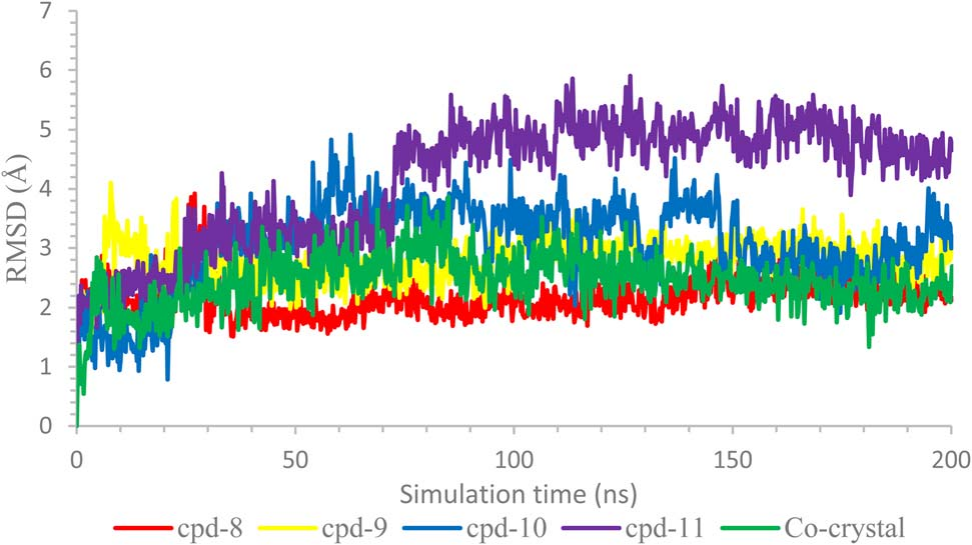
The RMSD of the ligands inside the active site of COX-2 (PDB ID: 3LN1).

Further, the behavior of ligands 8, 9, 10, and 11 inside the binding pocket of the COX-2 enzyme was monitored with respect to their original position inside the active site and was plotted as a function of time as well, Fig. 6. Briefly, the ligands' RMSD showed great stability with respect to the simulation time. Both compounds 8 and 9 showed RMSD of less than 3.00 Å, especially after 25 ns of the simulation time, indicating high stability of the ligands. However, the RMSD of compounds 10 and 11 reached 4.5 and 5.5 Å after 60 and 70 ns, respectively, indicating less but still acceptable stability behaviors. Besides, the RMSD of the co-crystallized inhibitor was recorded up to 4.00 Å, which was decreased to lower than 3.00 Å after 125 ns of the simulation time.

**Fig. 7:**
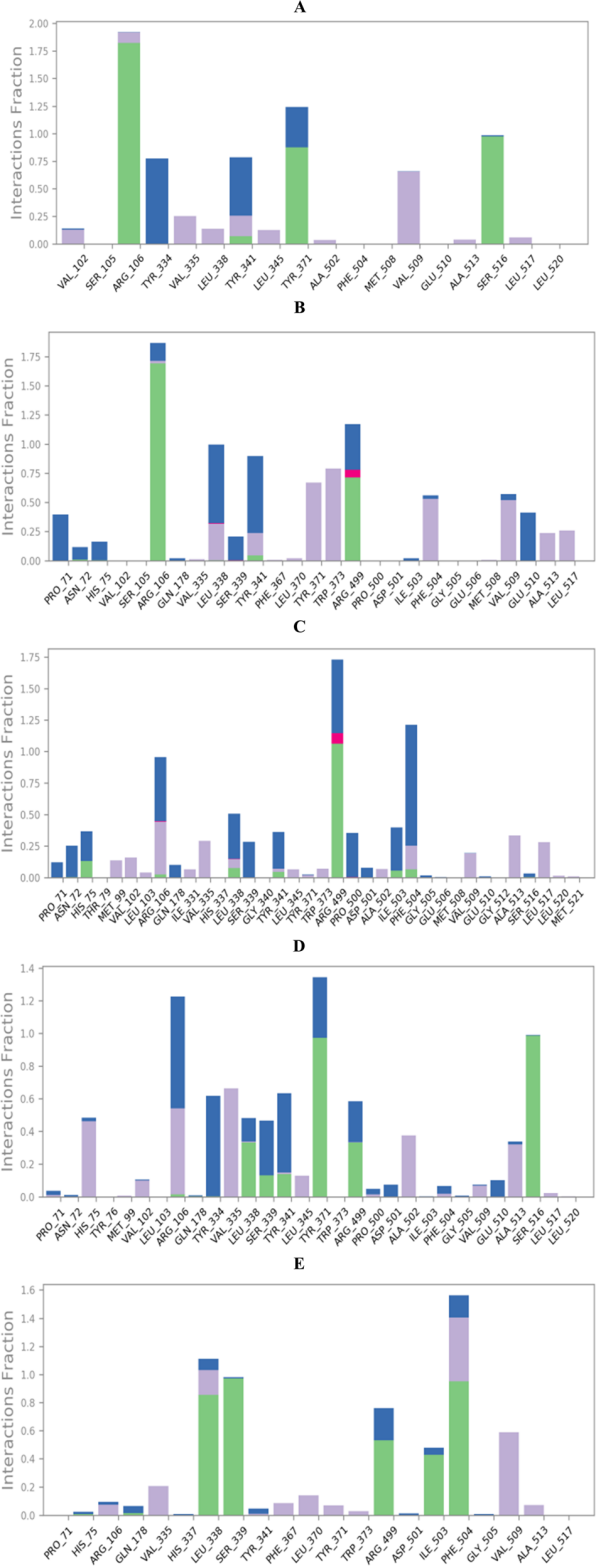
Histograms of compounds (A) 8, (B) 9, (C) 10, (D) 11, and (E) co-crystal with the active site of COX-2 (PDB ID: 3LN1).

On the other side, the protein–ligand interactions were analyzed using the histogram tool to describe the most crucial amino acids of the COX-2 binding site responsible for the binding interactions with the examined ligands (Fig. 7).

The histogram of compound 8-COX-2 complex showed that Arg106, Tyr371, and Ser516 were the most important residues to interact with at 190, 125, and 100% binding interaction, respectively. Also, the types of interactions between compound 8 and COX-2 residue were hydrogen and hydrophobic bonds (Arg106), hydrogen and water bridged hydrogen bonds (Tyr371), and hydrogen and water bridged hydrogen bonds (Ser516), Fig. 7A. Moreover, the histogram of compound 9-COX-2 complex described that Arg106, Arg499, and Leu338 were superior with 185, 125, and 100% binding interactions, respectively. The interactions between compound 9 and COX-2 were (hydrogen, hydrophobic, and water-bridged hydrogen bonds), (hydrogen, ionic, and water-bridged hydrogen bonds), and (hydrophobic, ionic, and water-bridged hydrogen bonds) for Arg106, Arg499, and Leu338, respectively (Fig. 7B). Furthermore, the compound 10-COX-2 histogram represented Arg449, Phe504, and Arg106 as the most important, with 175, 125, and 100% binding interactions, respectively. The types of interactions were (hydrogen, ionic, and water-bridged hydrogen bonds), (hydrogen, hydrophobic, and water-bridged hydrogen bonds), and (hydrogen, hydrophobic, ionic, and water-bridged hydrogen bonds) for Arg449, Phe504, and Arg106, respectively (Fig. 7C). In addition, compound 11-COX-2 histogram recorded Tyr371, Arg106, and Ser516 to be the best with 130, 120, and 100% interactions, respectively. The interactions were found to be (hydrogen and water-bridged hydrogen bonds), (hydrophobic and water-bridged hydrogen bonds), and (hydrogen and water-bridged hydrogen bonds) for Tyr371, Arg106, and Ser516, respectively (Fig. 7D). The co-crystallized inhibitor-COX-2 histogram clarified Phe504, Leu338, and Ser339 as the crucial amino acid residues for interactions with 155, 110, and 100% interactions, respectively. The interactions were as (hydrogen, hydrophobic, and water bridged hydrogen bonds), (hydrogen, hydrophobic, and water bridged hydrogen bonds), and (hydrogen and water bridged hydrogen bonds) for Phe504, Leu338, and Ser339, respectively (Fig. 7E).

**Fig. 8:**
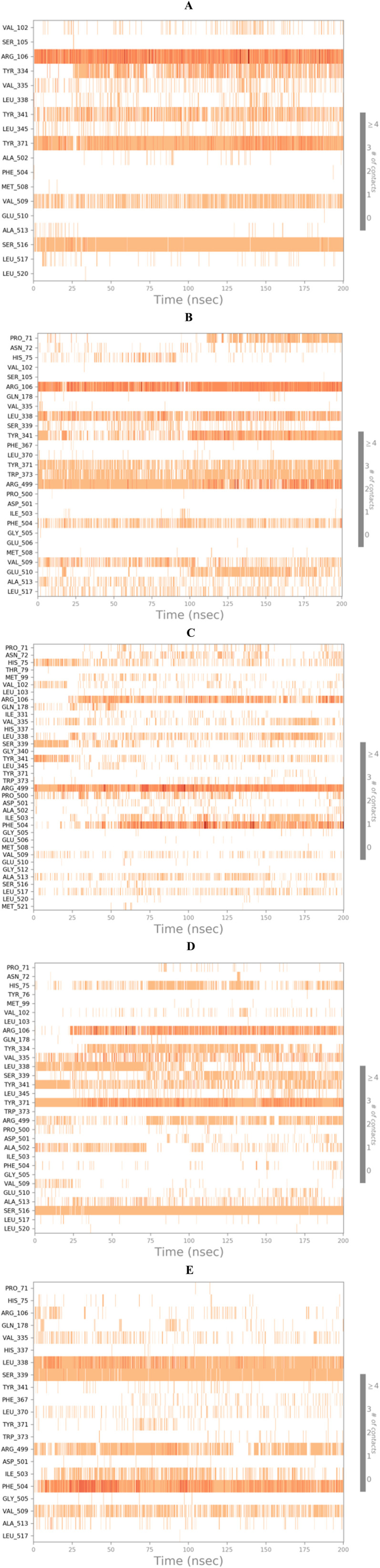
Heat maps of compounds (A) 8, (B) 9, (C) 10, (D) 11, and (E) co-crystal with the active site of COX-2 (PDB ID: 3LN1).

At the same time, the heat maps for all complexes (8, 9, 10, and 11)-COX-2, which present the interactions of the compounds with each residue as a function of time, were discussed to understand the exact time of interactions further. Herein, the heat maps of both compounds 8 and 9-COX-2 complexes showed that (Arg106, Tyr371, and Ser516) and (Arg106, Arg499, and Leu338) contributed to the binding interaction for each ligand from the start until the end of the simulation time (Fig. 8A and B), respectively. Moreover, the heat map of compound 10-COX-2 complex clarified that only Arg449 interactions were all over the 200 ns of the simulation time; however, the interactions of Arg106 and Phe504 were clearer after 25 and 30 ns of the simulation time, respectively (Fig. 8C). Further, the heat map of compound 11-COX-2 complex represented that both Tyr371 and Ser516 interactions were clear from the start until the end of the simulation time. Besides, the interactions of Arg106 appeared from 25 ns until the end of the simulation time (Fig. 8D). Finally, the heat map of the co-crystallized-COX-2 complex showed that both Leu338 and Ser339 interactions were apparent from the start until the end of the simulation time, and the interactions of Phe504 started after 5 ns until the end of the simulation time (Fig. 8E).

#### 2.3.3. MM-GBSA calculations

Finally, the average Molecular mechanics with generalized Born and surface area solvation (M-GBSA) binding energy was calculated for equilibrated MD trajectories, and 50 snapshots were selected with a 100 ps interval for further analysis. The average MM-GBSA binding energy was generated using the thermal_mmgbsa.py python script provided by Schrodinger, and the obtained results were reported in kcal mol^−1^ (Table 1).

**Table 1:** Prime MM-GBSA energies for ligands binding at the active site of COX-2 (PDB ID: 3LN1)^a^

Comp.	Δ*G*_Bind_	Coulomb	Covalent	H-bond	Lipo	Packing	Solv_GB	VdW
8	−35.92	1.51	1.00	−2.20	−10.68	−0.27	7.80	−33.08
9	−51.93	−25.77	1.18	−2.42	−19.96	−1.67	40.76	−44.05
10	−50.99	3.13	2.14	−1.26	−18.80	0.53	13.02	−49.76
11	−49.95	−18.05	1.52	−1.41	−14.39	−2.13	32.25	−47.75
Co-crystal	−60.61	−19.00	1.69	−2.03	−19.57	−1.10	21.66	−42.27

a Covalent: covalent binding energy; Coulomb: coulomb energy; Lipo: lipophilic energy; H-bond: hydrogen-bonding energy; Solv_GB: generalized Born electrostatic solvation energy; and VdW: van der Waals energy.

The binding energy was calculated as follows:Δ*G*_bind_ = Δ*E*_MM_ + Δ*G*_solv_ + Δ*G*_SA_where Δ*E*_MM_ is the difference in minimized energies between complex, ligand, and protein energy as follows:Δ*E*_MM_ = *E*_(complex)_ − *E*_(ligand)_ + *E*_(receptor)_

As can be clarified from Table 1, compounds 9, 10, and 11 showed excellent binding energies of −51.93, −50.99, and −49.95 kcal mol^−1^, respectively, in comparison to the co-crystallized ligand with Δ*G* binding of −60.61 kcal mol^−1^. Moreover, compound 9 recorded better Coulomb, H-bond, lipophilic, packing, and generalized Born electrostatic solvation energies, compared to the co-crystallized inhibitor. Furthermore, compound 10 described frontier covalent and van der waals energies compared to the co-crystallized inhibitor.

## 3. Conclusion

By combining chain elongation, substituent variation, and rigidification approaches, the designed OSe compounds can be treated as outstanding anti-inflammatory candidates. The investigated OSe compounds 8, 9, 10, and 11 exhibited remarkable downregulation of anti-inflammatory markers *i.e.* COX-2, IL-1β, and IL-6, assuring their anti-inflammatory potential. Furthermore, the very promising binding scores of compounds 8, 9, 10, and 11 towards the binding site of the COX-2 receptor after conducting molecular docking, as well as, the nearly similar binding interactions towards the crucial amino acids responsible for the antagonistic activity confirm greatly the anti-inflammatory potential of compounds 8, 9, 10, and 11. Moreover, the molecular dynamics simulations recommended the stability of the examined complexes based on the low values of the RMSDs (<3.00 Å) indicating low conformational changes and the absence of protein denaturation. Besides, the ligands' RMSD showed great stability concerning the simulation time as well. Finally, the MM-GBSA calculations clarified that compounds 9, 10, and 11 showed superior binding energies of −51.93, −50.99, and −49.95 kcal mol^−1^, respectively, in comparison to the co-crystallized ligand with Δ*G* binding of −60.61 kcal mol^−1^. Moreover, compound 9 recorded better Coulomb, H-bond, lipophilic, packing, and generalized Born electrostatic solvation energies, and compound 10 described frontier covalent and van der Waals energies, compared to the co-crystallized inhibitor. There are more opportunities for multi-disciplinary experiments and investigations involving organic and medicinal chemistry as well as pharmacology. Furthermore, the chemical structure of active *N*-amidic acid compounds 8, 9, 10, and 11 offers significant scope for diversification, and the development of more bioactive agents is, therefore, now straightforward. The latter is a promising research point for future studies of their activity *via* the design, preparation, and assessment of structural variants of *N*-amidic acid compounds 8, 9, 10, and 11. Moreover, cell-based experiments are highly needed to evaluate their exact effect on neurons and to identify their possible biological targets. The potential of developing *N*-amidic acid small OSe libraries in a simple synthetic route will significantly improve and accelerate pharmacological studies. However, it is not possible to create other derivatives and test them now. Furthermore, more extensive research and additional studies are required to evaluate their exact mode(s) of action and to discover possible intracellular biological targets, which we are already in progress. Furthermore, to set up the entire picture of OSe candidates as possible anti-inflammatory agents, this direction of research should be shifted to animal studies. We are entirely aware that a comprehensive QSAR will require a more extensive and diverse set of candidates, not only OSe compounds. A clear QSAR, in our opinion, should also include organosulfur and organotellurium analogues to perform wider screening and analysis of their selectivity. Also, to generate consistent QSAR and to get a better insight into their possible mechanism, these compounds should be expanded to include broader organoselenocyanates as well. Moreover, *N*-amidic acid organoselenium compounds 8, 9, 10, and 11 that downregulate anti-inflammatory markers propose a startup for more structural variants of organoselenium compounds to develop more efficient anti-inflammatory candidates.

## 4. Materials and methods

### 4.1. Synthesis of the *N*-amidic acids OSe compounds

The *N*-amidic acids OSe agents were synthesized following our published literature procedure (ESI, SI1†).^38,39,51,55,56^

### 4.2. Protein expression of the inflammation-related genes

The inhibitory potentials of compounds 8, 9, 10, and 11 were evaluated by measuring the protein expression of the inflammation-related genes (COX-2, IL-6, and IL-1β) in both the treated and untreated cells. These treatments were applied using the IC_50_ values of compounds 8, 9, 10, and 11 (5, 4.8, 7, and 5), respectively, toward the A375 cancer cell line.^53^ This protocol tends to measure and compare COX-2, IL-6, or IL-1β protein expression levels between the cells treated with the examined candidates and the negative control untreated cells (ESI, SI2†).

### 4.3. *In silico* studies

#### 4.3.1. Molecular docking

*N*-amidic acids OSe compounds 8, 9, 10, and 11 were docked against the crucial inflammatory marker (COX-2) using the AutoDock Vina,^57^ and visualized using the PyMOL software.^58^ Their chemical structures were copied from the ChemDraw to the working window of the drug design program to be energy minimized and corrected.^59^ The target COX-2 receptor was downloaded from (https://www.rcsb.org/structure/3LN1), corrected for errors, 3D hydrogenated, and energy minimized.^60,61^ Then, candidates 8, 9, 10, and 11 were docked toward the COX-2 receptor, and the scores and binding modes were discussed in detail.^62^

#### 4.3.2. Molecular dynamics simulations

The Desmond package of Schrödinger LLC^63,64^ was used to carry out the molecular dynamics simulations at 200 ns^61,65^ for the examined complexes of *N*-amidic acids OSe compounds (8, 9, 10, and 11). The full method was discussed in the supplementary data (ESI, SI3†).

#### 4.3.3. MM-GBSA calculations

The thermal_mmgbsa.py python script of Schrödinger LLC was applied to estimate the Molecular Mechanics Generalized Born Surface Area (MM-GBSA) energies.^66,67^ The detailed method was represented in the supplementary data (ESI, SI4†).

## Funding

This research work was funded by the Deputyship for Research & Innovation, Ministry of Education in Saudi Arabia for funding this research work through the project number INST222.

## Data availability

The data supporting this article have been included in the main manuscript and the ESI.†

## Conflicts of interest

The authors declare no conflict of interest.

## Supplementary Material

RA-014-D4RA04762A-s001
