# Supplementary material for: Investigating the anti-inflammatory potential of N-amidic acid organoselenium candidates: biological assessments, molecular docking, and molecular dynamics simulations
Source: RSC Adv. 2024 Oct 9;14(44):31990–2000. doi: 10.1039/d4ra04762a (PMC11463133; doi:10.1039/d4ra04762a)
Supplement: RA-014-D4RA04762A-s001 [file RA-014-D4RA04762A-s001.pdf]

Supplementary Information (SI) for RSC Advances.
This journal is © The Royal Society of Chemistry 2024

Supporting Information

Investigating the Anti-inflammatory Potential of *N*-Amidic Acids Organoselenium Candidates: Biological Assessments, Molecular Docking, and Molecular Dynamic Simulations

Hanan A. Althikrallah[1,*#], Saad Shaaban[1,2,*#], Ayman Abo Elmaaty[3], Hussein Ba-Ghazal[1], Mohammed N. Almarri[1], Marwa Sharaky[4], Radwan Alnajjar[5], Ahmed A. Al-Karmalawy[6,7,*]

[1] Department of Chemistry, College of Science, King Faisal University, Al-Ahsa 31982, Saudi Arabia.

[2] Department of Chemistry, Faculty of Science, Mansoura University, 35516 Mansoura, Egypt.

[3] Medicinal Chemistry Department, Faculty of Pharmacy, Port Said University, Port Said, 42511, Egypt.

[4] Cancer Biology Department, Pharmacology Unit, National Cancer Institute (NCI), Cairo University, Cairo, Egypt.

[5] CADD Unit, Faculty of Pharmacy, Libyan International Medical University, Benghazi, Libya.

[6] Department of Pharmaceutical Chemistry, College of Pharmacy, The University of Mashreq, Baghdad 10023, Iraq.

[7] Pharmaceutical Chemistry Department, Faculty of Pharmacy, Horus University–Egypt, New Damietta, 34518, Egypt.

[*] Corresponding authors:

(H. A. Althikrallah) halhekrallh@kfu.edu.sa; (S. Shaaban) sibrahim@kfu.edu.sa; (A. A. Al-Karmalawy) akarmalawy@horus.edu.eg

[#] These two authors contributed equally.

SI1. Chemistry

1.1. Material and methods

Melting points were recorded in degree centigrade on a Gallenkamp instrument using the standard open capillary method. The IR spectra were recorded on FTIR 5000 Mattson spectrophotometer. The ^{1}H- and ^{13}C-NMR spectra were recorded in DMSO–d_6 on a Varian 400 and 500 Spectrophotometer (^{1}H: 400 and 500 MHz, ^{13}C: 101 and 125 MHz) at 295 K. The chemical shifts (δ) are given in parts per million (ppm) downfield relative to tetramethyl silane (TMS). Mass Spectra were recorded on Bruker micrOTOFQ II APPI mass spectrometer. Compounds **2–6** were obtained according to our literature reports [1-2], and the new compounds **7**–**12** obtained according to our literature reports[3].

1.2. Synthesis and characterization

The synthesis of OSe maleanilic 7, 9, and 11 and succinanilic 8, 10, and 12 derivatives

Maleic or succinic anhydride (1.3 mmol) was added to OSe amine (1.0 mmol) in methylbenzene (3.0 mL). The solution was stirred for 8 hrs. Then, the formed precipitate was filtered and washed with warm methylbenzene and water. The acids were obtained in enough purity, and no further purifications were needed.

Synthesis of methyl 2-amino-5-selenocyanatobnzoate (2) [1, 4]

Methyl 2-amino-5-selenocyanatobenzoate (**2**) was synthesized from methyl 2-aminobenzoate (12.5 mmol, 1.80 g) with triselenium dicyanide prepared *in situ* from malononitrile (15 mmol, 1.00 g) and selenium dioxide (30 mmol, 3.30 g). It was isolated as reddish solid; yield:3.07 g (96%); m.p. = 118–119 °C; Rf = 0.4 (petroleum ether / ethyl acetate 4:2, *v*/*v*). IR (KBr): ν 3475 (N-H), 3366 (N-H), 2946 (C_{aliph}-H), 2842 (C_{aliph}-H), 2148 (CN), 1691 (C=O), 1551 (C=C), 1253 (C_{Ar}–N), 1085 (C–O), 910, 815 (C-H bending), 556 (C-Se), 536 (C-H rocking), 460; ^{1}H NMR (400 MHz, DMSO-d_6) δ 8.02 (s, 1H, Ar-H), 7.57 (d, J = 8.8 Hz, 1H, Ar-H), 7.08 (s, br, 2H, NH_2), 6.84 (d, J = 8.8 Hz, 1H, Ar-H), 3.82 (s, 3H, OCH_3). ^{13}C NMR (101 MHz, DMSO- d_6) δ 166.73, 152.22, 140.09, 137.94, 118.28, 109.59, 105.54, 105.47, 51.73. MS (EI, 70 ev) m/z (%) = 259.35 (M+3H, 2.39), 117 (29.02), 87 (26.6), 75 (2.70), 59 (100.0, base peak).

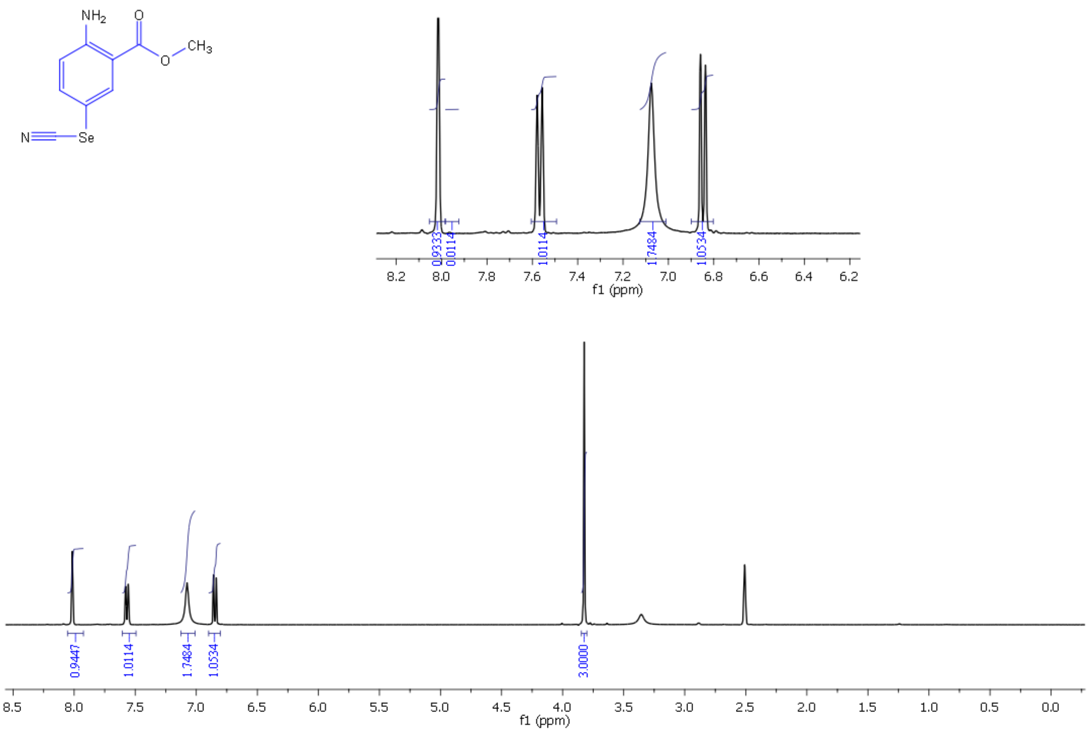

^{1}H NMR chart of compound **2**

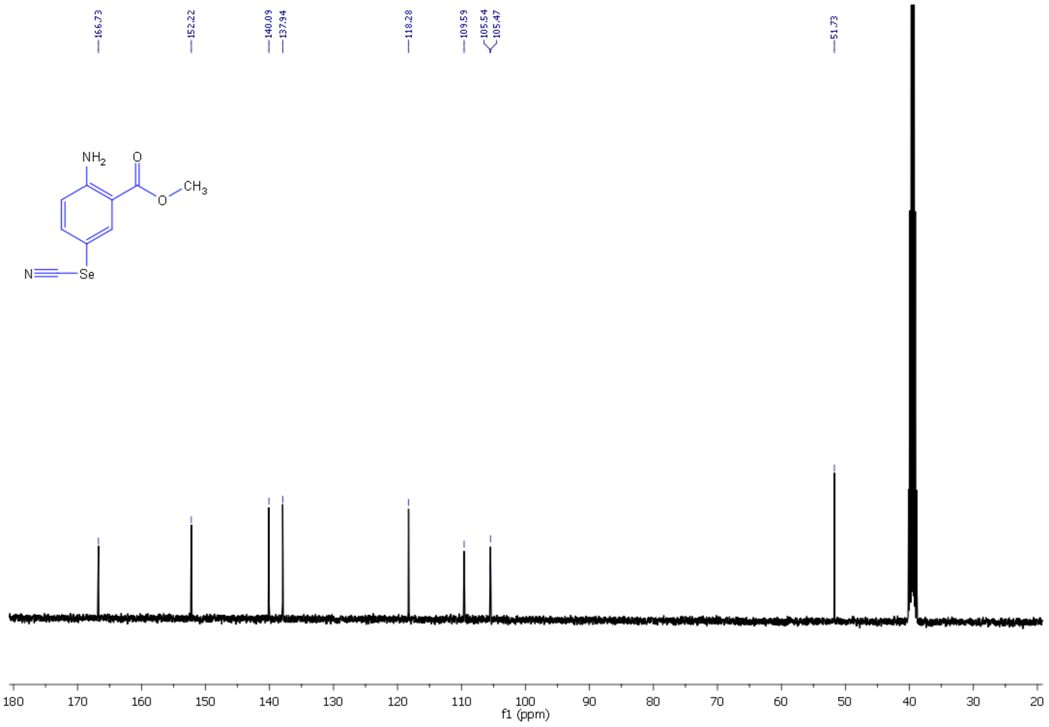

^{13}C NMR chart of compound **2**

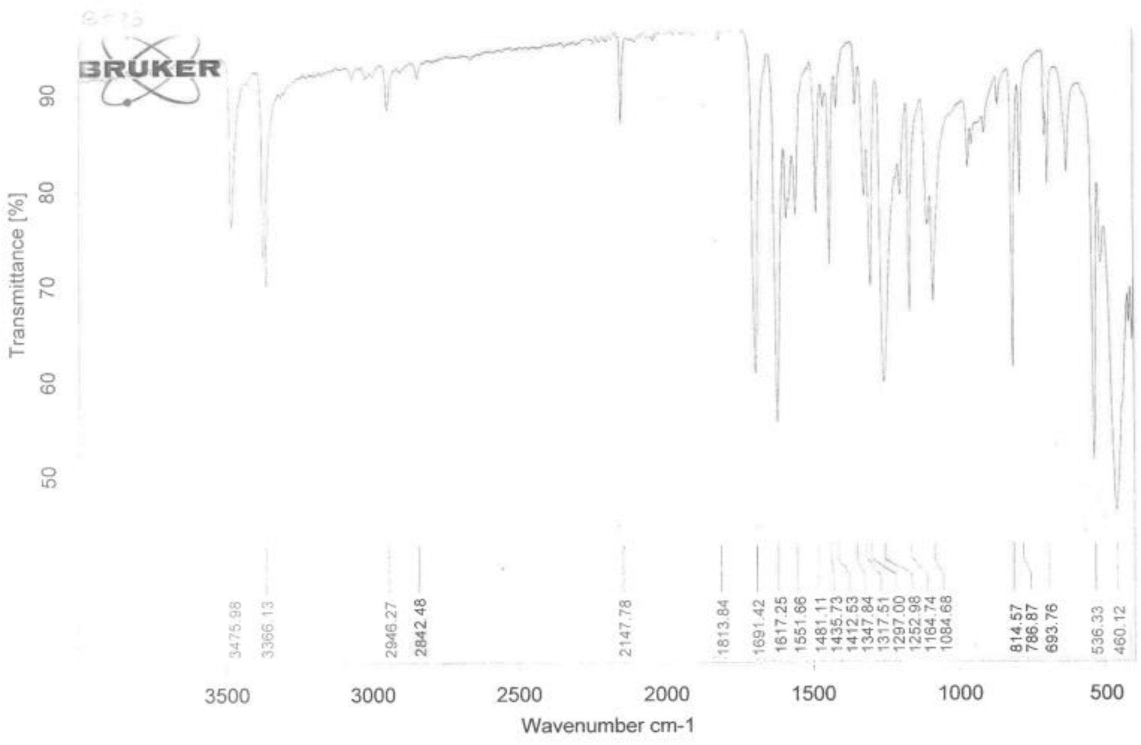

IR chart of compound **2**

06-Jan-07 19:37:16

Cairo University
Micro Analytical Center

DI Analysis
Shimadzu Qp-2010 Plus

Sample Information

Analyzed by	: Dr. Mai Younis
Analyzed	: 06/01/2007 07:26:19
Sample Name	: B2
Sample ID	:
Customer Name	: Dr.Mohamed Soliman - Science - Cairo
Data File	: C:\GCMSsolution\Data\Project1\B2.QGD
Org Data File	: C:\GCMSsolution\Data\Project1\B2.QGD
Method File	: C:\GCMSsolution\Data\Project1\High Temperature Op
Org Method File	: C:\GCMSsolution\Data\Project1\High Temperature Op
Report File	:
Tuning File	: C:\GCMSsolution\System\Tune1_default.qgt
$EndIf$Modified by	: Dr. Mai Younis
Modified	: 06/01/2007 07:32:04

Method

——— Analytical Line 1 ———

IonSourceTemp	:250.00 °C
[MS Table]	
--Group 1 - Event 1--	
Start Time	:0.00min
End Time	:10.00min
ACQ Mode	:Scan
Event Time	:0.50sec
Scan Speed	:1000
Start m/z	:50.00
End m/z	:510.00
Electron Voltage	: 70 eV
Ionization Mode	: EI

C:\GCMSsolution\Data\Project1\B2.QGD

Chromatogram B2 C:\GCMSsolution\Data\Project1\B2.QGD

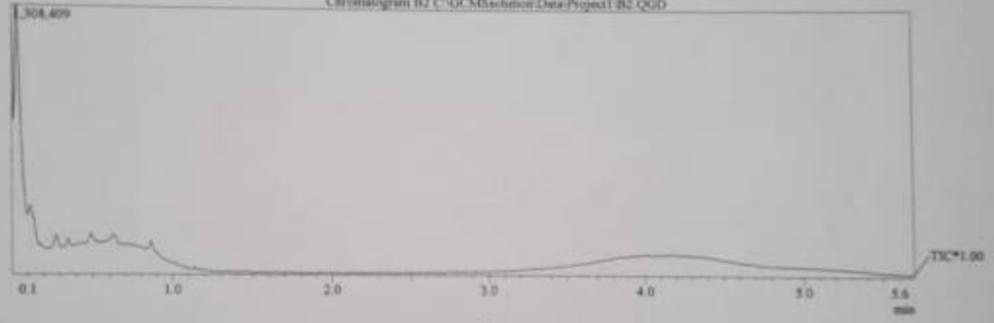

Spectrum

Line#:1 R.Time:4.1(Scan#:490)
MassPeaks:76
RawMode:Single 4.1(490) BasePeak:59(21586)
BG Mode:None Group 1 - Event 1

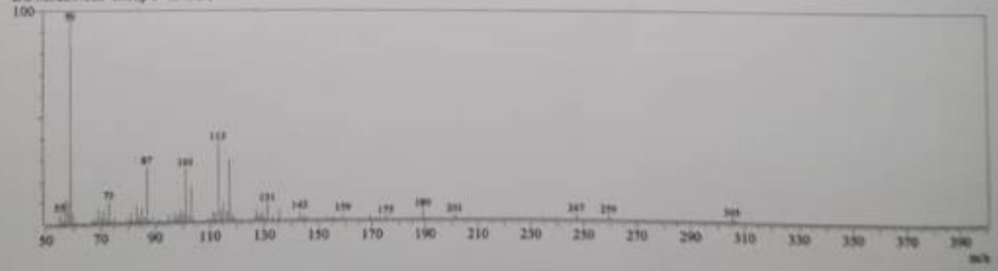

Mass Table
Line#:1 R.Time:4.1(Scan#:490)
MassPeaks:76
RawMode:Single 4.1(490) BasePeak:59(21586)
BG Mode:None Group 1 - Event 1

#	m/z	Abs. In	Rel. Int.	#	m/z	Abs. In	Rel. Int.	#	m/z	Abs. In	Rel. Int.
1	55.05	1062	4.92	4	58.05	2312	10.71	7	61.00	236	1.09
2	56.05	354	1.64	5	59.00	21586	100.00	8	67.10	468	2.17
3	57.05	2418	11.20	6	59.95	1083	5.02	9	68.15	341	1.58

1 / 2

Mass chart of compound **2**

#	m/z	Abs. In	Rel. Int.
10	69.05	1515	7.02
11	70.10	633	2.93
12	71.05	1365	6.32
13	72.05	440	2.04
14	73.00	2258	10.46
15	74.00	302	1.40
16	75.05	583	2.70
17	77.00	268	1.24
18	79.00	223	1.03
19	80.10	324	1.50
20	81.10	954	4.42
21	82.15	315	1.46
22	83.10	1628	7.54
23	84.10	481	2.23
24	85.05	1397	6.47
25	86.05	492	2.28
26	87.05	5752	26.65
27	88.00	314	1.45
28	89.05	560	2.59
29	91.10	327	1.51
30	94.10	202	0.94
31	95.10	807	3.74
32	96.10	278	1.29
33	97.10	945	4.38
34	98.15	599	2.77
35	99.10	1268	5.87
36	100.15	840	3.89
37	101.10	5503	25.49
38	102.15	699	3.24
39	103.10	3649	16.90
40	104.10	322	1.49
41	109.10	401	1.86
42	110.10	220	1.02
43	111.10	969	4.49
44	112.15	807	3.74
45	113.10	8084	37.45
46	114.10	1199	5.55
47	115.10	2001	9.27
48	116.25	936	4.34
49	117.15	6264	29.02
50	118.15	607	2.81
51	119.20	271	1.26
52	126.15	310	1.44
53	127.10	1102	5.11
54	128.15	589	2.73
55	129.10	740	3.43
56	130.15	304	1.41
57	131.10	1722	7.98
58	133.15	432	2.00
59	135.20	1132	5.24
60	143.10	908	4.21
61	144.10	210	0.97
62	145.20	492	2.28
63	153.20	234	1.08
64	155.20	281	1.30
65	159.15	662	3.07
66	169.20	334	1.55
67	171.20	204	0.95
68	173.20	202	0.94
69	175.20	346	1.60
70	189.20	1038	4.81
71	201.15	502	2.33
72	247.20	575	2.66
73	259.35	515	2.39
74	260.40	207	0.96
75	305.40	401	1.86
76	306.40	281	1.30

2 / 2

Mass chart of compound **2**

Synthesis of dimethyl 5,5'-diselanediylbis(2-aminobenzoate) (3)[1, 4]

Compound dimethyl 5,5-diselanediylbis(2-aminobenzoate) (**3**) was synthesized from methyl 2-amino-5-selenocyanatobenzoate (4 mmol, 1.00 g) and sodium hydroxide (4 mmol, 1.60 g) in anhydrous ethanol (20 mL). Dimethyl 5,5'-diselanediylbis(2-aminobenzoate) (**3**) appeared as a single compound on TLC and was isolated as a yellow solid; yield: 1.69 g (92%); m.p. = 138–139 °C; Rf = 0.5 (petroleum ether / ethyl acetate 4:3, *v*/*v*). IR (KBr): ν 3455 (N-H), 3344 (N-H), 2931 (C_{aliph}-H), 2890 (C_{aliph}-H), 2168 (CN), 1684 (C=O), 1560 (C=C), 1603, 1238 (C_{Ar}–N), 1079 (C–O), 811, 784 (C-H bending), 699 (C-H rocking), 533 (C-Se); ^{1}H NMR (400 MHz, DMSO- *d*$_6$) δ 7.70 (s, 2H, Ar-H), 7.44 (d, J = 8.6 Hz, 2H, Ar-H), 7.00 (s, 4H, 2NH_2), 6.77 (d, J = 8.6 Hz, 2H, Ar-H), 3.74 (s, 6H, 2OCH_3). ^{13}C NMR (101 MHz, DMSO-*d*$_6$) δ 167.00, 151.91, 140.57, 138.13, 117.63, 113.57, 108.98, 51.52. MS (EI, 70 ev) *m/z* (%) = 460.15 (M+H, 20.76), 459.15 (M, 5.20) or 230 (24.42), 119 (9.45), 91 (100.0, base peak), 65 (8.88).

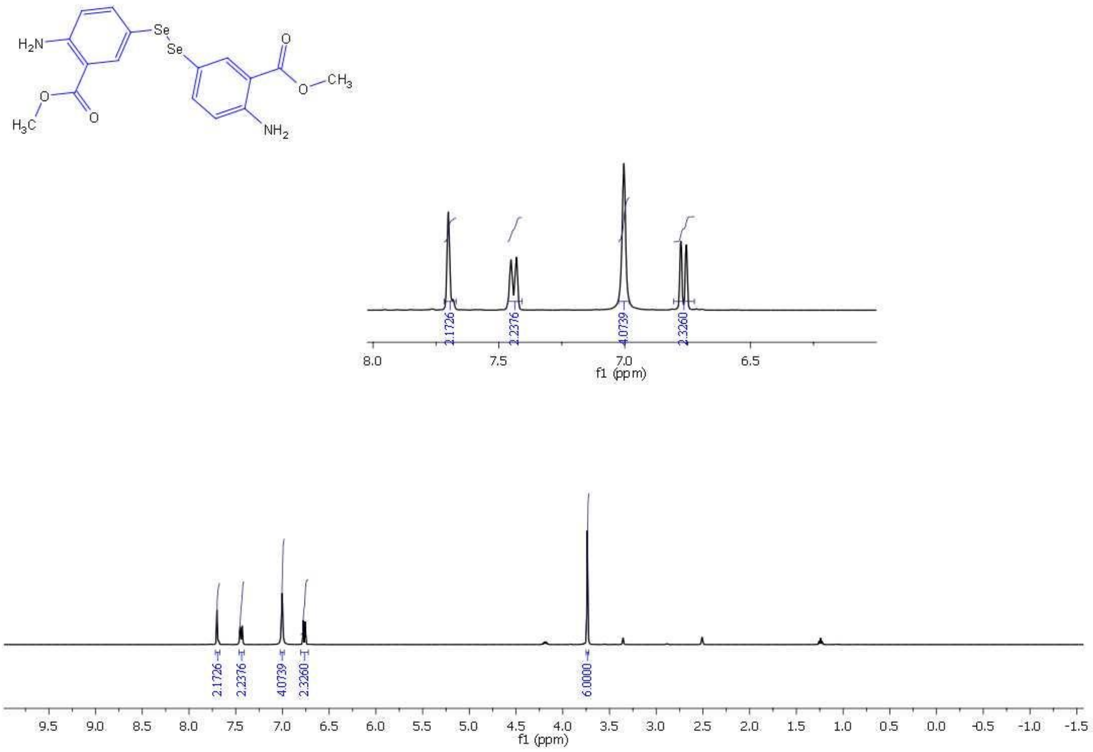

^{1}H NMR chart of compound **3**

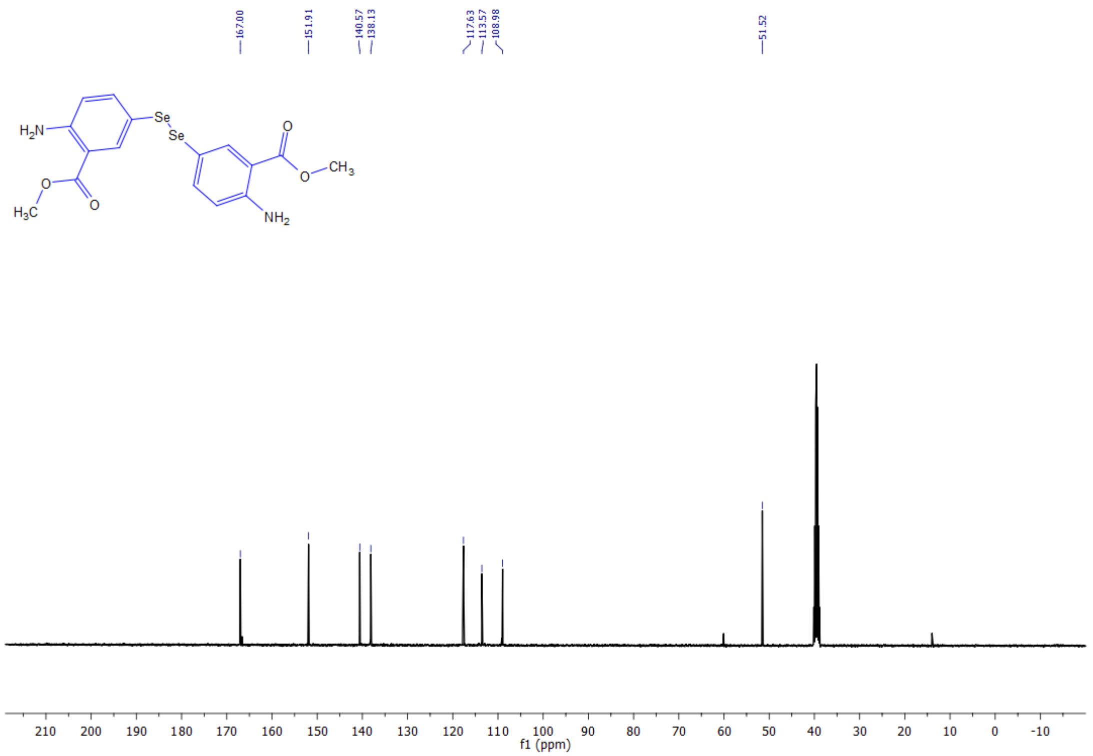

^{13}C NMR chart of compound **3**

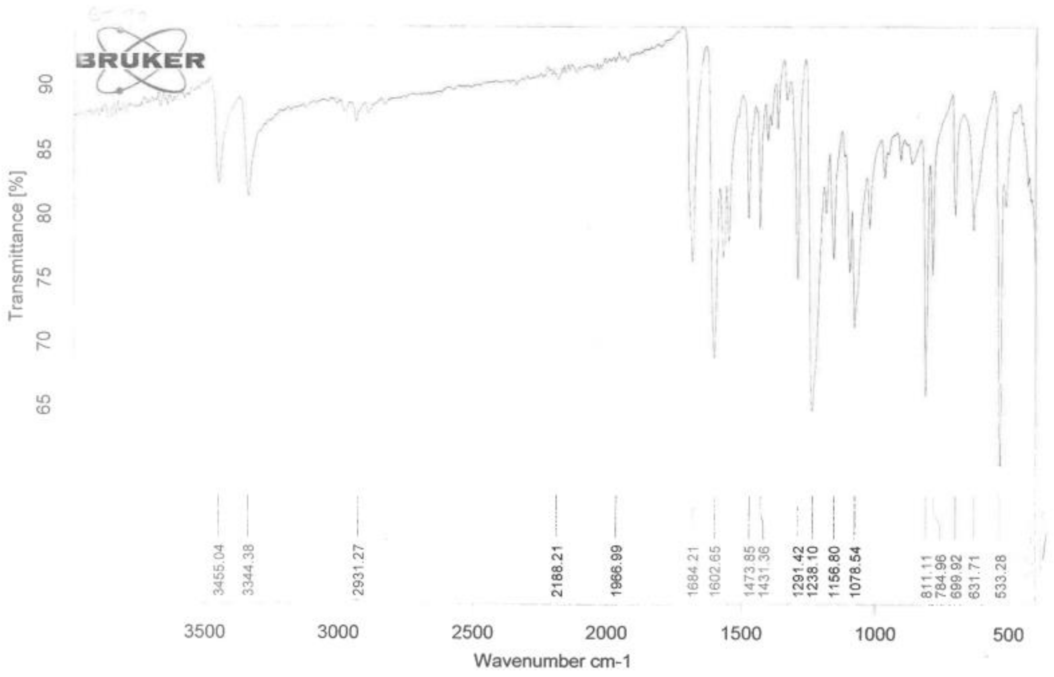

IR chart of compound **3**

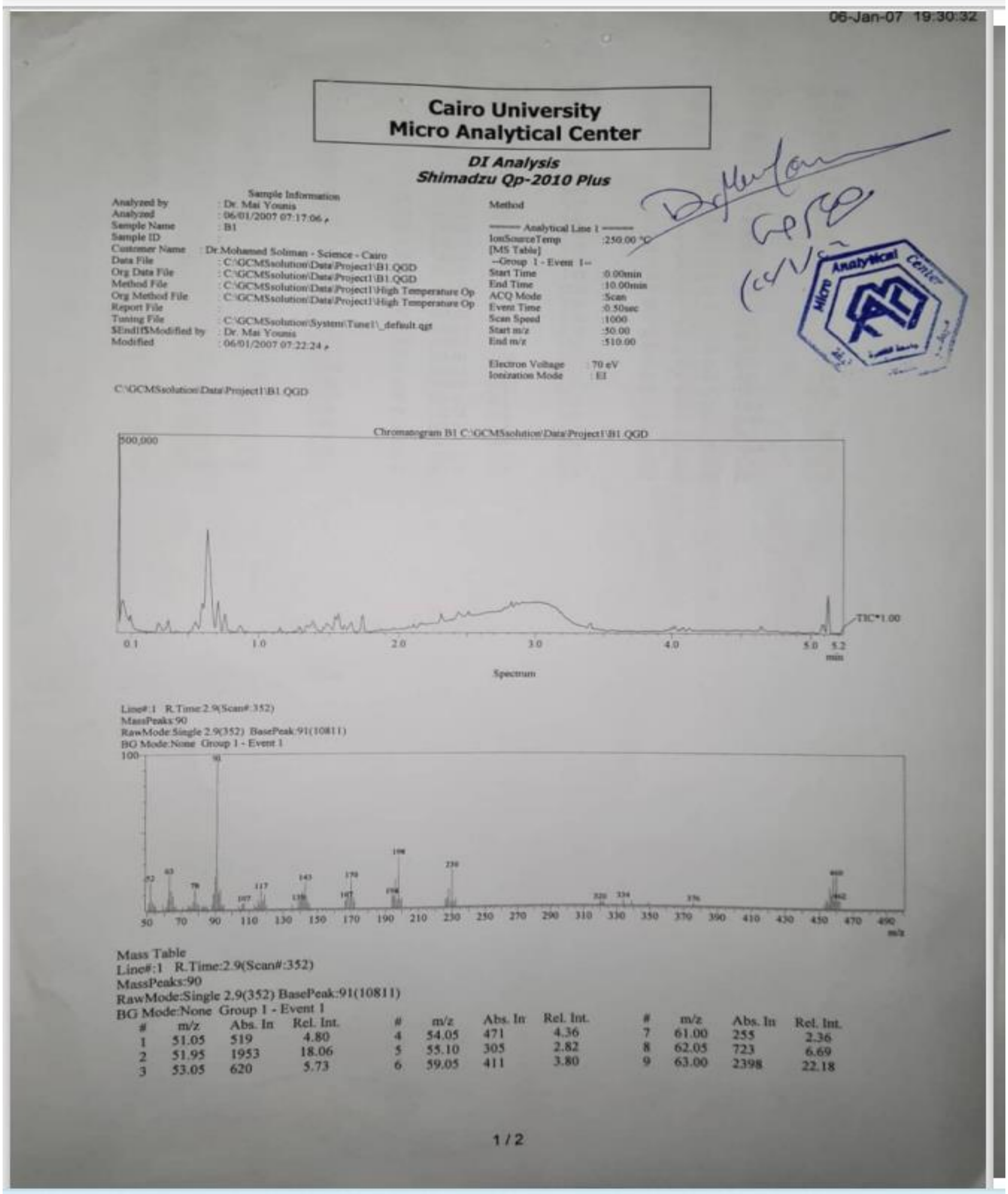
06-Jan-07 19:30:32

Cairo University
Micro Analytical Center

DI Analysis
Shimadzu Qp-2010 Plus

Sample Information

Analyzed by	: Dr. Mai Younis
Analyzed	: 06/01/2007 07:17:06 ⸗
Sample Name	: B1
Sample ID	:
Customer Name	: Dr.Mohamed Soliman - Science - Cairo
Data File	: C:\GCMSsolution\Data\Project1\B1.QGD
Org Data File	: C:\GCMSsolution\Data\Project1\B1.QGD
Method File	: C:\GCMSsolution\Data\Project1\High Temperature Op
Org Method File	: C:\GCMSsolution\Data\Project1\High Temperature Op
Report File	:
Tuning File	: C:\GCMSsolution\System\Tune1_default.qgt
$EndIf$Modified by	: Dr. Mai Younis
Modified	: 06/01/2007 07:22:24 ⸗

Method

——— Analytical Line 1 ———

IonSourceTemp	:250.00 °C
[MS Table]	
--Group 1 - Event 1--	
Start Time	:0.00min
End Time	:10.00min
ACQ Mode	:Scan
Event Time	:0.50sec
Scan Speed	:1000
Start m/z	:50.00
End m/z	:510.00
Electron Voltage	: 70 eV
Ionization Mode	: EI

C:\GCMSsolution\Data\Project1\B1.QGD

Mass Table
Line#:1 R.Time:2.9(Scan#:352)
MassPeaks:90
RawMode:Single 2.9(352) BasePeak:91(10811)
BG Mode:None Group 1 - Event 1

#	m/z	Abs. In	Rel. Int.	#	m/z	Abs. In	Rel. Int.	#	m/z	Abs. In	Rel. Int.
1	51.05	519	4.80	4	54.05	471	4.36	7	61.00	255	2.36
2	51.95	1953	18.06	5	55.10	305	2.82	8	62.05	723	6.69
3	53.05	620	5.73	6	59.05	411	3.80	9	63.00	2398	22.18

1 / 2

Mass chart of compound **3**

#	m/z	Abs. In	Rel. Int.
10	64.05	1311	12.13
11	65.05	960	8.88
12	66.00	278	2.57
13	67.00	247	2.28
14	71.00	202	1.87
15	74.00	313	2.90
16	75.00	255	2.36
17	76.00	214	1.98
18	77.05	571	5.28
19	78.05	1366	12.64
20	79.10	466	4.31
21	80.10	388	3.59
22	82.10	233	2.16
23	83.10	260	2.40
24	84.10	220	2.03
25	85.10	278	2.57
26	88.05	377	3.49
27	89.05	1081	10.00
28	90.05	2287	21.15
29	91.05	10811	100.00
30	92.05	1150	10.64
31	92.95	1429	13.22
32	94.00	254	2.35
33	95.00	354	3.27
34	104.00	249	2.30
35	106.10	398	3.68
36	107.00	444	4.11

#	m/z	Abs. In	Rel. Int.
37	113.00	308	2.85
38	113.90	222	2.05
39	114.90	636	5.88
40	116.05	385	3.56
41	117.00	1319	12.20
42	118.05	599	5.54
43	119.10	1022	9.45
44	120.10	310	2.87
45	135.10	289	2.67
46	139.00	482	4.46
47	140.00	614	5.68
48	141.05	1196	11.06
49	142.05	619	5.73
50	143.00	1894	17.52
51	144.00	850	7.86
52	144.95	409	3.78
53	166.05	493	4.56
54	167.05	639	5.91
55	168.05	1148	10.62
56	169.15	688	6.36
57	170.05	2031	18.79
58	171.10	802	7.42
59	172.10	420	3.88
60	194.05	863	7.98
61	195.05	858	7.94
62	196.00	1993	18.43
63	197.05	575	5.32

#	m/z	Abs. In	Rel. Int.
64	198.00	3602	33.32
65	199.00	591	5.47
66	200.05	690	6.38
67	226.05	505	4.67
68	227.05	655	6.06
69	228.00	1222	11.30
70	229.15	316	2.92
71	230.05	2640	24.42
72	231.05	347	3.21
73	232.05	594	5.49
74	320.00	290	2.68
75	321.00	222	2.05
76	322.00	228	2.11
77	334.00	398	3.68
78	339.00	318	2.94
79	349.00	230	2.13
80	376.00	215	1.99
81	453.00	263	2.43
82	454.20	604	5.59
83	455.20	425	3.93
84	456.25	1479	13.68
85	457.20	1015	9.39
86	458.20	2169	20.06
87	459.15	562	5.20
88	460.15	2244	20.76
89	461.15	521	4.82
90	462.10	618	5.72

Mass chart of compound **3**

Synthesis of methyl 2-amino-5-(methylselanyl) benzoate (4) [1, 4]

Compound **4** was synthesized from dimethyl 5,5'-diselanediylbis (2-aminobenzoate) (**3**) (2 mmol, 916 mg) and methyl iodide (4.4 mmol, 0.27 mL). It was isolated as brown oil; yield: 402 mg (82%); Rf = 0.6 (petroleum ether / ethyl acetate 4:2, *v/v*). IR (KBr): ν 3474 (N-H), 3364 (N-H), 2948 (C_{aliph}-H), 2926 (C_{aliph}-H), 1686 (C=O), 1607, 1292 (C_{Ar}–N), 1110 (C–O), 815, 789, 696 (C-H bending), 559 (C-Se); ^{1}H NMR (400 MHz, DMSO- d_6) δ 7.82 (s, 1H, Ar-H), 7.40 (d, J = 8.6 Hz, 1H, Ar-H), 6.77 (d, J = 8.6 Hz, 1H, Ar-H), 6.75 (s, 2H, NH_2), 3.62 (s, 3H, OCH_3), 2.22 (s, 3H, CH_3). ^{13}C NMR (101 MHz, DMSO- d_6) δ 167.70, 151.00, 138.90, 135.03, 118.21, 113.98, 109.87, 51.98, 9.17. MS (EI, 70 ev) *m/z* (%) = 245.10 (M, 100.0, base peak), 230 (38.06),186 (10.46), 170 (35.08), 91 (91.10).

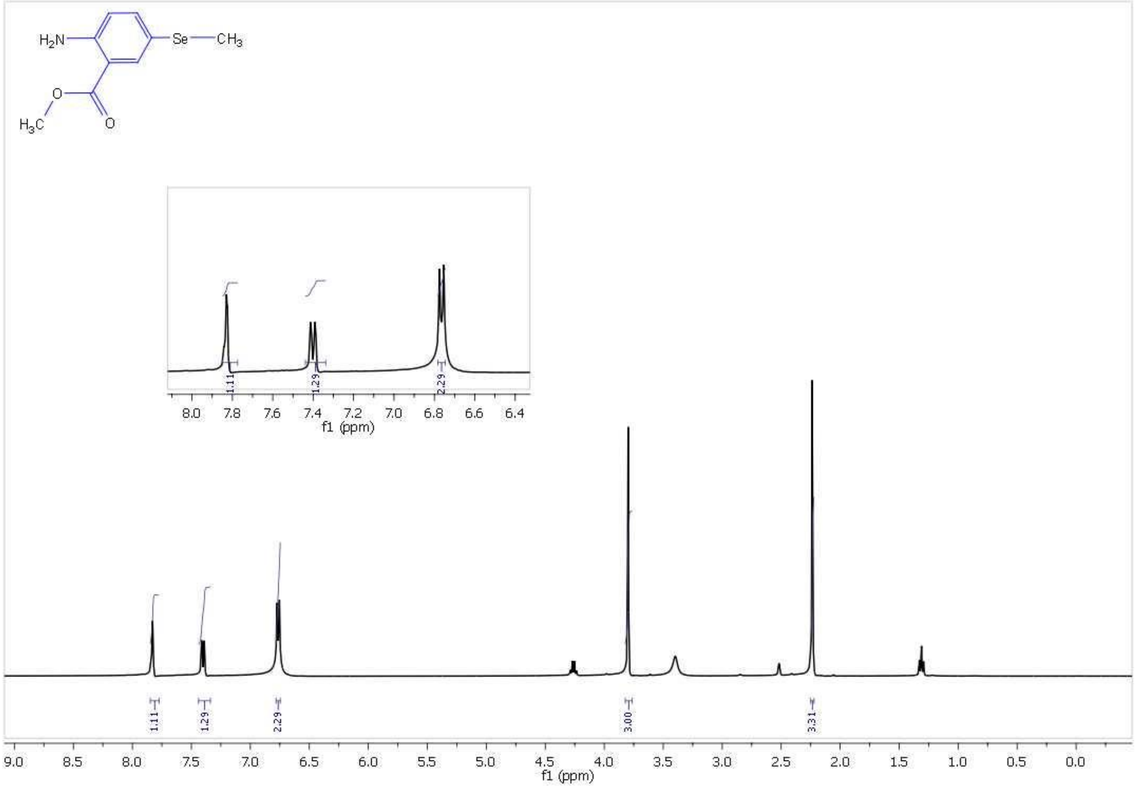

^{1}H NMR chart of compound **4**

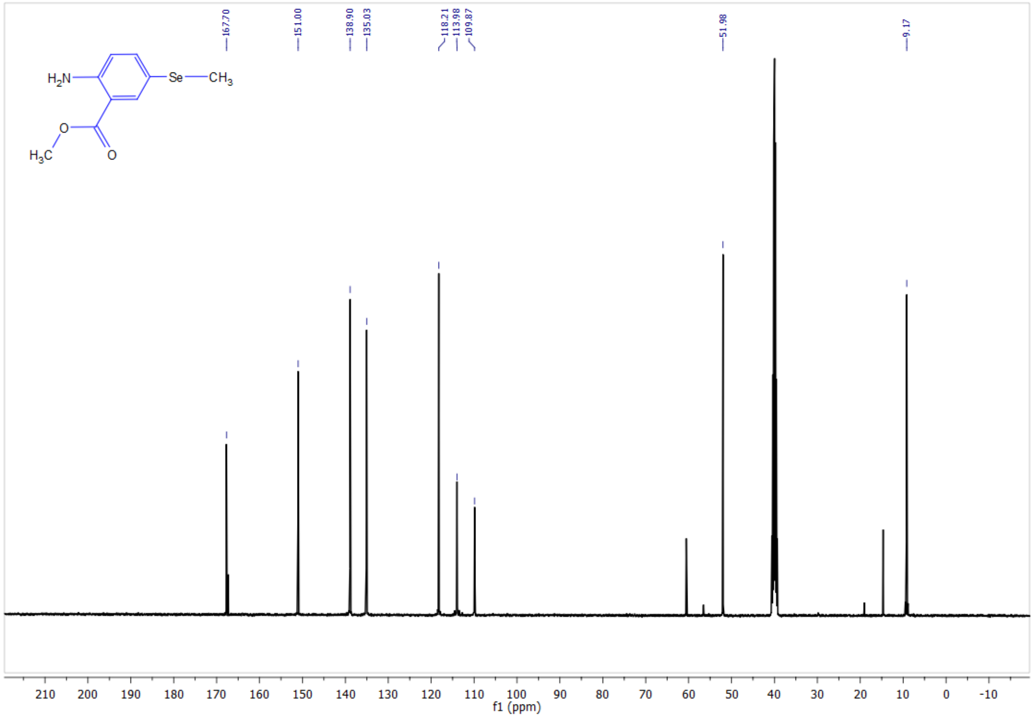

^{13}C NMR chart of compound **4**

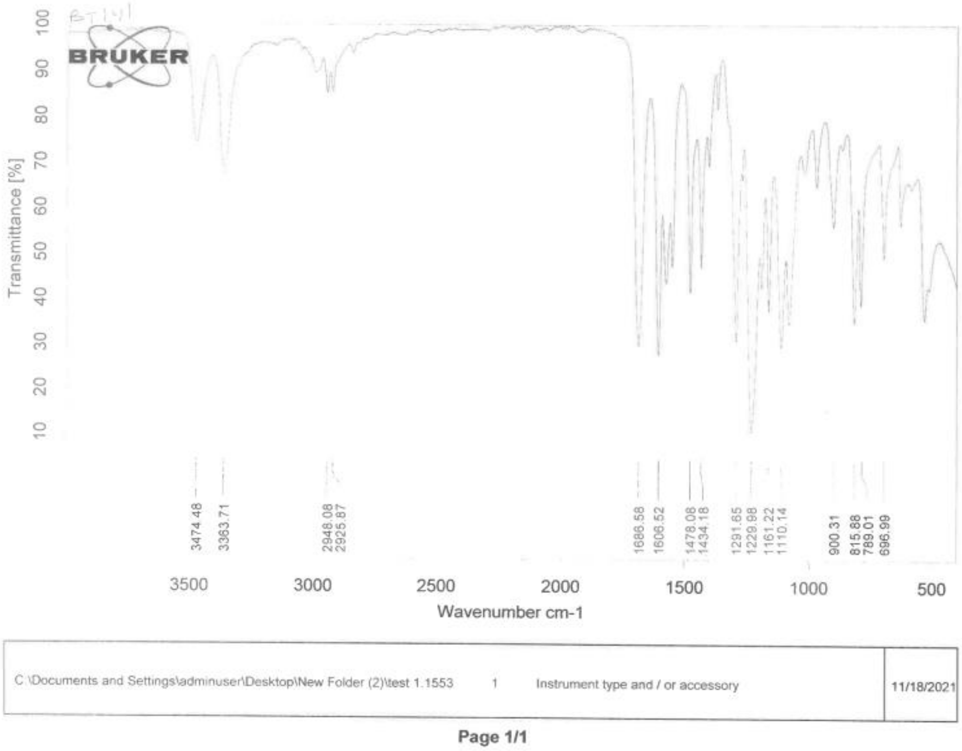

IR chart of compound **4**

Cairo University
Micro Analytical Center

DI Analysis
Shimadzu Qp-2010 Plus

Sample Information

Analyzed by	: Dr. Mai Younis
Analyzed	: 16/01/2007 03:02:11 ص
Sample Name	: B35
Sample ID	:
Customer Name	: Dr.Mohamed Soliman - Science - Cairo
Data File	: C:\GCMSsolution\Data\Project1\B35.QGD
Org Data File	: C:\GCMSsolution\Data\Project1\B35.QGD
Method File	: C:\GCMSsolution\Data\Project1\High Temperature Op
Org Method File	: C:\GCMSsolution\Data\Project1\High Temperature Op
Report File	:
Tuning File	: C:\GCMSsolution\System\Tune1_default.qgt
EndModified by	: Dr. Mai Younis
Modified	: 16/01/2007 03:12:11 ص

Method

——— Analytical Line 1 ———

IonSourceTemp	:250.00 °C
[MS Table]	
--Group 1 - Event 1--	
Start Time	:0.00min
End Time	:10.00min
ACQ Mode	:Scan
Event Time	:0.50sec
Scan Speed	:1000
Start m/z	:50.00
End m/z	:510.00
Electron Voltage	: 70 eV
Ionization Mode	: EI

C:\GCMSsolution\Data\Project1\B35.QGD

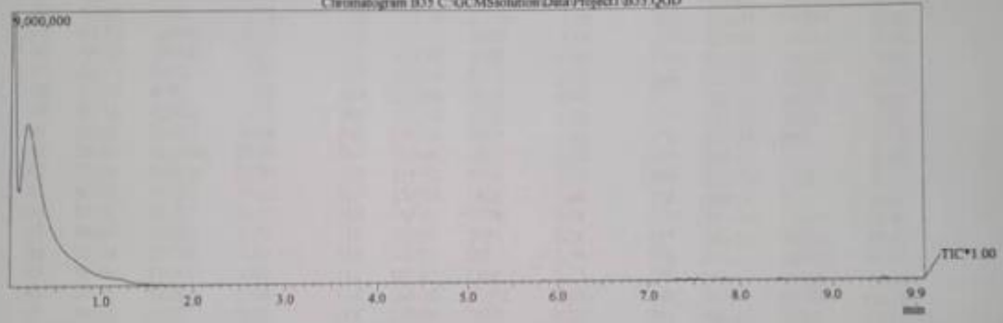

Spectrum

Line#:1 R.Time:0.2(Scan#:26)
MassPeaks:205
RawMode:Single 0.2(26) BasePeak:245(391154)
BG Mode:None Group 1 - Event 1

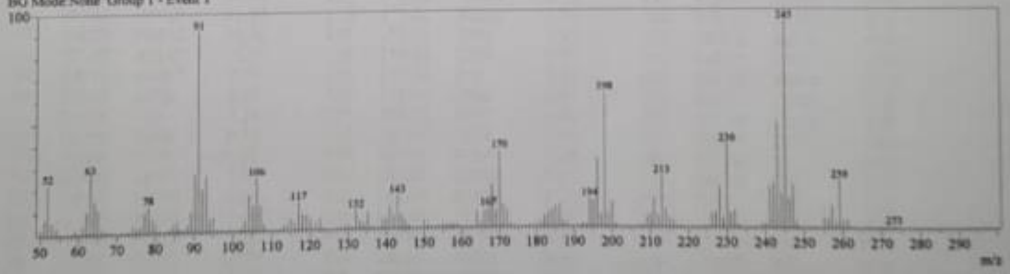

Mass Table
Line#:1 R.Time:0.2(Scan#:26)
MassPeaks:205
RawMode:Single 0.2(26) BasePeak:245(391154)
BG Mode:None Group 1 - Event 1

#	m/z	Abs. In	Rel. Int.	#	m/z	Abs. In	Rel. Int.	#	m/z	Abs. In	Rel. Int.
1	50.05	10411	2.66	4	53.00	21482	5.49	7	56.05	488	0.12
2	51.05	26260	6.71	5	54.05	11184	2.86	8	57.05	709	0.18
3	52.00	89298	22.83	6	55.00	2246	0.57	9	58.05	1164	0.30

1 / 2

Mass chart of compound **4**

16-Jan-07 03:18:00

#	m/z	Abs. In	Rel. Int.	#	m/z	Abs. In	Rel. Int.	#	m/z	Abs. In	Rel. Int.
10	59.00	6150	1.57	76	126.05	2179	0.56	142	195.00	47017	12.02
11	60.05	1575	0.40	77	127.05	3204	0.82	143	196.00	122146	31.23
12	61.05	14415	3.69	78	128.00	4023	1.03	144	197.05	22307	5.70
13	62.05	39733	10.16	79	129.05	5617	1.44	145	198.00	241660	61.78
14	63.05	103836	26.55	80	130.00	7762	1.98	146	198.95	25629	6.55
15	64.05	57318	14.65	81	131.15	4478	1.14	147	200.00	43719	11.18
16	65.05	43819	11.20	82	132.10	33902	8.67	148	200.95	4204	1.07
17	66.10	9838	2.52	83	133.10	15628	4.00	149	201.95	533	0.14
18	67.05	4572	1.17	84	134.15	8746	2.24	150	207.00	2228	0.57
19	68.05	2933	0.75	85	135.10	31116	7.95	151	208.05	2526	0.65
20	69.10	1041	0.27	86	136.15	5476	1.40	152	209.05	20084	5.13
21	70.05	837	0.21	87	137.00	2783	0.71	153	210.05	23473	6.00
22	71.05	1646	0.42	88	138.05	3352	0.86	154	211.00	50268	12.85
23	72.05	1669	0.43	89	139.00	15473	3.96	155	212.05	22070	5.64
24	72.95	2952	0.75	90	140.00	17660	4.51	156	213.00	91327	23.35
25	74.05	12141	3.10	91	141.00	39022	9.98	157	214.00	31220	7.98
26	75.10	10198	2.61	92	142.05	21311	5.45	158	215.00	18164	4.64
27	76.10	12385	3.17	93	143.00	58711	15.01	159	216.00	12508	3.20
28	77.10	36498	9.33	94	144.00	25175	6.44	160	216.95	1191	0.30
29	78.05	47310	12.09	95	145.00	13801	3.53	161	218.00	1618	0.41
30	79.10	24441	6.25	96	146.00	5042	1.29	162	224.00	2599	0.66
31	80.00	15313	3.91	97	147.00	1039	0.27	163	225.05	1510	0.39
32	81.00	4794	1.23	98	148.10	804	0.21	164	226.05	26426	6.76
33	82.55	1873	0.48	99	149.15	1515	0.39	165	227.05	28172	7.20
34	83.55	6981	1.78	100	150.10	13154	3.36	166	228.05	72774	18.60
35	84.55	13095	3.35	101	151.10	4439	1.13	167	229.05	16399	4.19
36	85.50	19868	5.08	102	151.95	1797	0.46	168	230.05	148867	38.06
37	86.10	5970	1.53	103	153.00	3161	0.81	169	231.00	25292	6.47
38	87.05	4792	1.23	104	154.00	3514	0.90	170	232.05	29923	7.65
39	88.10	12948	3.31	105	155.00	5122	1.31	171	233.00	4730	1.21
40	89.10	37161	9.50	106	156.00	4514	1.15	172	233.95	605	0.15
41	90.15	101589	25.97	107	157.05	6492	1.66	173	238.15	734	0.19
42	91.10	358905	91.76	108	158.00	3975	1.02	174	239.10	6298	1.61
43	92.05	75198	19.22	109	159.00	5625	1.44	175	240.15	7887	2.02
44	93.00	97377	24.89	110	160.00	1470	0.38	176	241.10	72847	18.62
45	94.00	22561	5.77	111	161.00	1084	0.28	177	242.10	80234	20.51
46	95.00	26670	6.82	112	162.00	380	0.10	178	243.10	187193	47.86
47	96.00	4114	1.05	113	163.15	1307	0.33	179	244.15	60066	15.36
48	97.05	4225	1.08	114	164.10	28951	7.40	180	245.10	391154	100.00
49	98.35	2449	0.63	115	165.05	9689	2.48	181	246.05	51306	13.12
50	99.30	3386	0.87	116	166.00	30183	7.72	182	247.05	74480	19.04
51	100.25	1374	0.35	117	167.05	33926	8.67	183	248.05	8926	2.28
52	101.05	1757	0.45	118	168.00	75583	19.32	184	249.05	953	0.24
53	102.10	5450	1.39	119	169.05	30276	7.74	185	253.10	1460	0.37
54	103.15	15671	4.01	120	170.00	137220	35.08	186	254.15	1011	0.26
55	104.15	62814	16.06	121	171.00	40180	10.27	187	255.10	15329	3.92
56	105.25	45718	11.69	122	172.00	30348	7.76	188	256.15	16105	4.12
57	106.20	94842	24.25	123	173.00	7290	1.86	189	257.10	40538	10.36
58	107.15	45188	11.55	124	174.00	1239	0.32	190	258.15	9549	2.44
59	108.15	10687	2.73	125	177.20	238	0.06	191	259.10	84319	21.56
60	109.05	2089	0.53	126	178.15	3162	0.81	192	260.05	12047	3.08
61	110.05	710	0.18	127	179.05	1817	0.46	193	261.10	15478	3.96
62	111.05	1314	0.34	128	180.00	3514	0.90	194	262.05	2350	0.60
63	112.05	1199	0.31	129	181.05	9794	2.50	195	263.05	335	0.09
64	113.00	7610	1.95	130	182.00	19970	5.11	196	264.10	220	0.06
65	114.00	10354	2.65	131	183.00	25158	6.43	197	269.15	730	0.19
66	115.00	21430	5.48	132	184.00	31201	7.98	198	270.15	629	0.16
67	116.05	12818	3.28	133	185.00	38050	9.73	199	271.15	1493	0.38
68	117.05	50031	12.79	134	186.00	40924	10.46	200	272.25	533	0.14
69	118.05	27671	7.07	135	187.00	10028	2.56	201	273.15	2823	0.72
70	119.10	26692	6.82	136	188.00	6714	1.72	202	274.20	558	0.14
71	120.10	18391	4.70	137	189.00	868	0.22	203	275.20	654	0.17
72	121.55	12099	3.09	138	191.05	296	0.08	204	276.20	290	0.07
73	122.60	19750	5.05	139	192.00	4826	1.23	205	277.10	369	0.09
74	123.55	4338	1.11	140	193.05	2875	0.74				
75	125.05	1061	0.27	141	194.00	49812	12.73				

Mass chart of compound **4**

Synthesis of methyl 2-amino-5-(benzylselanyl) benzoate (5)[1, 4]

Compound **5** was synthesized from dimethyl 5,5'-diselanediylbis(2-aminobenzoate) (**3**) (2 mmol, 916 mg) and benzyl chloride (4.4 mmol, 0.50 mL). It was isolated as light brown solid; yield: 597 mg (93%); m.p. = 78 ^{o}C; Rf = 0.6 (petroleum ether / ethyl acetate 4:3, *v*/*v*). IR (KBr): ν 3462 (N-H), 3353 (N-H), 2977 (C_{aliph}-H), 2946 (C_{aliph}-H), 1672 (C=O), 1621, 1436, 1234 (C_{Ar}-N), 1114 (C-O), 810 (C-H bending), 692 (C-H rocking), 595 (C-Se); ^{1}H NMR (400 MHz, DMSO- d_6) δ 7.70 (s, 1H, Ar-H), 7.30 (d, J = 8.6 Hz, 1H, Ar-H), 7.23 (dd, J = 8.6 Hz, J = 2.1 Hz, 2H, Ar-H), 7.19 (d, J = 8.7 Hz, 2H, Ar-H), 7.14 (d, J = 8.6 Hz, 2H, Ar-H), 6.82 (s, 2H, NH_2), 4.00 (s, 2H, $SeCH_2$), 3.77 (s, 3H, OCH_3); ^{13}C NMR (101 MHz, DMSO- d_6) δ 167.69, 151.53, 141.06, 139.66, 137.95, 129.24, 128.65, 126.98, 117.87, 112.42, 109.68, 51.94, 32.75. MS (EI, 70 ev) *m/z* (%) = 321.20 (M, 46.87), 244.10 (2.77), 230 (34.88), 150.15 (1.02), 91.05 (100.0, base peak).

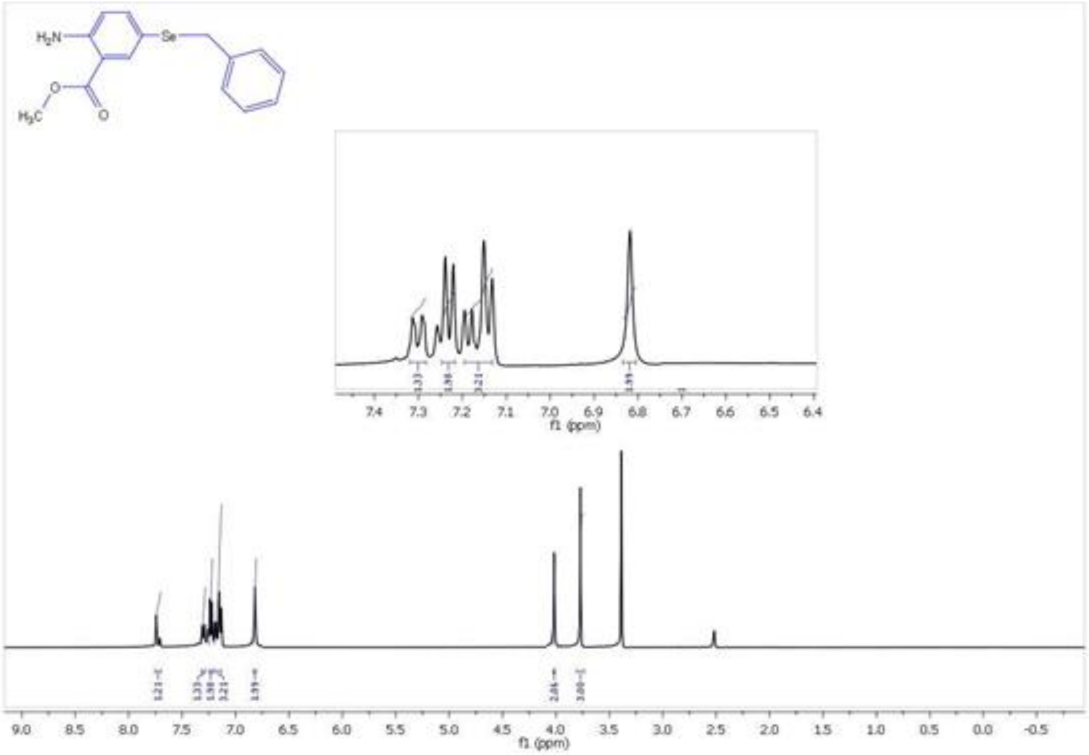

^{1}H NMR chart of compound **5**

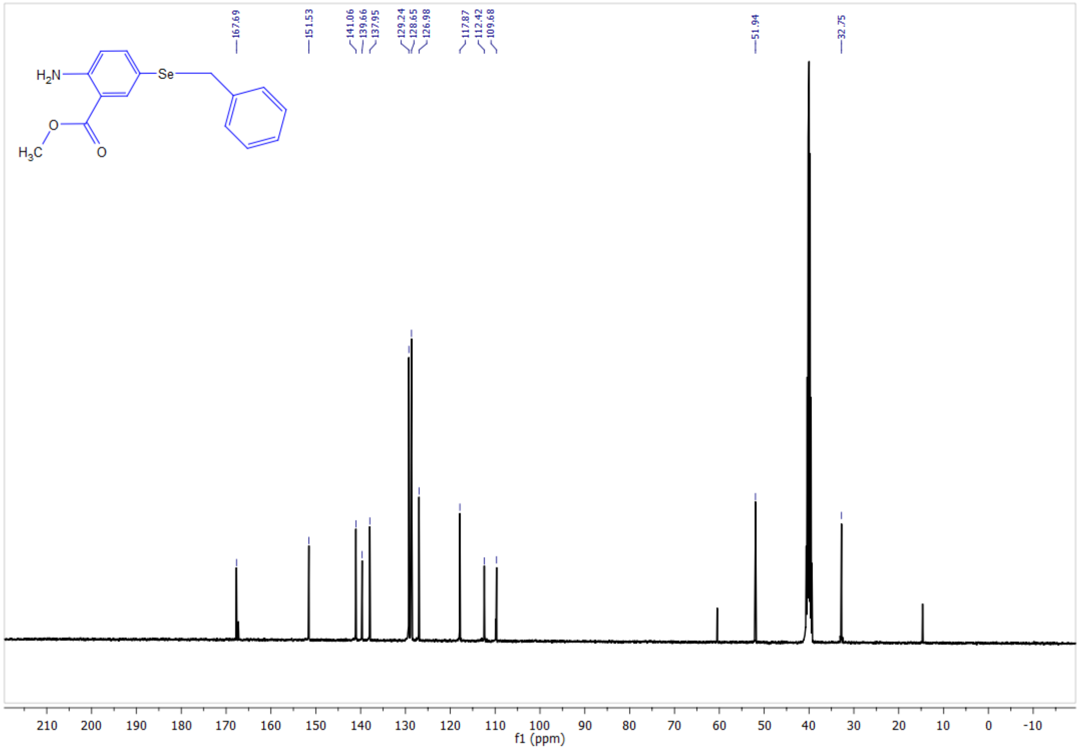

^{13}C NMR chart of compound **5**

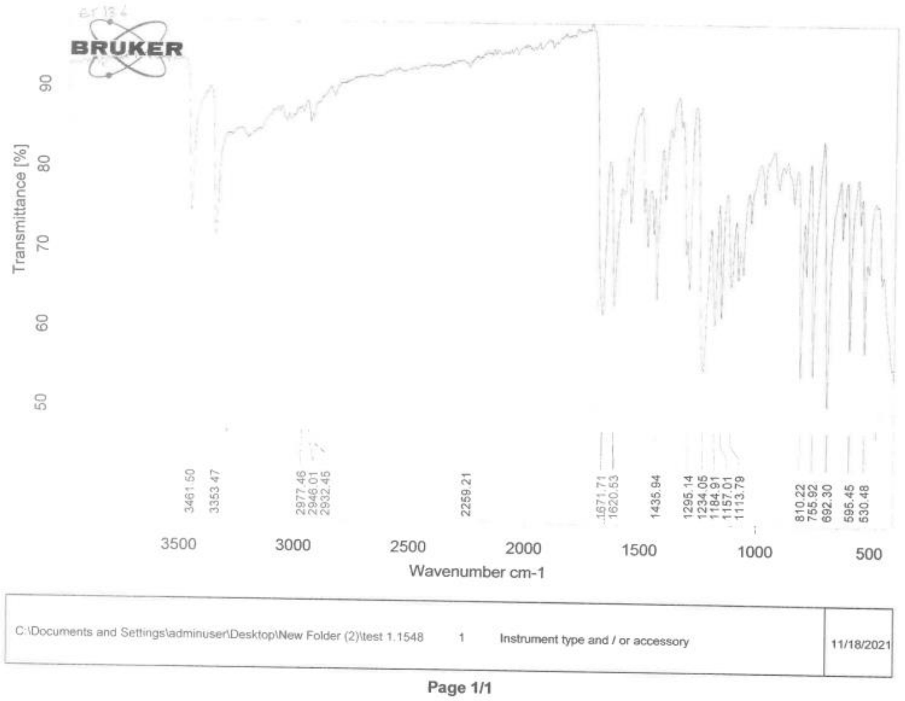

IR chart of compound **5**

06-Jan-07 19:54:17

Cairo University
Micro Analytical Center

DI Analysis
Shimadzu Qp-2010 Plus

Sample Information

Analyzed by	: Dr. Mai Younis
Analyzed	: 06/01/2007 07:49:46 ،
Sample Name	: B22
Sample ID	:
Customer Name	: Dr.Mohamed Soliman - Science - Cairo
Data File	: C:\GCMSsolution\Data\Project1\B22.QGD
Org Data File	: C:\GCMSsolution\Data\Project1\B22.QGD
Method File	: C:\GCMSsolution\Data\Project1\High Temperature Op
Org Method File	: C:\GCMSsolution\Data\Project1\High Temperature Op
Report File	:
Tuning File	: C:\GCMSsolution\System\Tune1_default.qgt
$EndIf$Modified by	: Dr. Mai Younis
Modified	: 06/01/2007 07:51:49 ،

Method

——— Analytical Line 1 ———

IonSourceTemp	:250.00 °C
[MS Table]	
--Group 1 - Event 1--	
Start Time	:0.00min
End Time	:10.00min
ACQ Mode	:Scan
Event Time	:0.50sec
Scan Speed	:1000
Start m/z	:50.00
End m/z	:510.00
Electron Voltage	:70 eV
Ionization Mode	:EI

C:\GCMSsolution\Data\Project1\B22.QGD

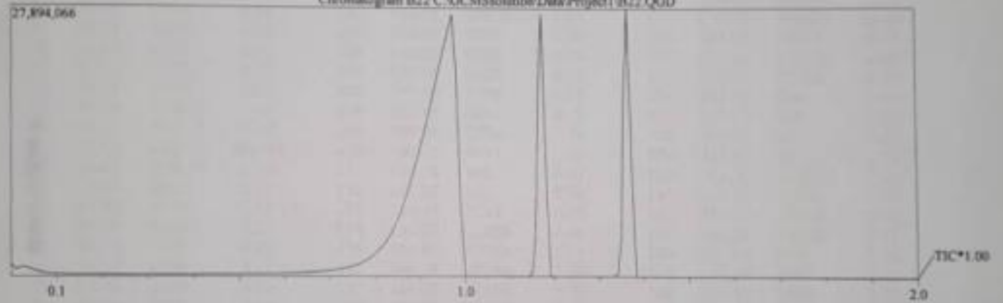

Spectrum

Line#:1 R.Time:1.2(Scan#:143)
MassPeaks:244(Peak Elimination m/z: 423.20, 425.30)
RawMode:Single 1.2(143) BasePeak:91(2025185)
BG Mode:None Group 1 - Event 1

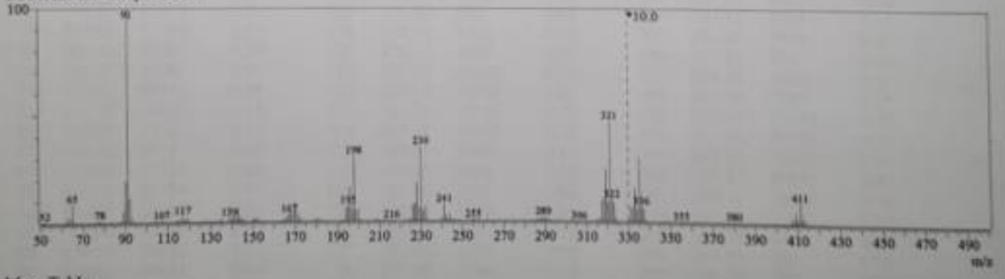

Mass Table
Line#:1 R.Time:1.2(Scan#:143)
MassPeaks:244(Peak Elimination m/z: 423.20, 425.30)
RawMode:Single 1.2(143) BasePeak:91(2025185)
BG Mode:None Group 1 - Event 1

#	m/z	Abs. In	Rel. Int.	#	m/z	Abs. In	Rel. Int.	#	m/z	Abs. In	Rel. Int.
1	50.05	5073	0.25	4	52.95	3146	0.16	7	57.00	1097	0.05
2	51.00	11465	0.57	5	54.00	1751	0.09	8	58.05	483	0.02
3	52.00	13521	0.67	6	55.00	583	0.03	9	59.00	2993	0.15

1 / 3

Mass chart of compound **5**

#	m/z	Abs. In	Rel. Int.
10	60.05	391	0.02
11	61.05	3772	0.19
12	62.05	17107	0.84
13	63.00	56926	2.81
14	64.05	36812	1.82
15	65.00	176463	8.71
16	65.95	12878	0.64
17	67.00	1580	0.08
18	68.05	483	0.02
19	69.10	985	0.05
20	70.10	1497	0.07
21	71.10	1663	0.08
22	72.05	379	0.02
23	73.05	2080	0.10
24	74.05	4921	0.24
25	75.05	5212	0.26
26	76.05	6114	0.30
27	77.05	10886	0.54
28	78.05	20811	1.03
29	79.05	8101	0.40
30	80.00	5090	0.25
31	81.05	1605	0.08
32	82.15	1045	0.05
33	83.10	2590	0.13
34	84.15	2599	0.13
35	85.15	4470	0.22
36	86.05	4174	0.21
37	87.15	6312	0.31
38	88.15	20787	1.03
39	89.15	110480	5.46
40	90.15	391518	19.33
41	91.05	202518	100.00
42	92.05	228419	11.28
43	93.05	46032	2.27
44	94.00	4180	0.21
45	95.00	8522	0.42
46	96.15	1388	0.07
47	97.10	2596	0.13
48	98.10	2490	0.12
49	99.10	2721	0.13
50	100.15	911	0.04
51	101.10	2750	0.14
52	102.10	4402	0.22
53	103.10	7242	0.36
54	104.05	11796	0.58
55	105.05	7012	0.35
56	106.10	11636	0.57
57	107.05	13258	0.65
58	108.00	3873	0.19
59	109.15	1378	0.07
60	110.05	979	0.05
61	111.05	2440	0.12
62	112.05	2793	0.14
63	113.05	9979	0.49
64	114.05	12822	0.63
65	115.00	27724	1.37
66	116.05	18052	0.89
67	117.00	53988	2.67
68	118.05	26835	1.33
69	119.05	43451	2.15
70	120.05	15785	0.78
71	121.10	4878	0.24
72	122.05	2063	0.10
73	123.10	1527	0.08
74	124.05	682	0.03
75	125.05	4002	0.20
76	126.05	5459	0.27
77	127.05	10480	0.52
78	128.05	10844	0.54
79	129.05	9313	0.46
80	130.05	11086	0.55
81	131.10	5054	0.25
82	132.05	10987	0.54
83	133.10	3684	0.18
84	134.15	9264	0.46
85	135.05	30121	1.49
86	136.05	4109	0.20
87	137.05	5343	0.26
88	138.05	7879	0.39
89	139.00	35607	1.76
90	140.05	41787	2.06
91	141.00	77244	3.81
92	142.05	47101	2.33
93	142.95	126529	6.25
94	143.95	48735	2.41
95	144.95	28945	1.43
96	146.05	23129	1.14
97	146.95	4067	0.20
98	148.15	1843	0.09
99	149.15	8597	0.42
100	150.15	20578	1.02
101	151.05	33271	1.64
102	152.05	32505	1.61
103	153.05	20872	1.03
104	154.05	7855	0.39
105	155.00	4855	0.24
106	155.95	3585	0.18
107	157.00	3398	0.17
108	157.95	2693	0.13
109	159.05	2274	0.11
110	160.05	4165	0.21
111	161.00	602	0.03
112	162.05	482	0.02
113	163.05	3714	0.18
114	164.05	12889	0.64
115	165.05	22436	1.11
116	166.05	47284	2.33
117	167.05	66556	3.29
118	168.00	102111	5.04
119	169.05	77098	3.81
120	169.95	174770	8.63
121	170.95	74461	3.68
122	172.00	39018	1.93
123	172.95	11808	0.58
124	173.95	1305	0.06
125	176.15	889	0.04
126	177.15	2401	0.12
127	178.15	5969	0.29
128	179.15	10187	0.50
129	180.15	36701	1.81
130	181.10	18865	0.93
131	182.15	14965	0.74
132	183.05	3441	0.17
133	184.00	2350	0.12
134	185.10	1281	0.06
135	186.05	1167	0.06
136	190.05	602	0.03
137	191.05	1736	0.09
138	192.05	12467	0.62
139	193.05	17901	0.88
140	194.05	131489	6.49
141	195.05	146464	7.23
142	196.00	323593	15.98
143	197.05	113333	5.60
144	198.00	620129	30.62
145	198.95	99120	4.89
146	200.00	113441	5.60
147	200.95	16159	0.80
148	201.95	1028	0.05
149	204.25	5464	0.27
150	205.15	701	0.03
151	206.20	1020	0.05
152	207.25	2877	0.14
153	208.20	14284	0.71
154	209.15	17645	0.87
155	210.05	5896	0.29
156	211.15	1898	0.09
157	212.05	5471	0.27
158	213.10	2154	0.11
159	214.05	4336	0.21
160	215.15	994	0.05
161	216.10	6855	0.34
162	217.05	395	0.02
163	218.10	921	0.05
164	221.20	846	0.04
165	223.15	1595	0.08
166	224.10	14468	0.71
167	225.15	19403	0.96
168	226.10	153471	7.58
169	227.15	166163	8.20
170	228.10	368934	18.22
171	229.15	120571	5.95
172	230.10	706377	34.88
173	231.05	94541	4.67
174	232.10	132870	6.56
175	233.05	15116	0.75
176	234.05	960	0.05
177	238.15	969	0.05
178	239.25	4131	0.20
179	240.25	35113	1.73
180	241.25	155831	7.69
181	242.10	54386	2.69
182	243.15	12588	0.62
183	244.10	56178	2.77
184	245.05	8044	0.40
185	246.10	9770	0.48
186	247.10	1503	0.07
187	254.35	1823	0.09
188	255.25	10721	0.53
189	256.15	3888	0.19
190	257.15	1891	0.09
191	258.15	4289	0.21
192	259.15	3074	0.15
193	260.15	2910	0.14
194	261.15	2231	0.11
195	262.20	1594	0.08
196	283.25	376	0.02
197	284.25	1803	0.09
198	285.20	7185	0.35
199	286.20	14742	0.73
200	287.15	23239	1.15
201	288.20	24534	1.21
202	289.15	36611	1.81
203	290.15	36028	1.78
204	291.10	11612	0.57
205	292.15	6462	0.32
206	293.15	552	0.03
207	306.20	302	0.01
208	308.10	636	0.03
209	310.05	707	0.03
210	314.25	1728	0.09
211	315.20	18481	0.91
212	316.25	23945	1.18
213	317.20	196281	9.69
214	318.25	227987	11.26
215	319.20	494574	24.42
216	320.25	188395	9.30

2 / 3

Mass chart of compound **5**

06-Jan-07 19:54:17

#	m/z	Abs. In	Rel. Int.
217	321.20	949119	46.87
218	322.15	206457	10.19
219	323.15	182324	9.00
220	324.05	37472	1.85
221	325.05	4024	0.20
222	329.25	824	0.04
223	330.25	1720	0.08
224	331.20	13207	0.65
225	332.25	14297	0.71
226	333.20	31734	1.57

#	m/z	Abs. In	Rel. Int.
227	334.25	12617	0.62
228	335.20	61590	3.04
229	336.15	14251	0.70
230	337.20	12284	0.61
231	338.15	2137	0.11
232	347.20	470	0.02
233	349.20	1524	0.08
234	355.20	474	0.02
235	378.20	226	0.01
236	380.20	604	0.03

#	m/z	Abs. In	Rel. Int.
237	407.30	3812	0.19
238	408.35	4545	0.22
239	409.25	10425	0.51
240	410.35	4385	0.22
241	411.25	19850	0.98
242	412.25	5834	0.29
243	413.25	4131	0.20
244	414.15	786	0.04

3 / 3

Mass chart of compound **5**

Synthesis of methyl 2-amino-5-((2-oxo-2-(phenylamino) ethyl) selanyl) benzoate (6)[1, 4]

Compound **6** was synthesized from dimethyl 5,5'-diselanediylbis (2-aminobenzoate) (**3**) (2.0 mmol, 916 mg) and 2-chloro-*N*-phenylacetamide (4.4 mmol, 744 mg). It was isolated as violet solid; yield: 699 mg (96%); m.p. = 126–128 °C; Rf = 0.50 (petroleum ether / ethyl acetate 4:2, *v*/*v*). IR (KBr): ν 3466 (N-H), 3354 (N-H), 3246 (N-H), 2945 (C_{aliph}-H), 1678, 1594 (C=O), 1542 (C=C), 1439, 1238 (C_{Ar}-N), 1090 (C-O), 752 (C-H bending), 695, 552 (C-Se), 539 (C-H rocking); ^{1}H NMR (400 MHz, DMSO- d_6) δ 9.99 (s, 1H, NH), 7.96 (s, 1H, Ar-H), 7.53 (d, J = 8.6 Hz, 2H, ArH), 7.48 (d, J = 8.6 Hz, 1H, ArH), 7.30 (dd, J = 8.6 Hz, J = 2.1 Hz, 2H, Ar-H), 7.04 (t, 1H, J = 8.6 Hz, Ar-H), 6.86 (s, 2H, NH_2), 6.75 (d, J = 8.6 Hz, 1H, Ar-H), 3.74 (s, 3H, OCH_3), 3.42 (s, 2H, $SeCH_2$); ^{13}C NMR (101 MHz, DMSO- d_6) δ 168.65, 167.65, 151.81, 141.13, 139.49, 138.20, 129.14, 123.74, 119.62, 117.89, 111, 109.71, 51.93, 32.33. MS (EI, 70 ev) *m/z* (%) = 364.20 (M, 100.0, base peak), 230 (40.48), 91.10 (31), 77.05 (24.13), 59 (14.56).

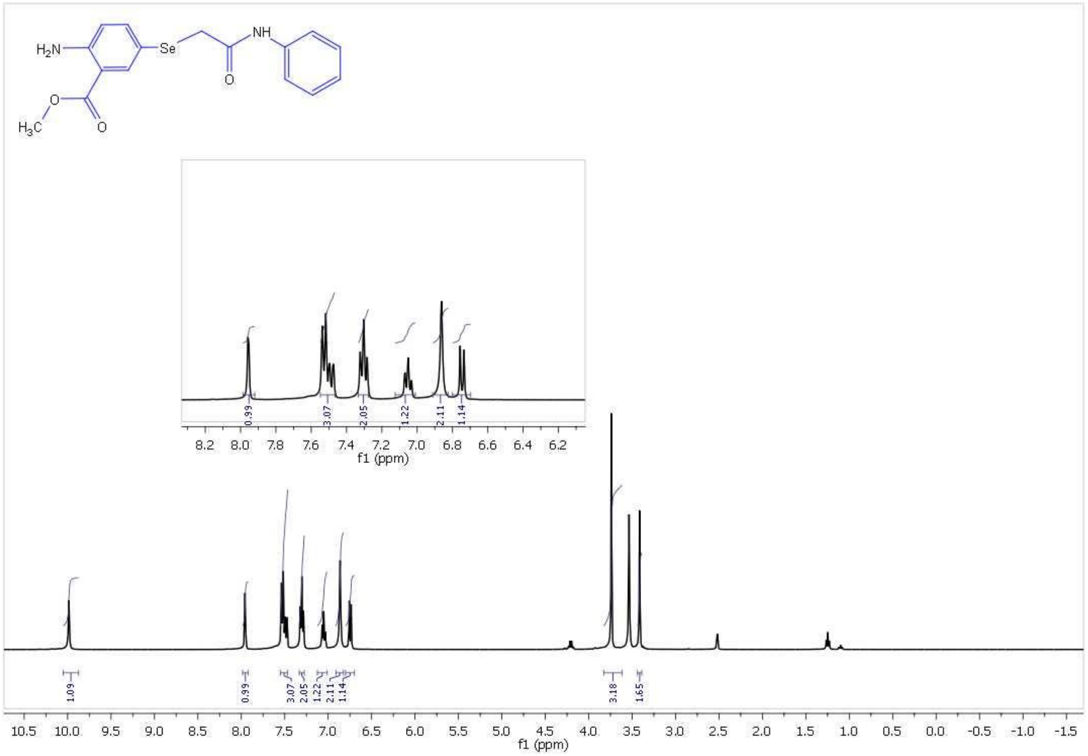

^{1}H NMR chart of compound **6**

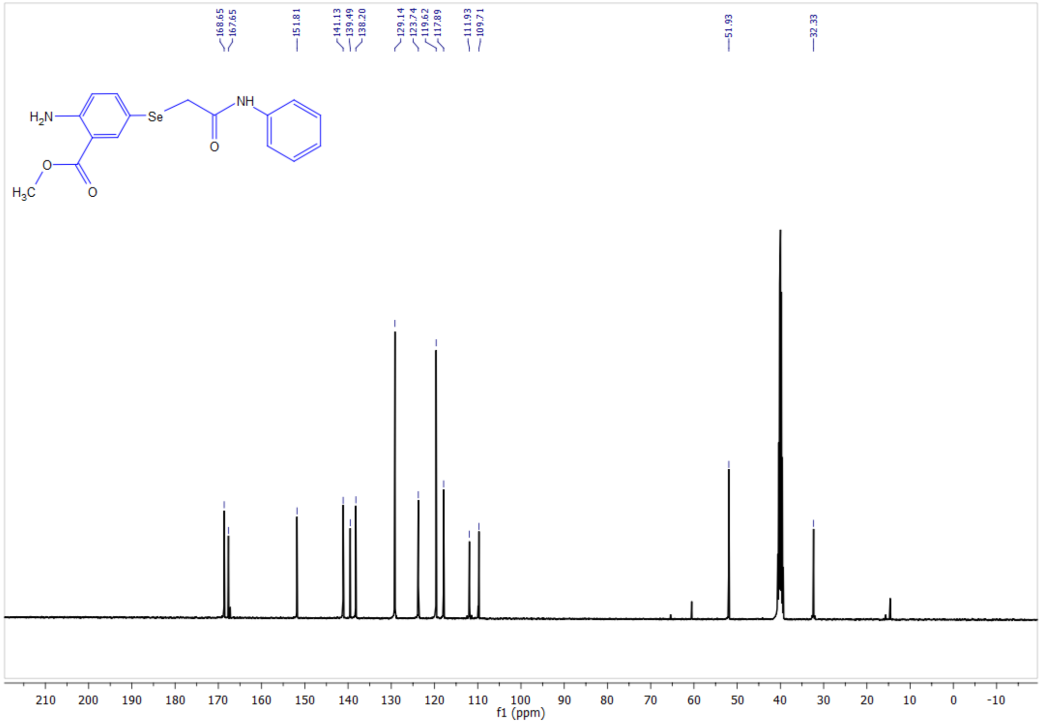

^{13}C NMR chart of compound **6**

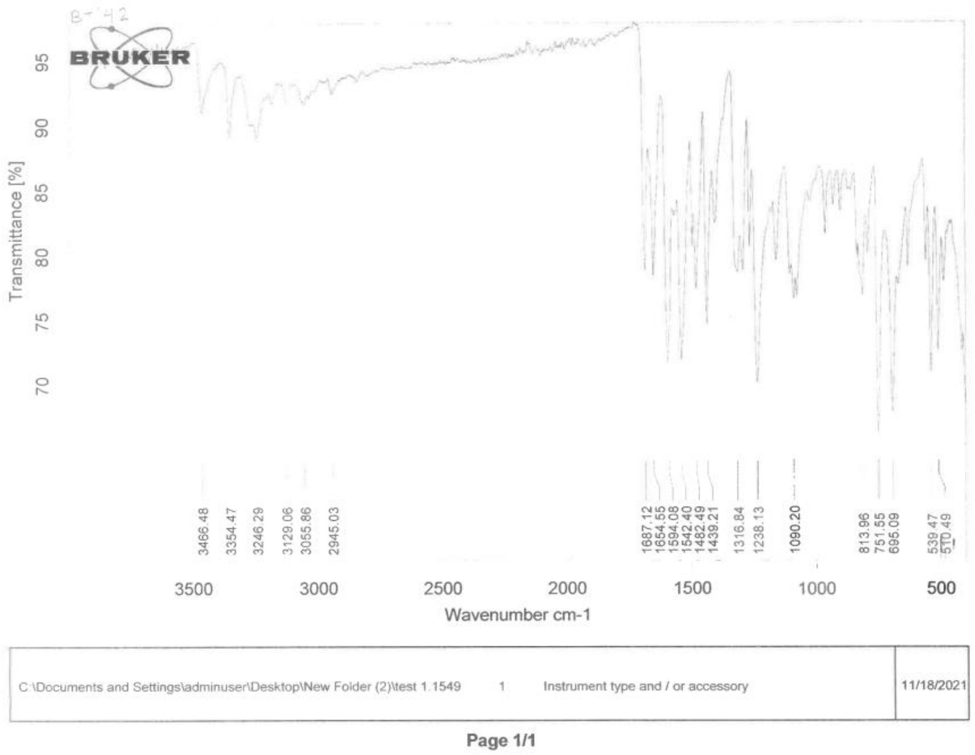

IR chart of compound **6**

Cairo University
Micro Analytical Center

DI Analysis
Shimadzu Qp-2010 Plus

Sample Information		Method	
Analysed by	Dr. Mai Younis	—— Analytical Line 1 ——	
Analyzed	06/01/2007 08:03:42 ‹	IonSourceTemp	250.00 °C
Sample Name	B23	[MS Table]	
Sample ID		--Group 1 - Event 1--	
Customer Name	Dr.Mohamed Soliman - Science - Cairo	Start Time	0.00min
Data File	C:\GCMSsolution\Data\Project1\B23.QGD	End Time	10.00min
Org Data File	C:\GCMSsolution\Data\Project1\B23.QGD	ACQ Mode	Scan
Method File	C:\GCMSsolution\Data\Project1\High Temperature Op	Event Time	0.50sec
Org Method File	C:\GCMSsolution\Data\Project1\High Temperature Op	Scan Speed	1000
Report File		Start m/z	50.00
Tuning File	C:\GCMSsolution\System\Tune1_default.qgt	End m/z	510.00
$End16Modified by	Dr. Mai Younis		
Modified	06/01/2007 08:06:25 ‹	Electron Voltage	70 eV
		Ionization Mode	EI

C:\GCMSsolution\Data\Project1\B23.QGD

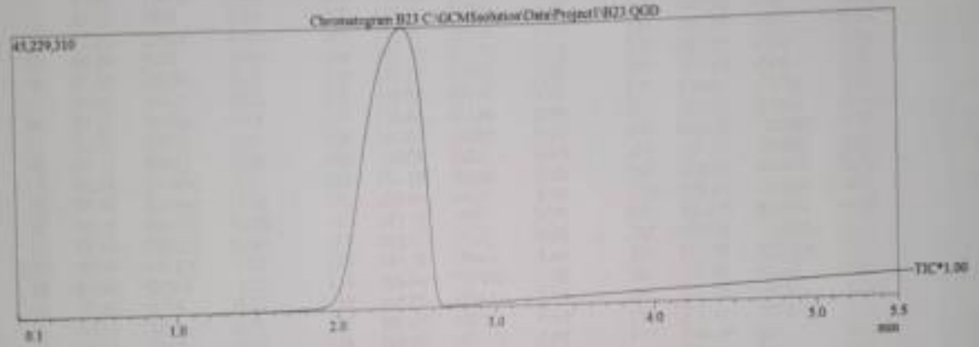

Spectrum

Line#:1 R.Time:2.6(Scan#:309)
MassPeaks:395(Peak Elimination m/z: 467.40, 468.40, 469.60, 473.60, 487.60)
RawMode:Single 2.6(309) BasePeak:364(2220999)
BG Mode:None Group 1 - Event 1

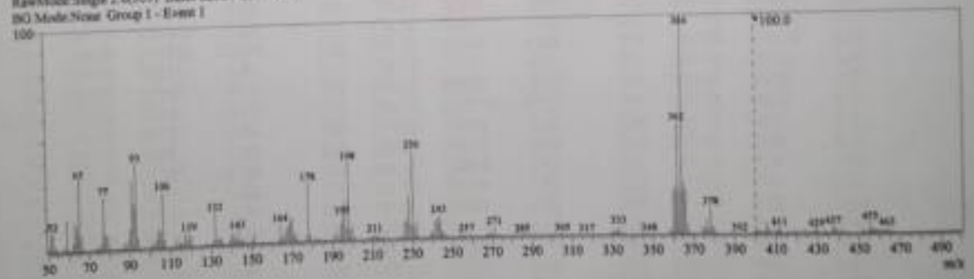

Mass Table
Line#:1 R.Time:2.6(Scan#:309)
MassPeaks:395(Peak Elimination m/z: 467.40, 468.40, 469.60, 473.60, 487.60)
RawMode:Single 2.6(309) BasePeak:364(2220999)
BG Mode:None Group 1 - Event 1

#	m/z	Abs. In	Rel. Int.	#	m/z	Abs. In	Rel. Int.	#	m/z	Abs. In	Rel. Int.
1	50.05	51768	2.33	4	53.00	56865	2.56	7	56.05	7933	0.36
2	51.00	187841	8.46	5	54.00	39409	1.77	8	57.05	37932	1.71
3	52.00	198514	8.94	6	55.00	21300	0.96	9	58.05	57716	2.60

1 / 3

Mass chart of compound **6**

#	m/z	Abs. In	Rel. Int.
10	59.00	323380	14.56
11	60.00	19518	0.88
12	61.05	25294	1.14
13	62.05	85663	3.86
14	63.05	284974	12.83
15	64.05	250676	11.29
16	65.00	718538	32.35
17	66.00	152620	6.87
18	66.95	28433	1.28
19	68.05	8877	0.40
20	69.05	15359	0.69
21	70.05	8547	0.38
22	71.05	14796	0.67
23	72.05	7256	0.33
24	73.05	29968	1.35
25	74.05	26922	1.21
26	75.05	38018	1.71
27	76.05	89648	4.04
28	77.05	535908	24.13
29	78.05	171575	7.73
30	79.05	144276	6.50
31	80.05	37639	1.69
32	81.05	11176	0.50
33	82.15	5419	0.24
34	83.10	15562	0.70
35	84.15	7935	0.36
36	85.10	16374	0.74
37	86.15	16002	0.72
38	87.10	69636	3.14
39	88.15	32733	1.47
40	89.15	88448	3.98
41	90.15	210874	9.49
42	91.10	688561	31.00
43	92.15	463765	20.88
44	93.10	862055	38.81
45	94.05	112159	5.05
46	95.00	27708	1.25
47	96.10	5274	0.24
48	97.10	9146	0.41
49	98.15	5237	0.24
50	99.10	12962	0.58
51	100.15	13487	0.61
52	101.10	60012	2.70
53	102.15	30642	1.38
54	103.15	88585	3.99
55	104.10	204116	9.19
56	105.15	159966	7.20
57	106.10	568420	25.59
58	107.10	76871	3.46
59	108.05	12580	0.57
60	109.10	3719	0.17
61	110.15	3039	0.14
62	111.15	9856	0.44
63	112.15	15347	0.69
64	113.10	93678	4.22
65	114.15	34219	1.54
66	115.10	49390	2.22
67	116.15	38664	1.74
68	117.10	130407	5.87
69	118.15	63644	2.87
70	119.10	142945	6.44
71	120.10	98100	4.42
72	121.05	14291	0.64
73	122.10	5969	0.27
74	123.05	3081	0.14
75	124.15	1538	0.07
76	125.15	3955	0.18
77	126.15	7068	0.32
78	127.15	19469	0.88

#	m/z	Abs. In	Rel. Int.
79	128.15	17920	0.81
80	129.15	16582	0.75
81	130.15	23991	1.08
82	131.15	62358	2.81
83	132.10	333977	15.04
84	133.15	66067	2.97
85	134.15	78340	3.53
86	135.15	69517	3.13
87	136.15	11769	0.53
88	137.10	7681	0.35
89	138.15	13090	0.59
90	139.15	52322	2.36
91	140.10	117575	5.29
92	141.05	90195	4.06
93	142.05	52676	2.37
94	143.00	141599	6.38
95	144.00	54009	2.43
96	145.05	43425	1.96
97	146.05	54032	2.43
98	147.10	12569	0.57
99	148.10	17756	0.80
100	149.15	8208	0.37
101	150.15	38743	1.74
102	151.10	102964	4.64
103	152.05	16710	0.75
104	153.10	11177	0.50
105	154.10	14711	0.66
106	155.05	15641	0.70
107	156.05	11100	0.50
108	157.05	15214	0.69
109	158.05	8233	0.37
110	159.10	16986	0.76
111	160.05	4503	0.20
112	161.15	4537	0.20
113	162.15	5173	0.23
114	163.15	35647	1.60
115	164.10	195450	8.80
116	165.05	50811	2.29
117	166.05	92270	4.15
118	167.05	149172	6.72
119	168.05	172761	7.78
120	169.05	226041	10.18
121	170.00	257615	11.60
122	171.00	96798	4.36
123	172.00	69998	3.15
124	173.00	21033	0.95
125	174.10	7897	0.36
126	175.15	17735	0.80
127	176.15	7737	0.35
128	177.15	62536	2.82
129	178.10	605762	27.27
130	179.05	72024	3.24
131	180.05	20815	0.94
132	181.10	26969	1.21
133	182.05	36544	1.65
134	183.10	28086	1.26
135	184.05	26423	1.19
136	185.00	19938	0.90
137	186.00	10751	0.48
138	187.05	6516	0.29
139	188.25	4750	0.21
140	189.15	25047	1.13
141	190.15	9994	0.45
142	191.15	57570	2.59
143	192.10	82754	3.73
144	193.05	33585	1.51
145	194.05	172249	7.76
146	195.05	249002	11.21
147	196.00	413670	18.63

#	m/z	Abs. In	Rel. Int.
148	197.05	127657	5.75
149	198.00	802486	36.13
150	198.95	111117	5.00
151	200.00	147049	6.62
152	201.00	27327	1.23
153	202.05	6591	0.30
154	203.15	3651	0.16
155	204.15	2634	0.12
156	205.10	8610	0.39
157	206.05	3512	0.16
158	207.05	11680	0.53
159	208.05	15928	0.72
160	209.05	33769	1.52
161	210.05	30291	1.36
162	211.05	55042	2.48
163	212.05	30408	1.37
164	213.05	31320	1.41
165	214.05	18869	0.85
166	215.05	9367	0.42
167	216.05	19840	0.89
168	217.05	4333	0.20
169	218.05	4479	0.20
170	219.10	2612	0.12
171	220.10	778	0.04
172	221.15	1397	0.06
173	222.15	1142	0.05
174	223.15	4881	0.22
175	224.05	17976	0.81
176	225.15	20916	0.94
177	226.10	174902	7.87
178	227.15	189200	8.52
179	228.05	447454	20.15
180	229.15	133404	6.01
181	230.05	899020	40.48
182	231.05	114924	5.17
183	232.05	173318	7.80
184	233.05	21637	0.97
185	234.05	3484	0.16
186	235.15	3179	0.14
187	236.15	3807	0.17
188	237.05	8985	0.40
189	238.15	10887	0.49
190	239.10	48174	2.17
191	240.10	75681	3.41
192	241.10	157647	7.10
193	242.15	186228	8.38
194	243.10	230793	10.39
195	244.05	179124	8.07
196	245.05	65413	2.95
197	246.05	36727	1.65
198	247.20	22822	1.03
199	248.15	7827	0.35
200	249.15	2124	0.10
201	250.25	1855	0.08
202	251.20	7351	0.33
203	252.15	3123	0.14
204	253.15	6626	0.30
205	254.15	7024	0.32
206	255.10	19212	0.87
207	256.15	19273	0.87
208	257.10	29007	1.31
209	258.15	10175	0.46
210	259.20	21718	0.98
211	260.15	6061	0.27
212	261.15	4356	0.20
213	262.15	3391	0.15
214	263.15	3353	0.15
215	264.10	1687	0.08
216	265.15	3515	0.16

Mass chart of compound **6**

#	m/z	Abs. In	Rel. Int.
217	266.15	2637	0.12
218	267.10	18146	0.82
219	268.15	20158	0.91
220	269.10	46786	2.11
221	270.15	15694	0.71
222	271.10	96137	4.33
223	272.05	16070	0.72
224	273.10	21305	0.96
225	274.05	3890	0.18
226	275.15	1899	0.09
227	276.25	1345	0.06
228	277.20	2796	0.13
229	278.15	1435	0.06
230	279.15	898	0.04
231	280.15	727	0.03
232	281.15	2478	0.11
233	282.15	2409	0.11
234	283.10	5790	0.26
235	284.15	3531	0.16
236	285.10	12303	0.55
237	286.15	5148	0.23
238	287.10	5833	0.26
239	288.25	2633	0.12
240	289.15	7201	0.32
241	290.15	2596	0.12
242	291.20	2186	0.10
243	292.15	1131	0.05
244	293.25	1594	0.07
245	294.25	1118	0.05
246	295.20	5345	0.24
247	296.15	1761	0.08
248	297.25	811	0.04
249	298.35	617	0.03
250	299.30	823	0.04
251	300.25	1273	0.06
252	301.25	1429	0.06
253	302.25	2061	0.09
254	303.30	3346	0.15
255	304.35	3895	0.18
256	305.30	14001	0.63
257	306.20	5601	0.25
258	307.15	2298	0.10
259	308.15	902	0.04
260	309.30	892	0.04
261	310.25	583	0.03
262	311.35	721	0.03
263	312.35	712	0.03
264	313.35	1160	0.05
265	314.35	803	0.04
266	315.35	918	0.04
267	316.35	904	0.04
268	317.30	4386	0.20
269	318.25	2391	0.11
270	319.25	2801	0.13
271	320.25	1770	0.08
272	321.20	4260	0.19
273	322.25	2058	0.09
274	323.25	2501	0.11
275	324.25	848	0.04
276	325.15	508	0.02
277	326.15	836	0.04
278	327.15	2834	0.13
279	328.15	6050	0.27
280	329.15	23275	1.05
281	330.15	29151	1.31
282	331.15	50733	2.28
283	332.15	33893	1.53
284	333.15	88344	3.98
285	334.05	24078	1.08
286	335.15	19856	0.89
287	336.15	4538	0.20
288	337.15	1006	0.05
289	338.25	662	0.03
290	339.30	1508	0.07
291	340.25	578	0.03
292	341.25	684	0.03
293	342.25	584	0.03
294	343.25	805	0.04
295	344.20	1958	0.09
296	345.25	2706	0.12
297	346.20	3890	0.18
298	347.25	4640	0.21
299	348.20	7434	0.33
300	349.15	2578	0.12
301	350.15	2611	0.12
302	351.20	977	0.04
303	352.15	764	0.03
304	353.25	847	0.04
305	354.15	553	0.02
306	355.25	657	0.03
307	356.25	704	0.03
308	357.25	3443	0.16
309	358.20	42783	1.93
310	359.25	41697	1.88
311	360.20	448631	20.20
312	361.25	490508	22.09
313	362.20	113024	50.89
314	363.25	433043	19.50
315	364.20	222099	100.00
316	365.15	516139	23.24
317	366.15	453240	20.41
318	367.15	94607	4.26
319	368.15	13453	0.61
320	369.15	2332	0.10
321	370.30	4468	0.20
322	371.25	1831	0.08
323	372.20	5146	0.23
324	373.25	5093	0.23
325	374.25	54806	2.47
326	375.25	61887	2.79
327	376.20	144442	6.50
328	377.25	48025	2.16
329	378.20	271025	12.20
330	379.15	67760	3.05
331	380.20	56982	2.57
332	381.15	13270	0.60
333	382.15	2182	0.10
334	383.20	484	0.02
335	384.20	236	0.01
336	386.20	215	0.01
337	387.30	279	0.01
338	388.25	396	0.02
339	389.35	651	0.03
340	390.25	1290	0.06
341	391.35	1022	0.05
342	392.30	1966	0.09
343	393.35	1297	0.06
344	394.30	940	0.04
345	395.30	473	0.02
346	396.45	384	0.02
347	397.50	1070	0.05
348	398.45	585	0.03
349	399.45	317	0.01
350	400.20	370	0.02
351	401.20	289	0.01
352	402.20	250	0.01
353	403.20	257	0.01
354	404.45	336	0.02
355	405.40	1146	0.05
356	406.35	447	0.02
357	407.40	271	0.01
358	409.45	410	0.02
359	410.50	282	0.01
360	411.50	583	0.03
361	412.50	284	0.01
362	415.50	254	0.01
363	417.50	207	0.01
364	418.50	257	0.01
365	419.50	354	0.02
366	421.50	326	0.01
367	423.50	396	0.02
368	425.50	250	0.01
369	426.50	212	0.01
370	428.35	430	0.02
371	429.30	458	0.02
372	430.30	231	0.01
373	432.30	222	0.01
374	433.30	212	0.01
375	434.30	201	0.01
376	436.40	250	0.01
377	437.45	688	0.03
378	438.35	497	0.02
379	439.50	431	0.02
380	441.50	303	0.01
381	442.50	233	0.01
382	444.50	210	0.01
383	451.50	289	0.01
384	452.50	233	0.01
385	454.45	304	0.01
386	455.40	1105	0.05
387	456.35	499	0.02
388	457.10	265	0.01
389	458.15	443	0.02
390	459.10	218	0.01
391	460.20	433	0.02
392	462.20	287	0.01
393	463.40	473	0.02
394	464.40	252	0.01
395	466.40	212	0.01

3 / 3

Mass chart of compound **6**

4-((2-(methoxycarbonyl)-4-(methylselanyl)phenyl)amino)-4-oxobut-2-enoic acid (7)

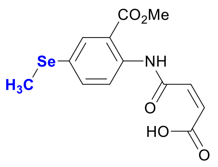

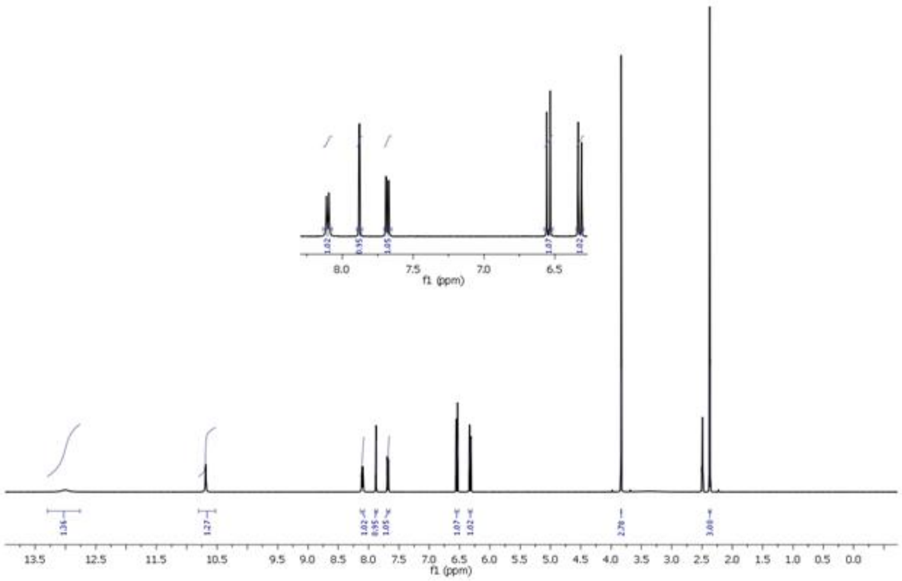

^{1}H NMR chart of compound **7**

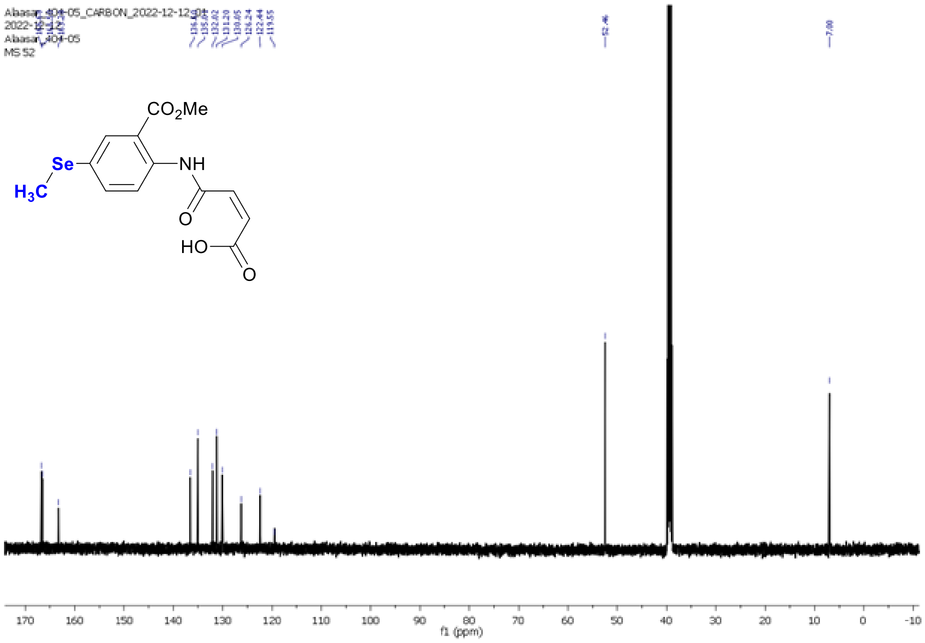

^{13}C NMR chart of compound **7**

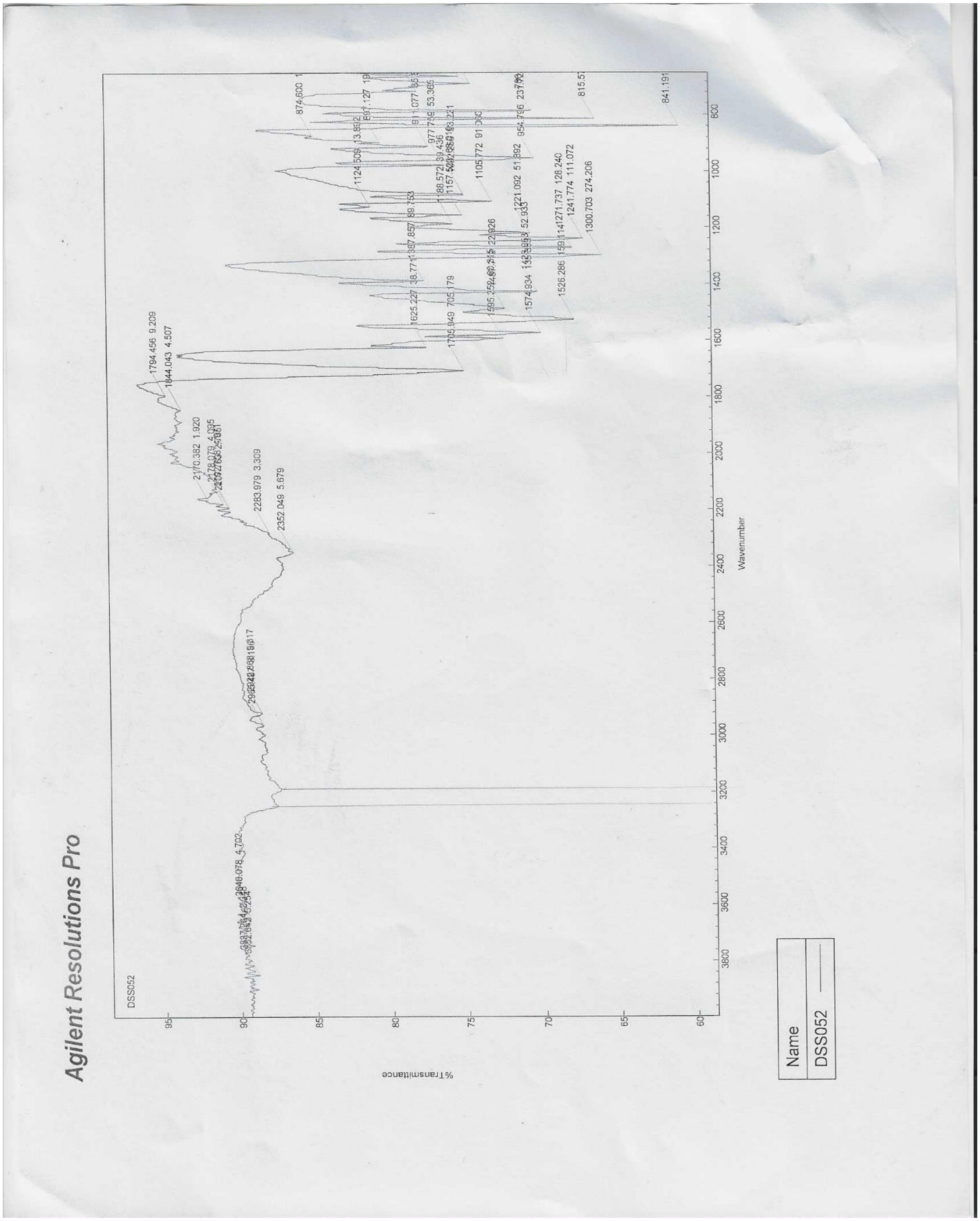

IR chart of compound **7**

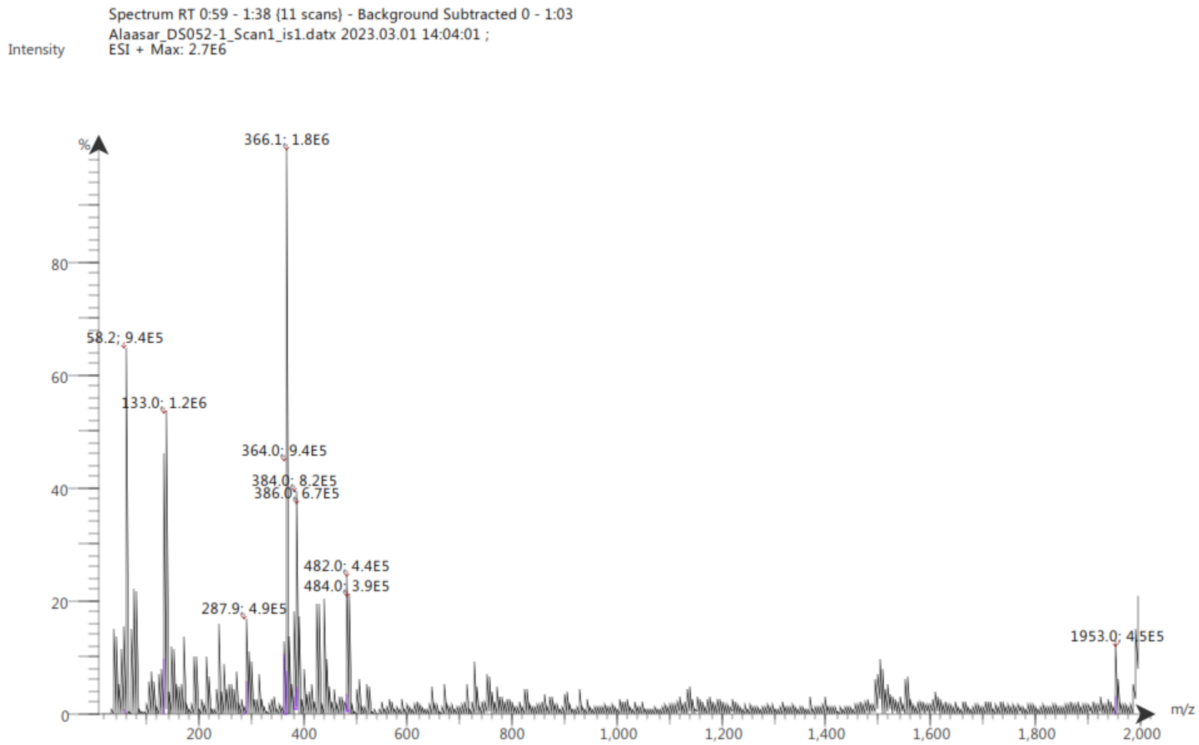

MS chart of compound **7**

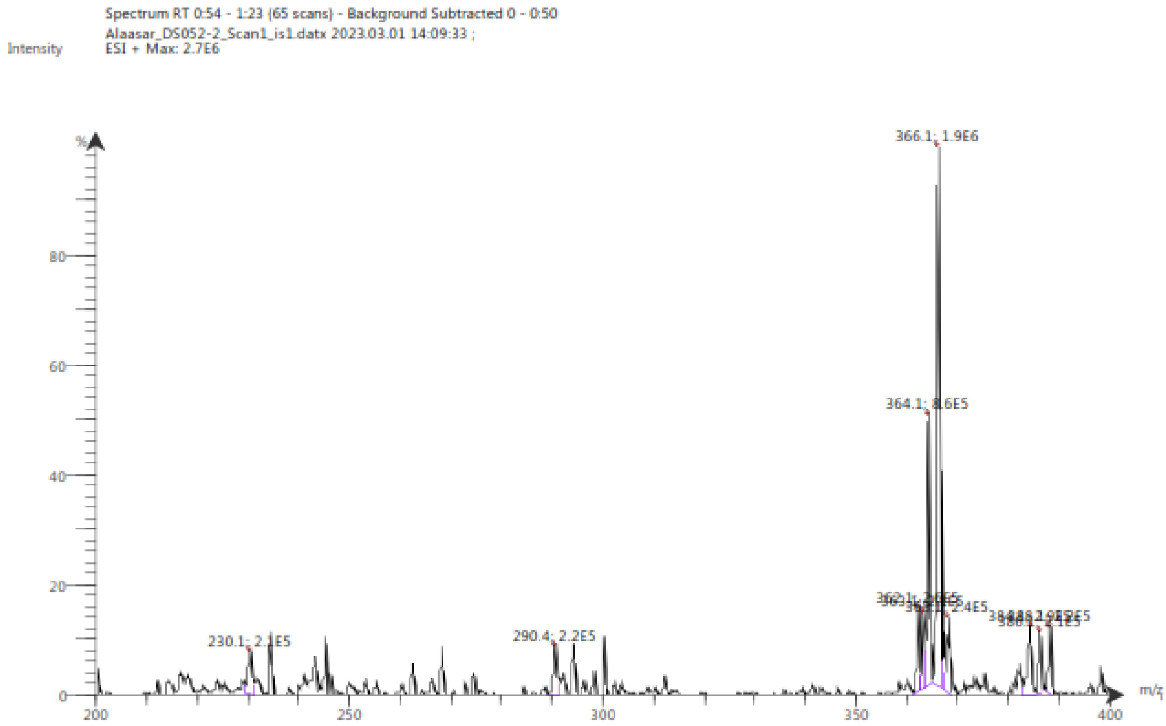

MS chart of compound **7**

4-((2-(methoxycarbonyl)-4-(methylselanyl)phenyl)amino)-4-oxobut-2-enoic acid (8)

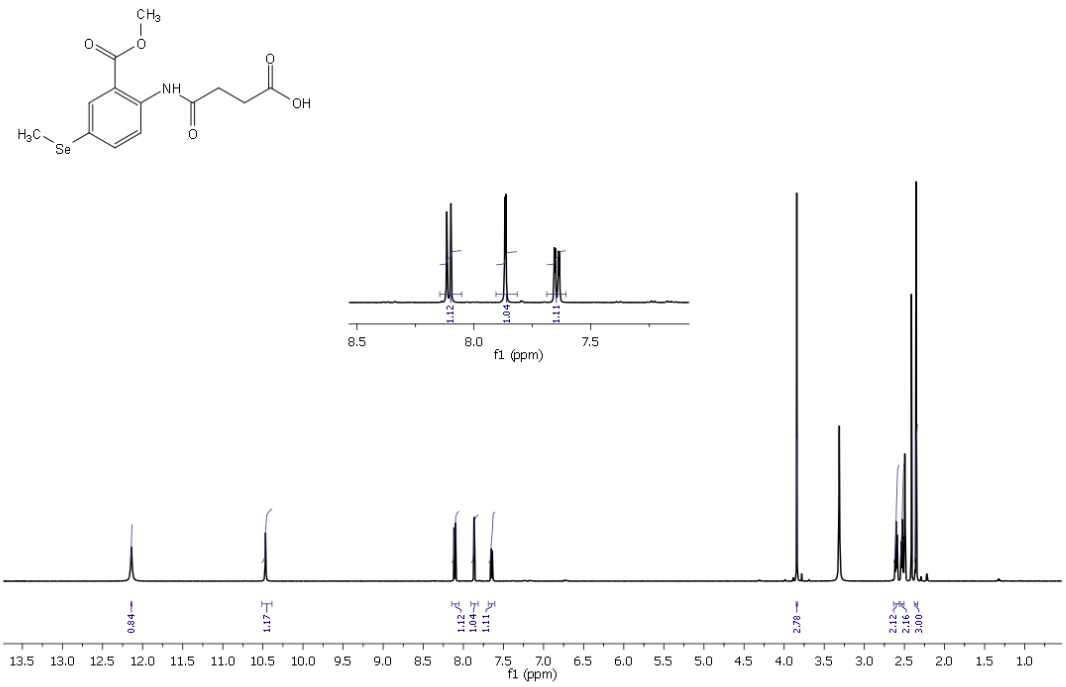

^{1}H NMR chart of compound **8**

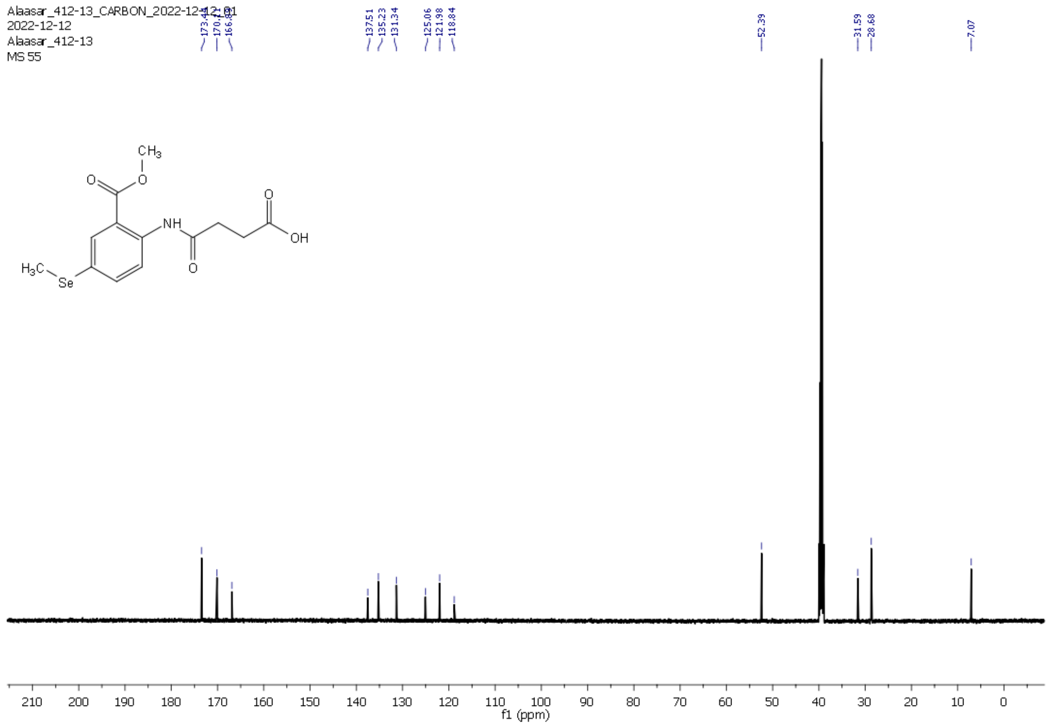

^{13}C NMR chart of compound **8**

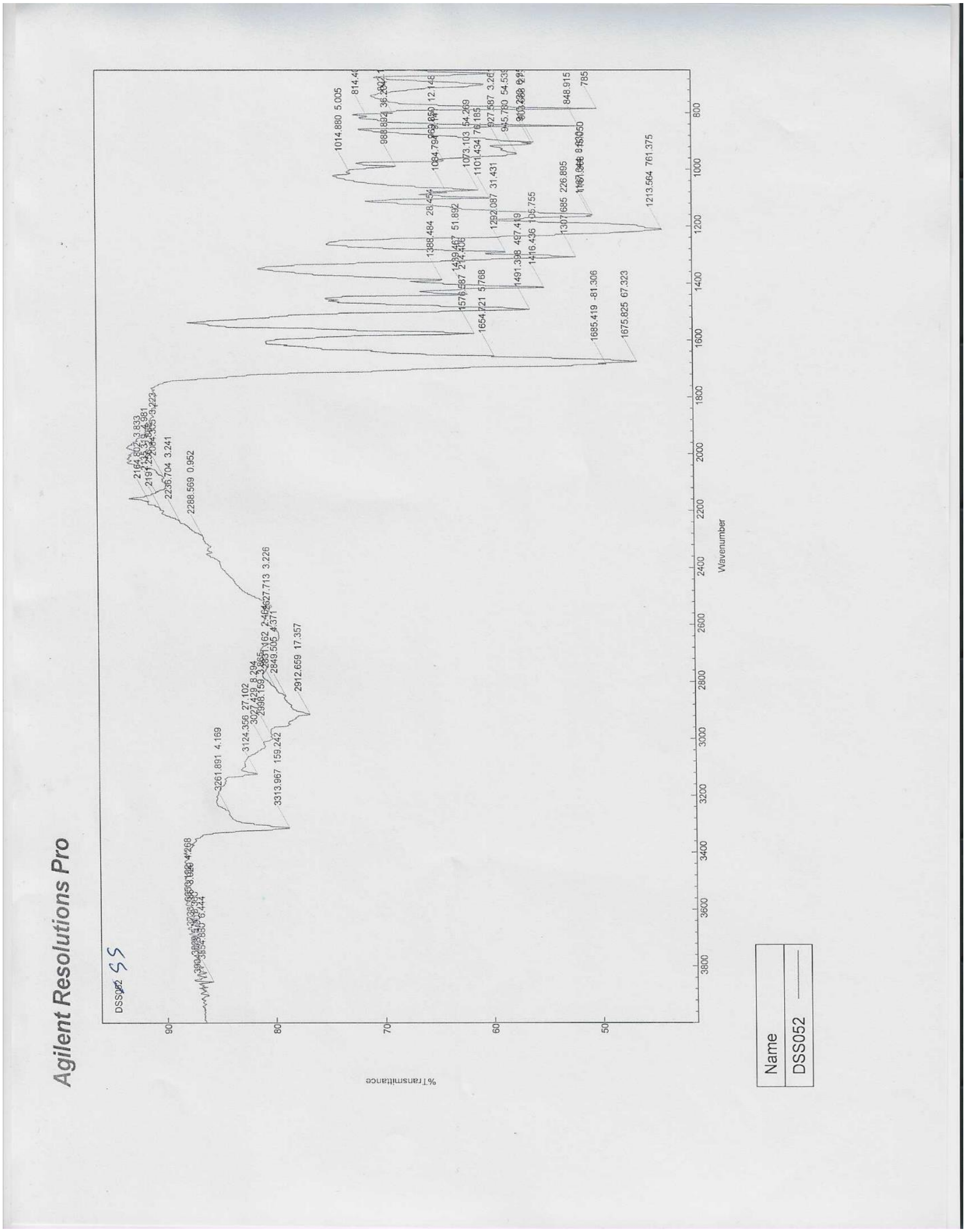

IR chart of compound **8**

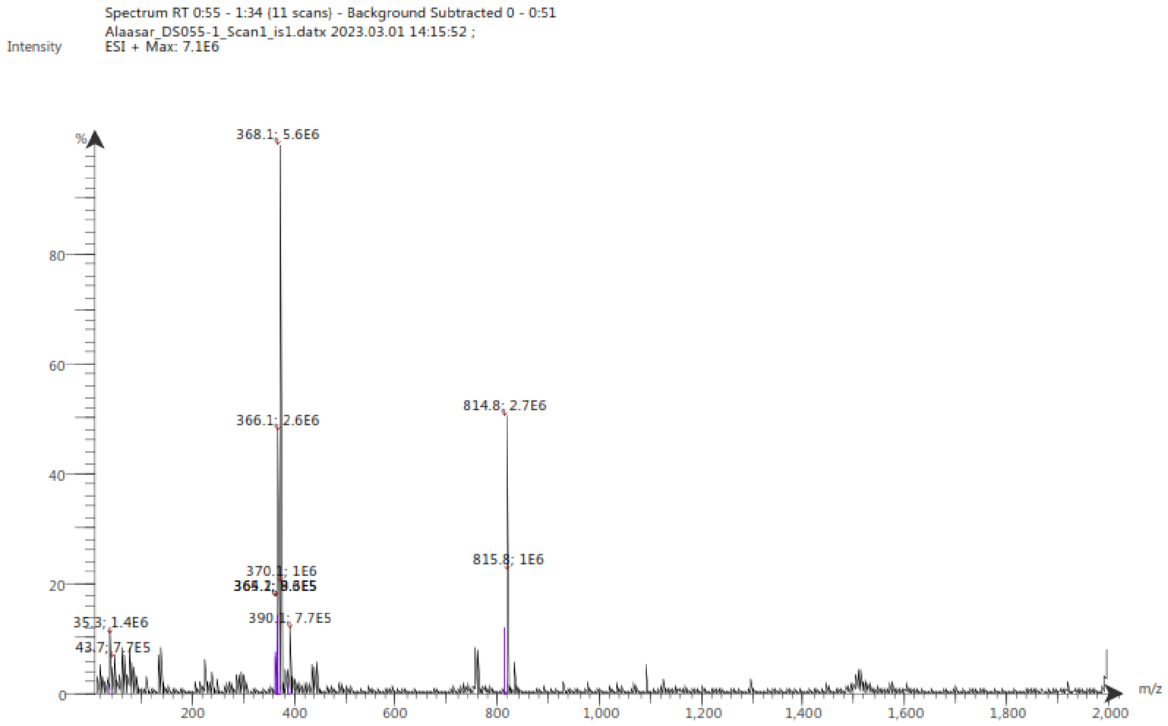

MS chart of compound **8**

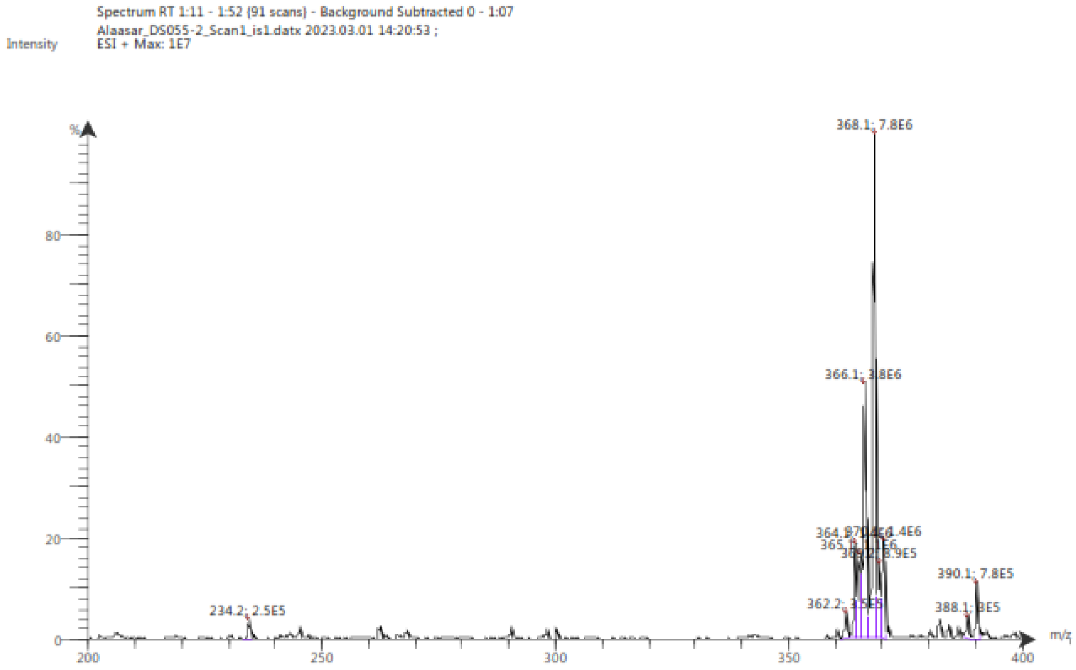

MS chart of compound **8**

4-((2-(methoxycarbonyl)-4-(methylselanyl)phenyl)amino)-4-oxobut-2-enoic acid (9)

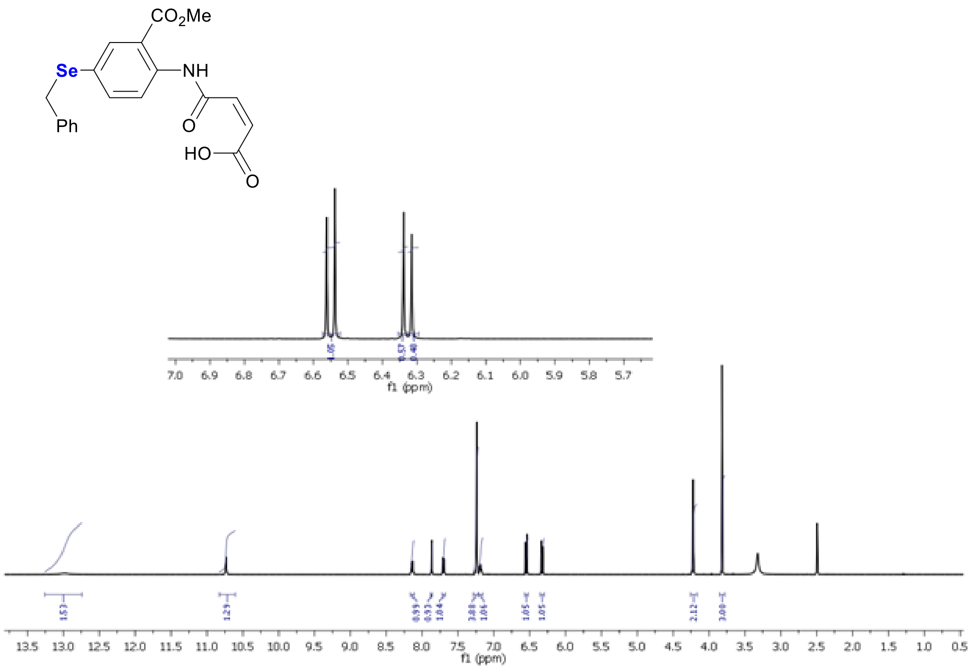

^{1}H NMR chart of compound **9**

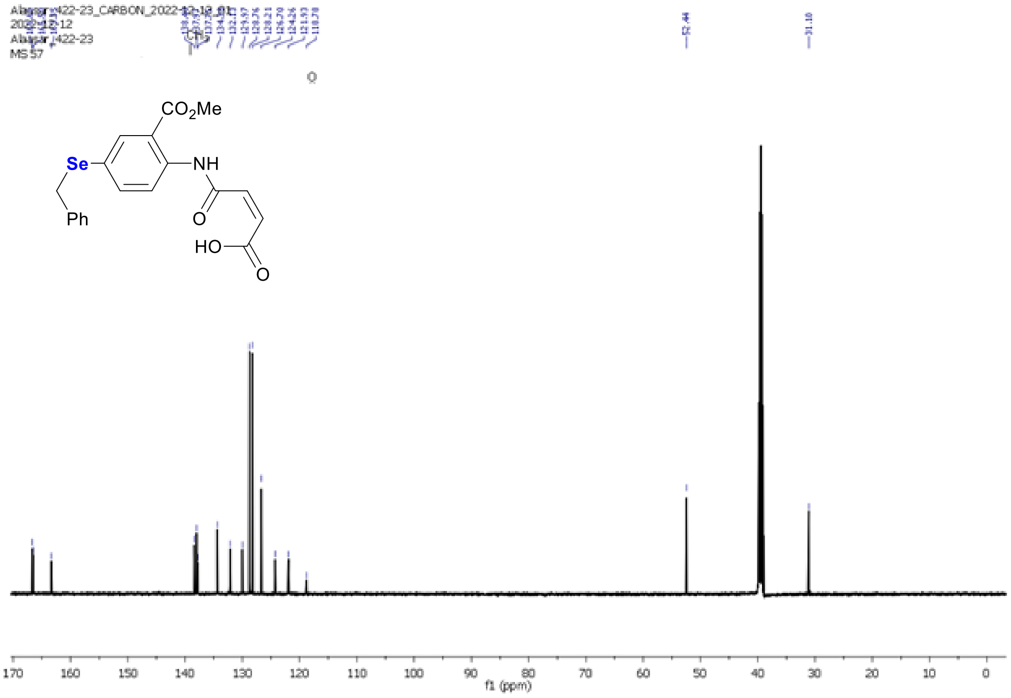

^{13}C NMR chart of compound **9**

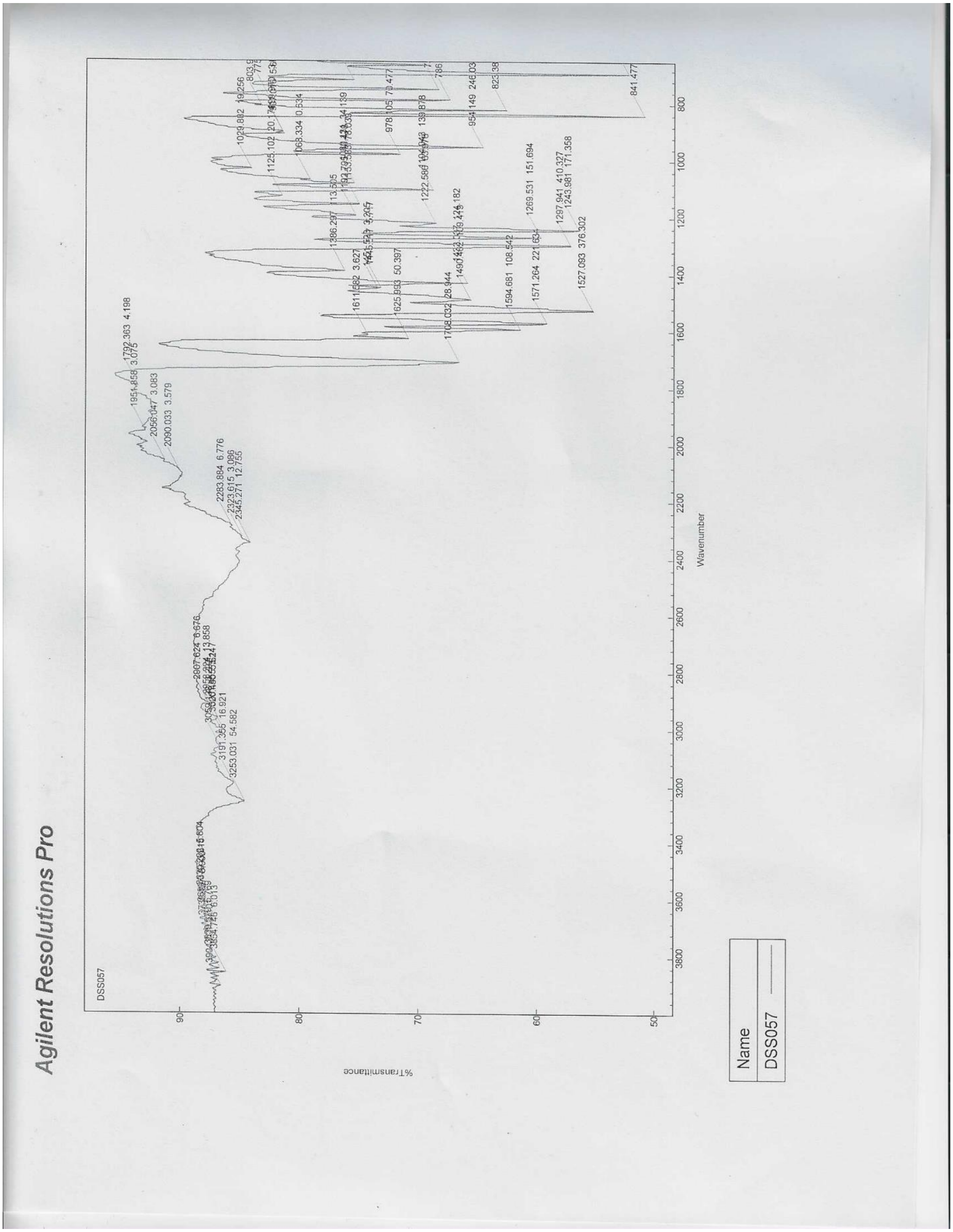

IR chart of compound **9**

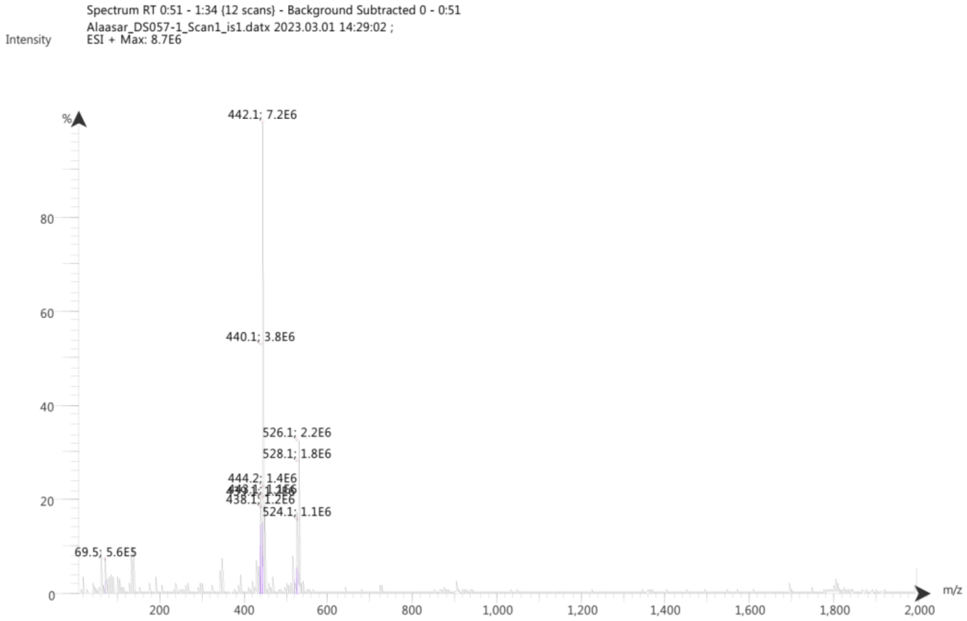

MS chart of compound **9**

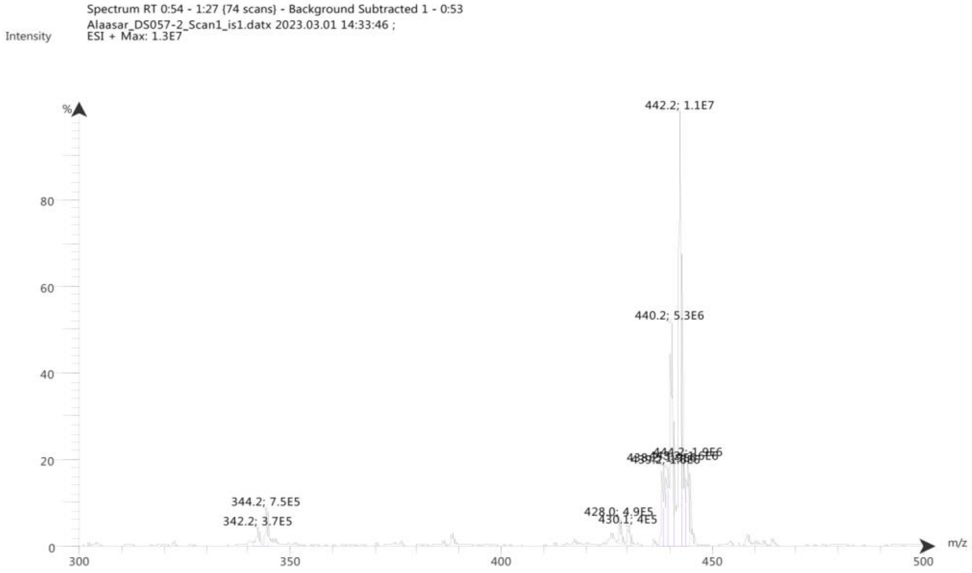

MS chart of compound **9**

4-((2-(methoxycarbonyl)-4-(methylselanyl)phenyl)amino)-4-oxobut-2-enoic acid (10)

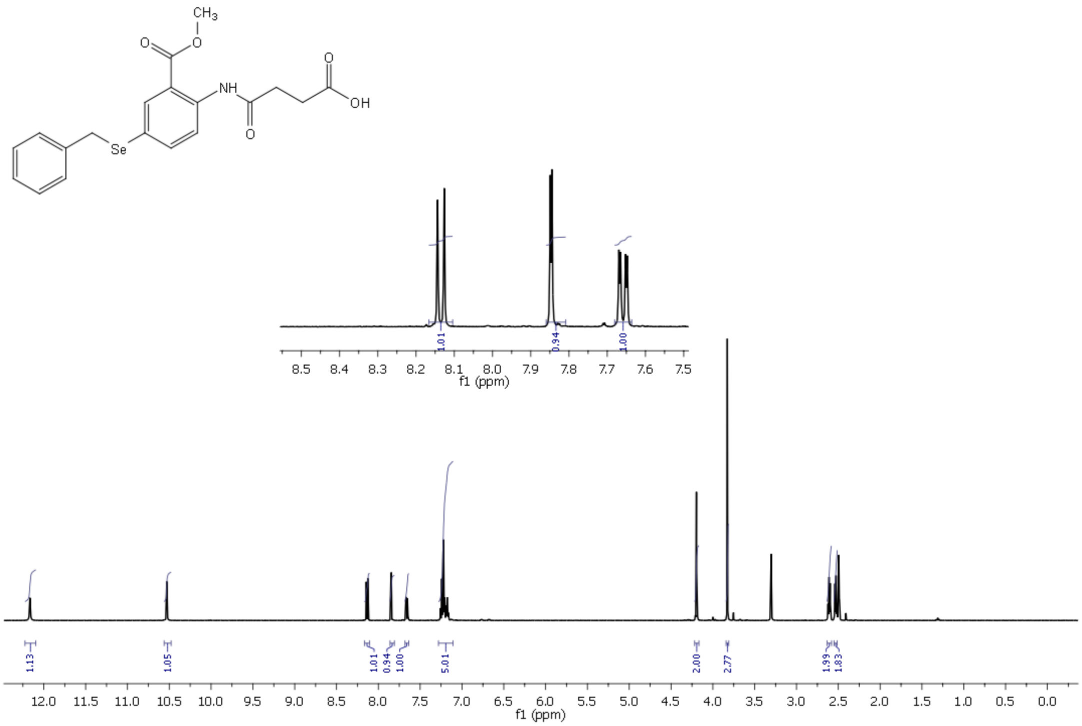

^{1}H NMR chart of compound **10**

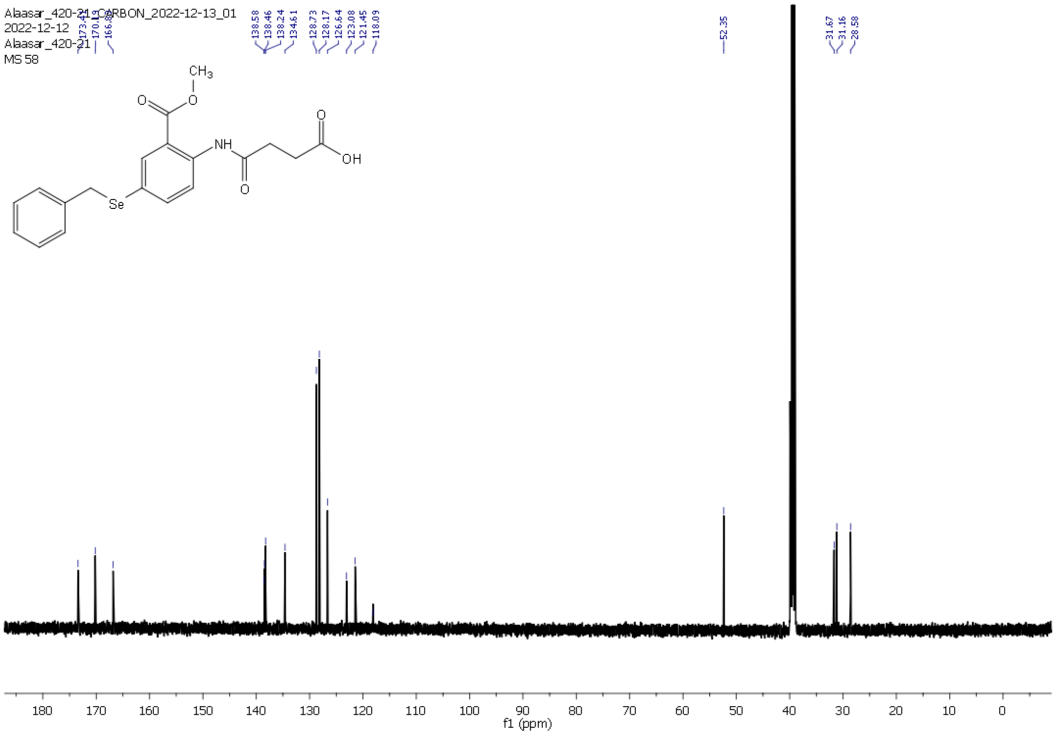

^{13}C NMR chart of compound **10**

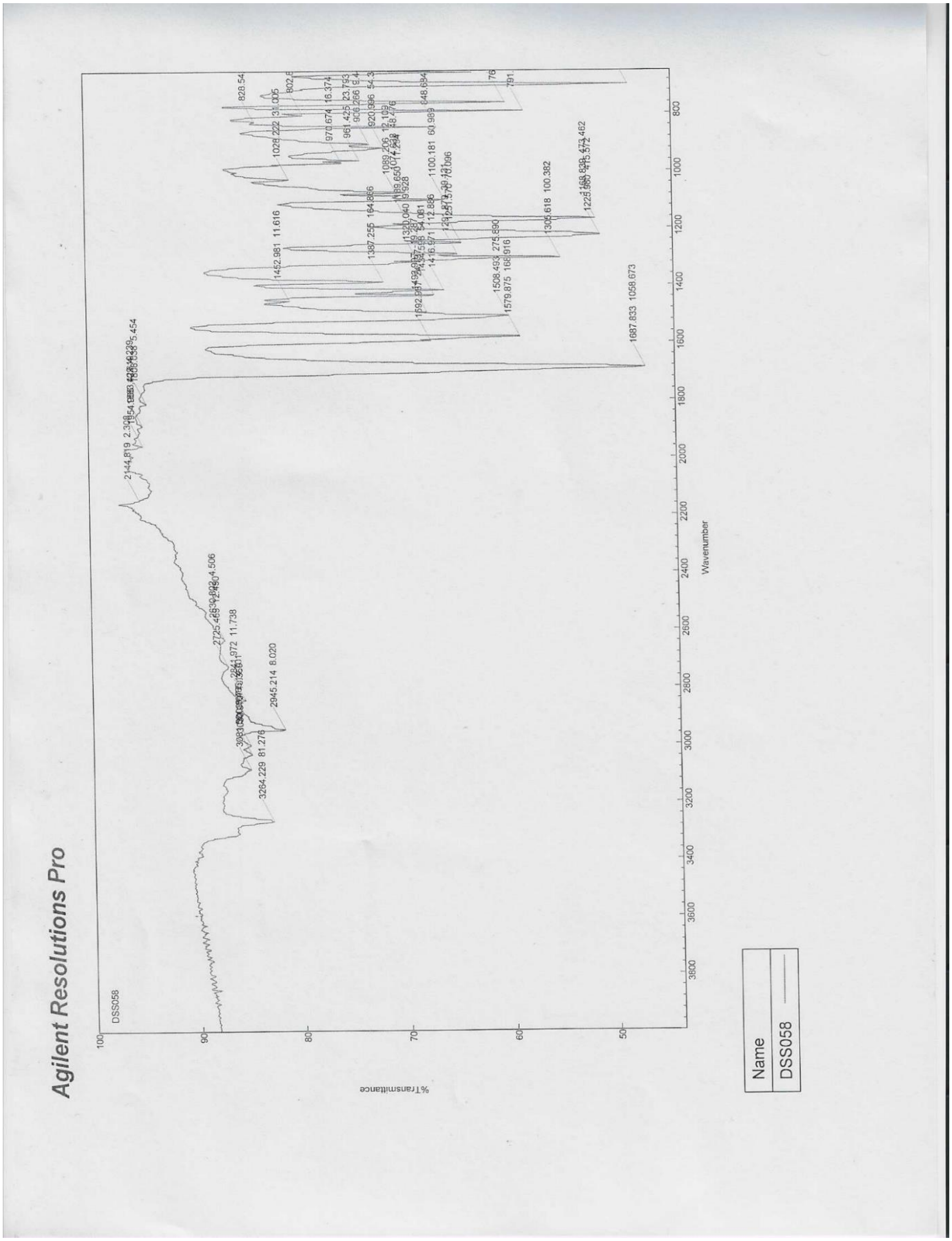

IR chart of compound **10**

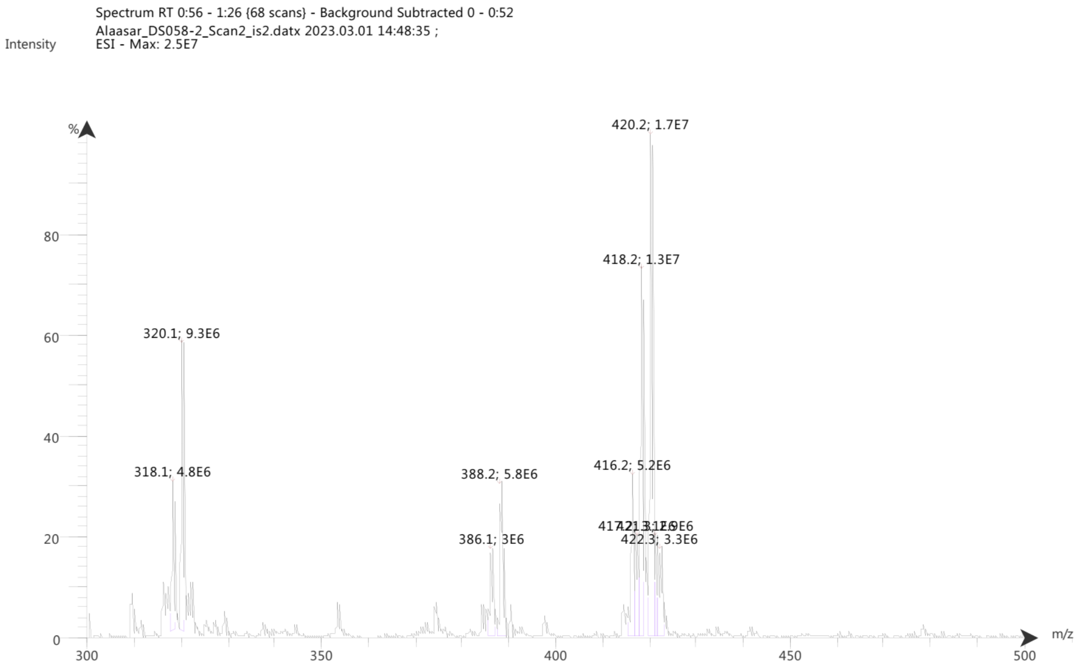

MS chart of compound **10**

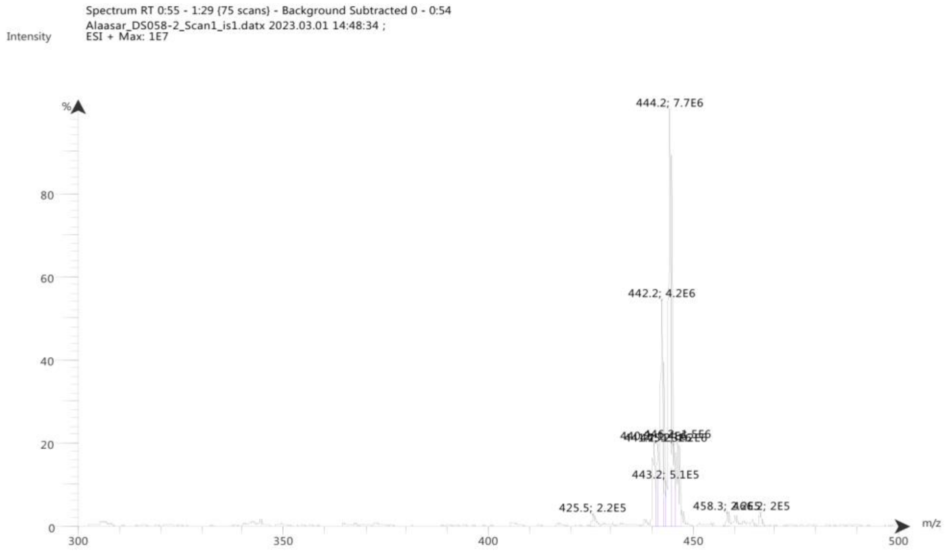

MS chart of compound **10**

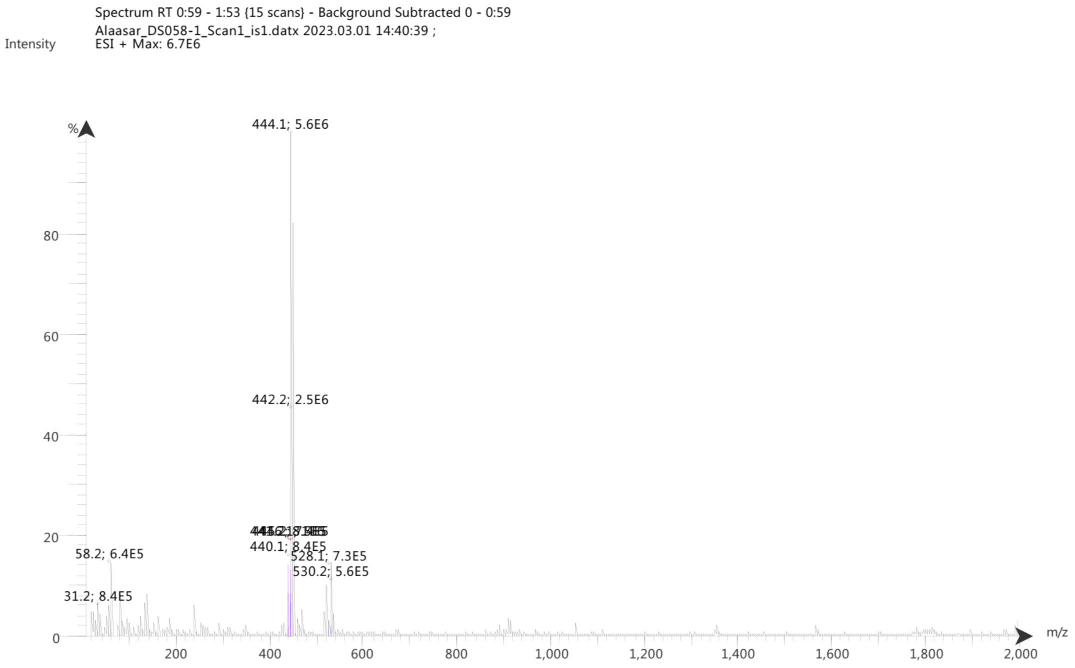

MS chart of compound **10**

4-((2-(methoxycarbonyl)-4-(methylselanyl)phenyl)amino)-4-oxobut-2-enoic acid (11)

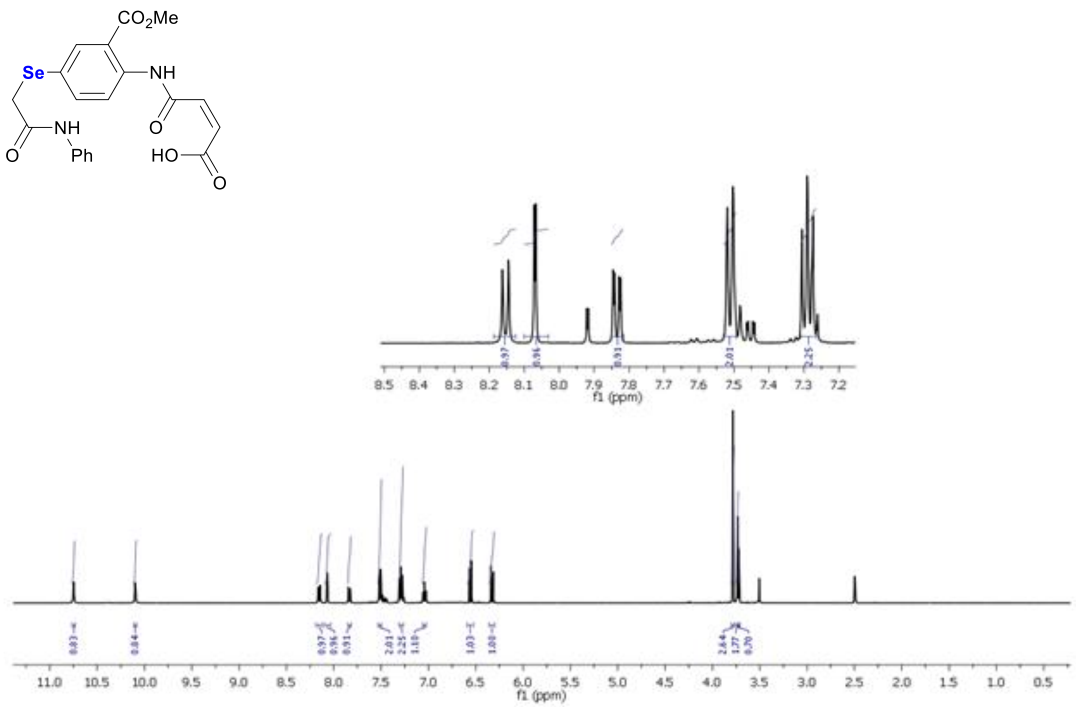

^{1}H NMR chart of compound **11**

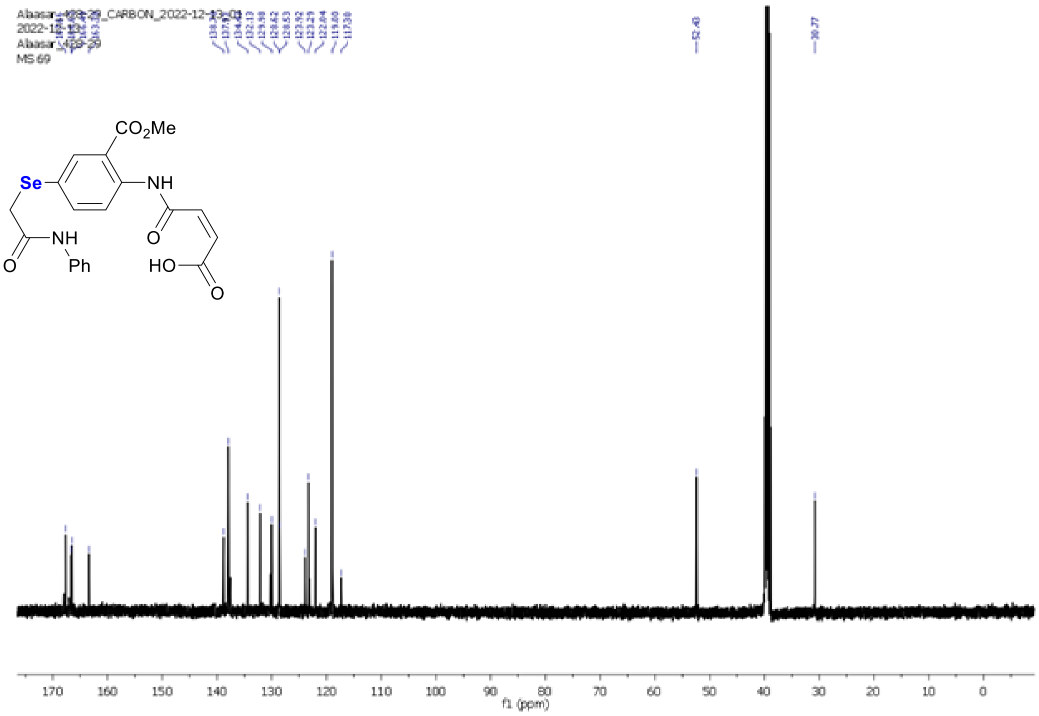

^{13}C NMR chart of compound **11**

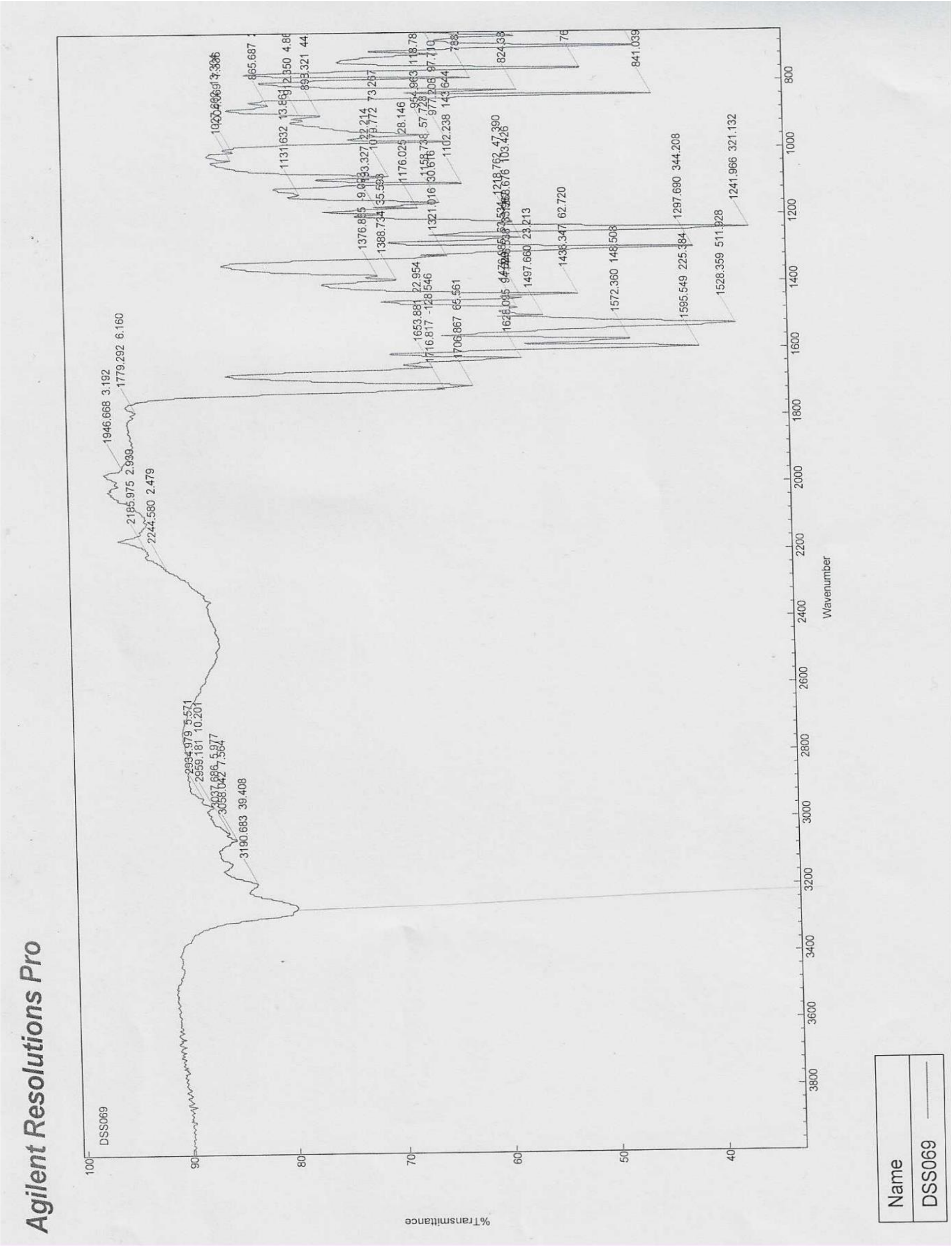

IR chart of compound **11**

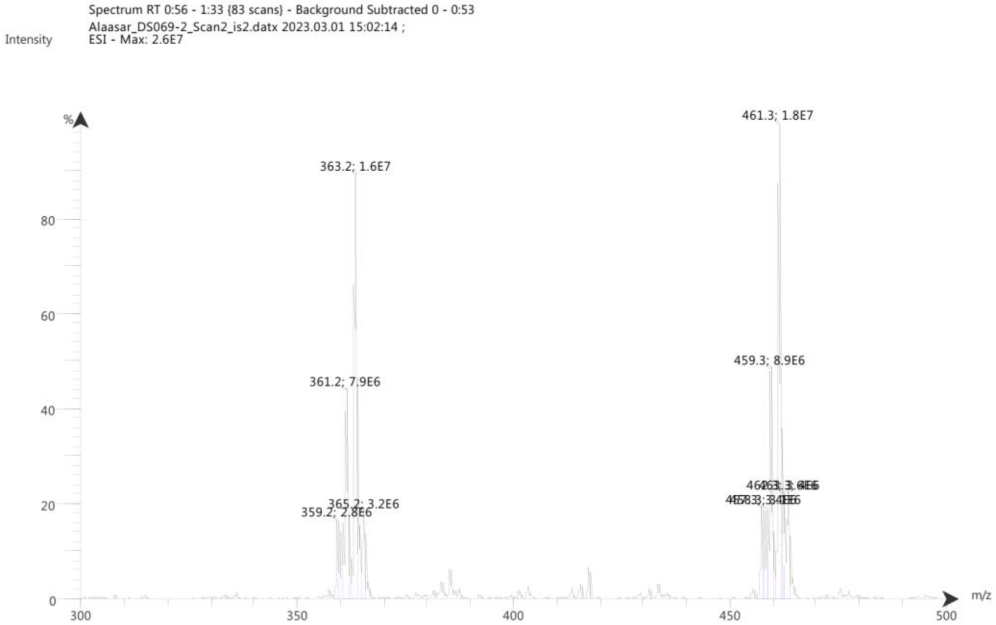

MS chart of compound **11**

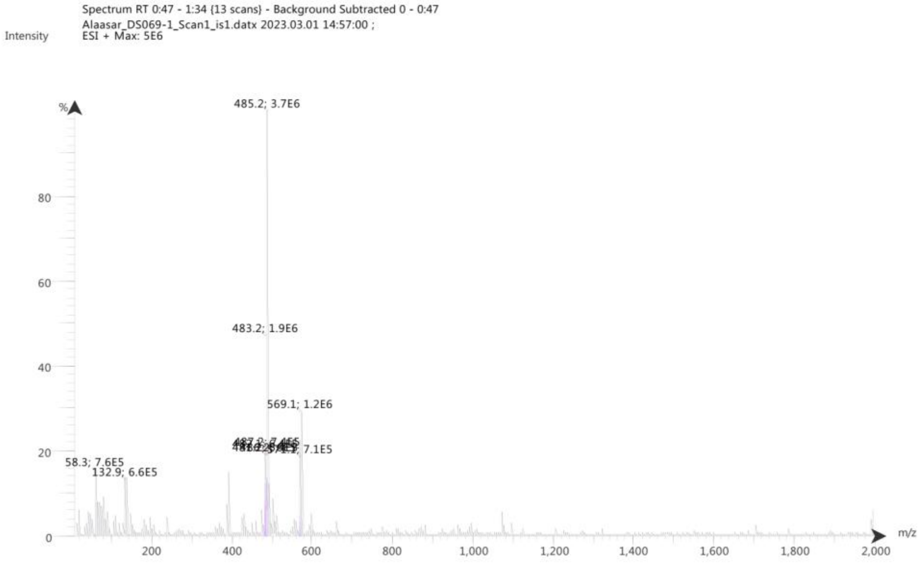

MS chart of compound **11**

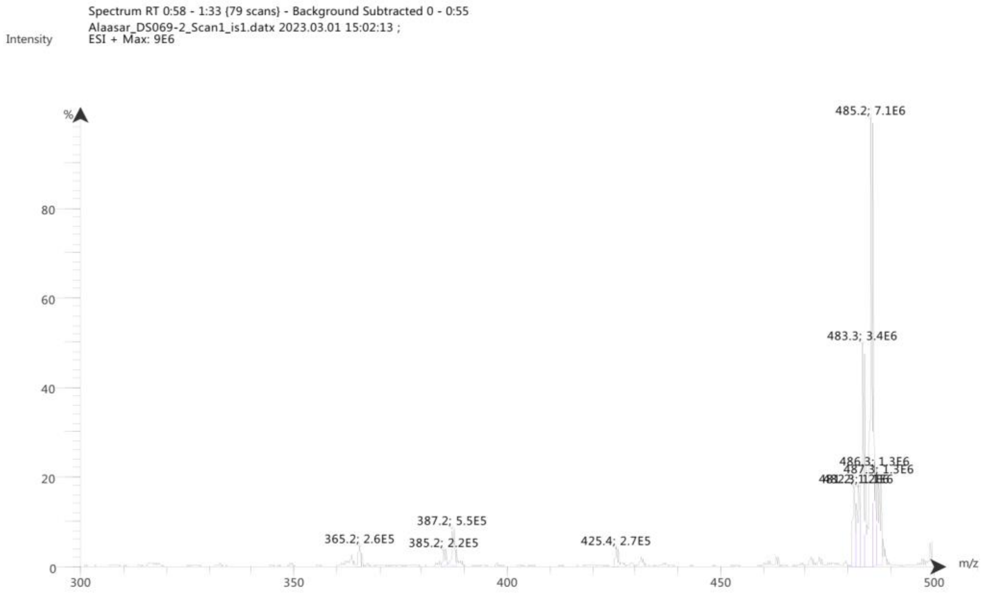

MS chart of compound **11**

4-((2-(methoxycarbonyl)-4-(methylselanyl)phenyl)amino)-4-oxobut-2-enoic acid (12)

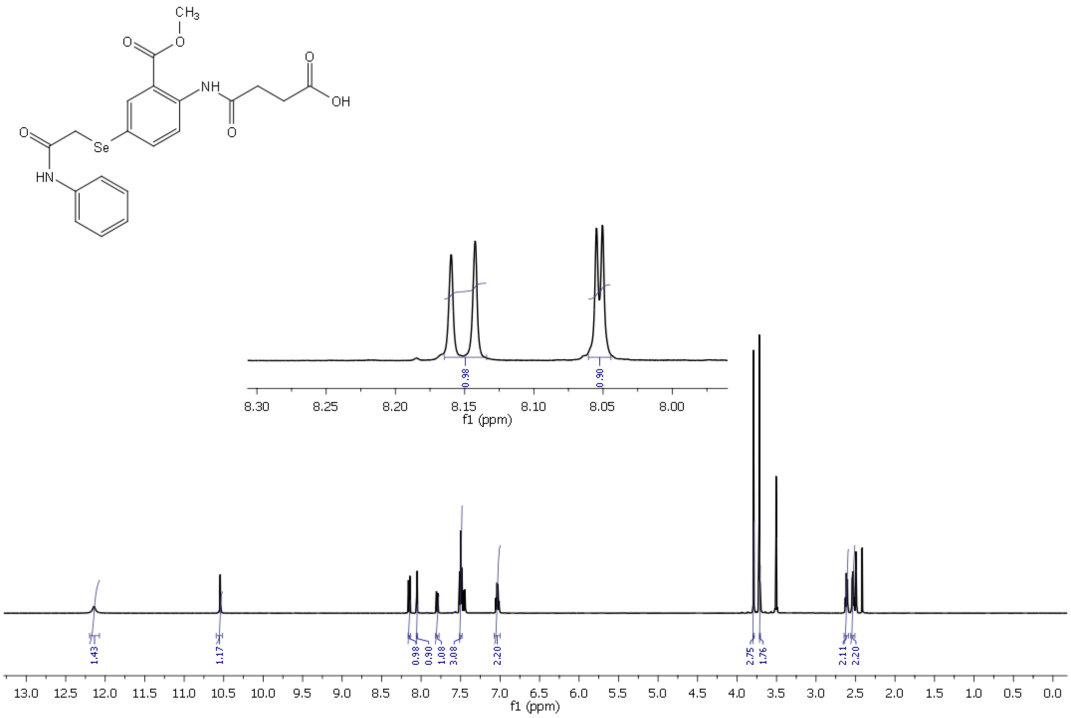

^{1}H NMR chart of compound **12**

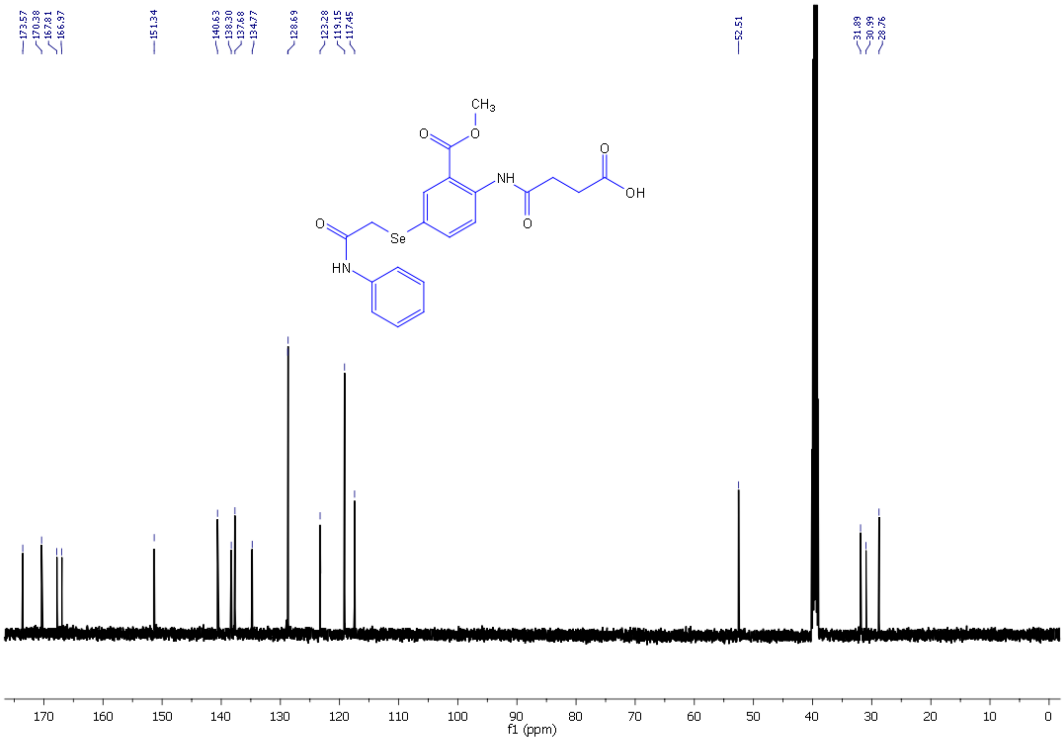

^{13}C NMR chart of compound **12**

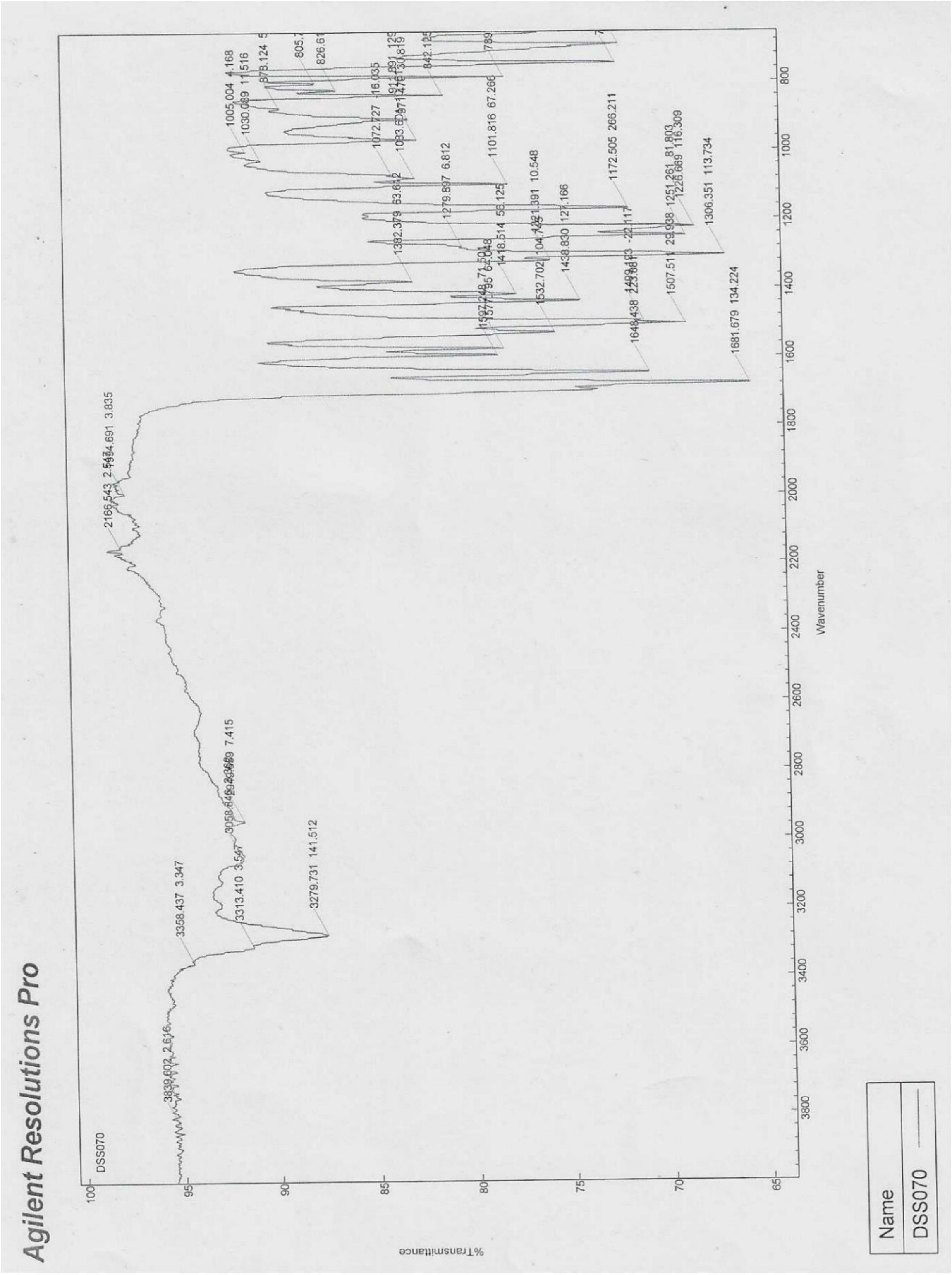

IR chart of compound **12**

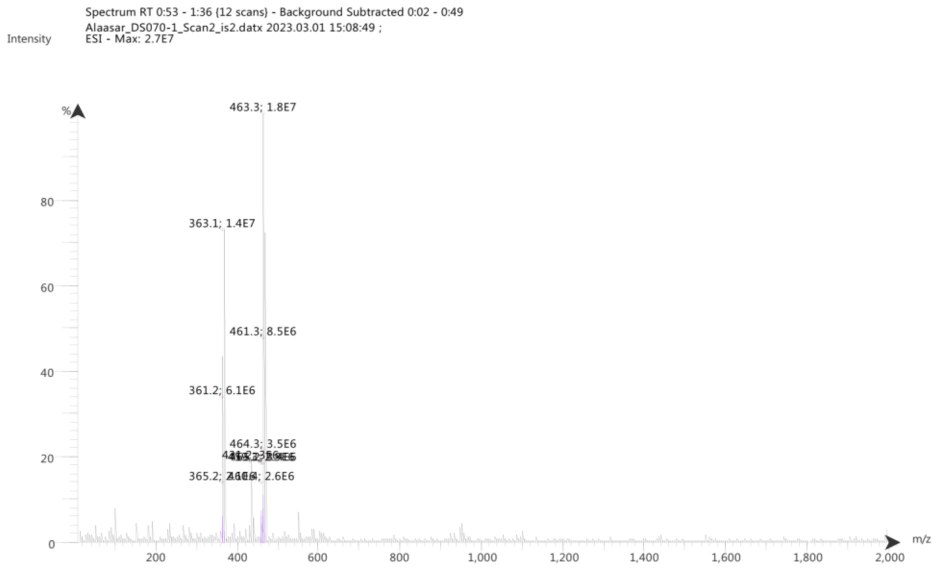

MS chart of compound **12**

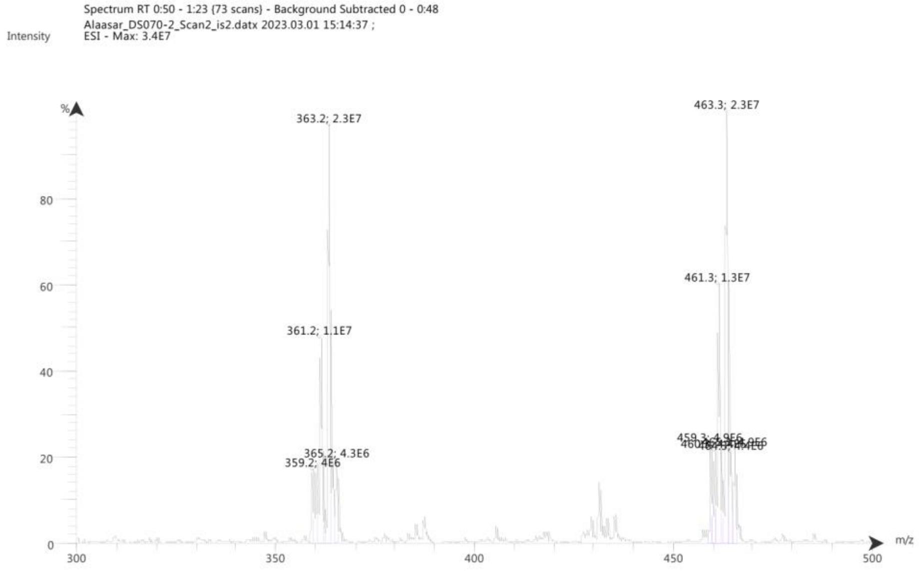

MS chart of compound **12**

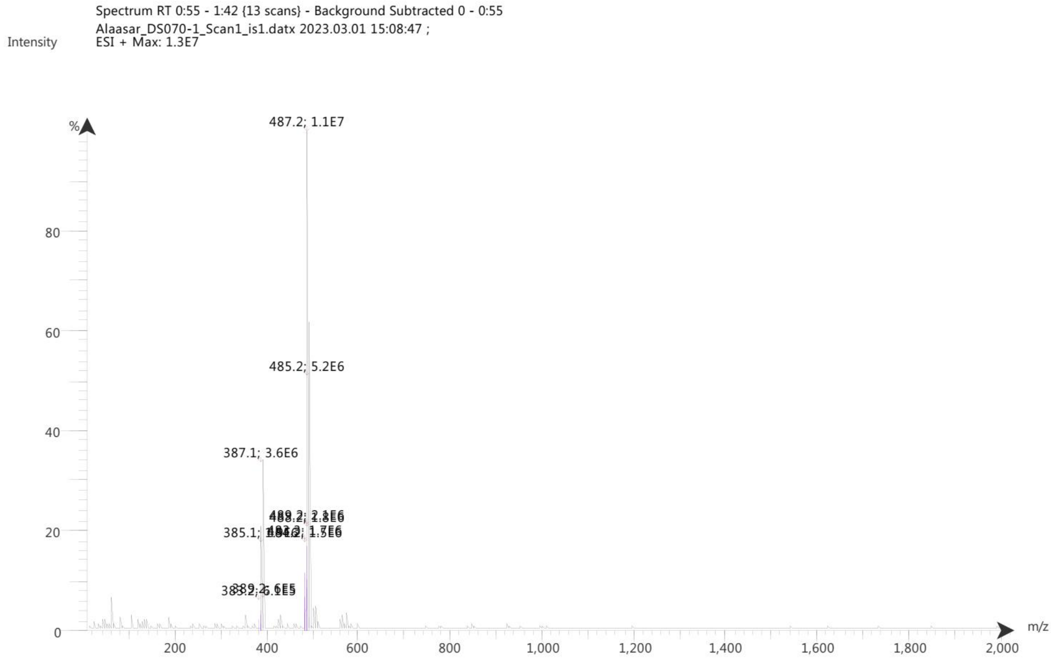

MS chart of compound **12**

SI2. Anti-inflammatory markers assay (Enzyme-linked Immunosorbent Assay)

The microplate provided in this kit has been pre-coated with an antibody specific to COX-2, IL-6, and IL-1β. Standards or samples are then added to the appropriate microplate wells with a biotin-conjugated antibody specific to COX-2, IL-6, and IL-1β. Next, Avidin conjugated to Horseradish Peroxidase (HRP) is added to each microplate well and incubated. After the TMB substrate solution is added, only those wells that contain COX-2, IL-6, and IL-1β, biotin-conjugated antibody, and enzyme-conjugated Avidin will exhibit a color change. The enzyme-substrate reaction is terminated by the addition of sulphuric acid solution and the color change is measured spectrophotometrically at a wavelength of 450 nm ± 10 nm. The concentration of COX-2, IL-6, and IL-1β in the samples is then determined by comparing the O.D. of the samples to the standard curve. Average the duplicate readings for each standard, control, and sample, and subtract the average zero standard optical density. Construct a standard curve by plotting the mean O.D. and concentration for each standard and draw a best-fit curve through the points on the graph or create a standard curve on log-log graph paper with COX-2, IL-6, and IL-1β concentration on the y-axis and absorbance on the x-axis. Using some plot software, for instance, Curve Expert 1.30, is also recommended. If samples have been diluted, the concentration read from the standard curve must be multiplied by the dilution factor.

Procedure

1. Determine wells for diluted standard, blank, and sample (**3a**, **3b**, **3c**, or **5a**). Prepare 7 wells for standard, 1 well for blank. Add 100 μL each of dilutions of standard, blank, and samples into the appropriate wells. Cover with the Plate sealer. Incubate for 1 h at 37 ^oC.

2. Remove the liquid from each well, don't wash.

3. Add 100 μL of Detection Reagent A working solution to each well, cover the wells with the plate sealer, and incubate for 1 h at 37 ^oC.

4. Aspirate the solution and wash with 350 μL of 1× Wash Solution to each well using a squirt bottle, multi-channel pipette, manifold dispenser, or autowasher, and let it sit for 1~2 min. Remove the remaining liquid from all wells completely by snapping the plate onto absorbent paper. Totally wash 3 times. After the last wash, remove any remaining Wash Buffer by aspirating or decanting. Invert the plate and blot it against absorbent paper.

5. Add 100 μL of Detection Reagent B working solution to each well, cover the wells with the plate sealer, and incubate for 30 min at 37 ^{o}C.

6. Repeat the aspiration/wash process for a total of 5 times as conducted in step 4.

7. Add 90 μL of Substrate Solution to each well. Cover with a new Plate sealer. Incubate for 10-20 min at 37 ^{o}C (Don't exceed 30 min). Protect from light. The liquid will turn blue with the addition of a Substrate Solution.

8. Add 50 μL of Stop Solution to each well. The liquid will turn yellow with the addition of the stop solution. Mix the liquid by tapping the side of the plate. If the color change does not appear uniform, gently tap the plate to ensure thorough mixing.

9. Remove any drop of water and fingerprint on the bottom of the plate and confirm there is no bubble on the surface of the liquid. Then, run the microplate reader and conduct measurement at 450 nm immediately.

SI3. Molecular dynamic simulations

The molecular dynamic simulations were carried out using the Desmond simulation package of Schrödinger LLC.[5-7] The NPγT ensemble with the temperature 300 K and a pressure 1.01 bar was applied in all runs. The simulation length was 200 ns with a relaxation time of 1 ps. The OPLS3 force field parameters were used in all simulations.[8] The cutoff radius in Coulomb interactions was 9.0 Å. The orthorhombic periodic box boundaries were set 10 Å away from the protein atoms. The water molecules were explicitly described using the transferable intermolecular potential with the three points (TIP3P) model.[9] Salt concentration was set to 0.15 M NaCl and was built using the System Builder utility of Desmond. The Martyna−Tuckerman−Klein chain coupling scheme with a coupling constant of 2.0 ps was used for the pressure control and the Nosé−Hoover chain coupling scheme for the temperature control.[10-11] Nonbonded forces were calculated using a RESPA integrator where the short-range forces were updated every step, and the long-range forces were updated every three steps. The trajectories were saved at 300 ps intervals for analysis. The behavior and interactions between the ligands and protein were analyzed using the Simulation Interaction Diagram tool implemented in the Desmond MD package. The stability of MD simulations was monitored by looking at the RMSD of the ligand and protein atom positions as a function of simulation time.

SI4. MD trajectory analysis and prime MM-GBSA calculations

Simulation interactions diagram panel of Maestro software was used to monitoring interactions contribution in the ligand-protein stability. The molecular mechanics generalized born/solvent accessibility (MM – GBSA) was performed to calculate the ligand binding free energies and ligand strain energies for docked compounds over the last 50 ns with thermal_mmgbsa.py python script provided by Schrodinger which takes a Desmond trajectory file, splits it into individual snapshots, runs the MM-GBSA calculations on each frame, and outputs the average computed binding energy.

References

1. Al-Abdallah, B.; Al-Faiyz, Y. S.; Shaaban, S., Anticancer, Antimicrobial, and Antioxidant Activities of Organodiselenide-Tethered Methyl Anthranilates. *Biomolecules* **2022,** *12* (12), 1765.
2. Sak, M.; Al-Faiyz, Y. S.; Elsawy, H.; Shaaban, S., Novel organoselenium redox modulators with potential anticancer, antimicrobial, and antioxidant activities. *Antioxidants* **2022,** *11* (7), 1231.
3. Shaaban, S.; Al-Faiyz, Y. S.; Alsulaim, G. M.; Alaasar, M.; Amri, N.; Ba-Ghazal, H.; Al-Karmalawy, A. A.; Abdou, A., Synthesis of new organoselenium-based succinanilic and maleanilic derivatives and in silico studies as possible SARS-CoV-2 main protease inhibitors. *Inorganics* **2023,** *11* (8), 321.
4. Al-Abdallah, B.; Al-Faiyz, Y. S.; Shaaban, S., Organoselenocyanates Tethered Methyl Anthranilate Hybrids with Promising Anticancer, Antimicrobial, and Antioxidant Activities. *Inorganics* **2022,** *10* (12), 246.
5. Bowers, K. J.; Chow, D. E.; Xu, H.; Dror, R. O.; Eastwood, M. P.; Gregersen, B. A.; Klepeis, J. L.; Kolossvary, I.; Moraes, M. A.; Sacerdoti, F. D.; Salmon, J. K.; Shan, Y.; Shaw, D. E. In *Scalable Algorithms for Molecular Dynamics Simulations on Commodity Clusters*, SC '06: Proceedings of the 2006 ACM/IEEE Conference on Supercomputing, 11-17 Nov. 2006; 2006; pp 43-43.
6. El-Shershaby, M. H.; Ghiaty, A.; Bayoumi, A. H.; Al-Karmalawy, A. A.; Husseiny, E. M.; El-Zoghbi, M. S.; Abulkhair, H. S., From triazolophthalazines to triazoloquinazolines: A bioisosterism-guided approach toward the identification of novel PCAF inhibitors with potential anticancer activity. *Bioorganic & Medicinal Chemistry* **2021,** *42*, 116266.
7. Research, D. E. S. *Desmond Molecular Dynamics System*, New York, NY, 2021.
8. Harder, E.; Damm, W.; Maple, J.; Wu, C.; Reboul, M.; Xiang, J. Y.; Wang, L.; Lupyan, D.; Dahlgren, M. K.; Knight, J. L.; Kaus, J. W.; Cerutti, D. S.; Krilov, G.; Jorgensen, W. L.; Abel, R.; Friesner, R. A., OPLS3: A Force Field Providing Broad Coverage of Drug-like Small Molecules and Proteins. *Journal of Chemical Theory and Computation* **2016,** *12* (1), 281-296.
9. Jorgensen, W. L.; Chandrasekhar, J.; Madura, J. D.; Impey, R. W.; Klein, M. L., Comparison of simple potential functions for simulating liquid water. *Journal of Chemical Physics* **1983,** *79* (2), 926-935.
10. Martyna, G. J.; Klein, M. L.; Tuckerman, M., Nosé–Hoover chains: The canonical ensemble via continuous dynamics. *Journal of Chemical Physics* **1992,** *97* (4), 2635-2643.
11. Martyna, G. J.; Tobias, D. J.; Klein, M. L., Constant pressure molecular dynamics algorithms. *Journal of Chemical Physics* **1994,** *101* (5), 4177-4189.
